# V- and V_L_-scores unveil viral signatures and origins of protein families

**DOI:** 10.1038/s41467-026-72028-0

**Published:** 2026-04-28

**Authors:** Kun Zhou, James C. Kosmopoulos, Etan Dieppa Colón, Peter John Badciong, Karthik Anantharaman

**Affiliations:** 1 State Key Laboratory of Marine Geology, Tongji University, Shanghai, China; 2 Department of Bacteriology, University of Wisconsin–Madison, Madison, WI USA; 3 Microbiology Doctoral Training Program, University of Wisconsin–Madison, Madison, WI USA; 4 Department of Integrative Biology, University of Wisconsin–Madison, Madison, WI USA; 5 Department of Data Science and AI, Wadhwani School of Data Science and AI, Indian Institute of Technology Madras, Chennai, India

**Keywords:** Protein databases, Bacteriophages, Microbial ecology, Comparative genomics, Statistical methods

## Abstract

Viruses are key drivers of microbial ecology and evolution, yet their study is hindered due to challenges in culturing. Traditional gene-centric methods, which focus on a few hallmark genes like for capsids, miss much of the viral genome, leaving key viral proteins and functions undiscovered. Here, we introduce two powerful annotation-free metrics, V-score and V_L_-score, designed to quantify the “virus-likeness” of protein families and genomes and create an open-access searchable database, ‘V-Score-Search’. By applying V- and V_L_-scores to public protein databases, we link 19 − 59% of protein families with viruses representing a 5 − 8x increase over current estimates. These metrics outperform existing approaches, enabling high efficiency in detection of viral genomes, prophages, and host-derived auxiliary viral genes (AVGs) from fragmented sequences. Remarkably, we identify up to 17 times more AVGs dominated by non-metabolic proteins of unknown function. This innovation unlocks new insights into virus signatures and host interactions, with wide-ranging implications from genomics to biotechnology.

## Abstract (web summary)

Zhou et al. introduce two powerful, annotation-free metrics for viral genomics: V-score and V_L_-score. These are designed to quantify the “virus-likeness” of protein families and genomes and advance viral ecology, evolution, and biotechnology.

## Introduction

Viruses are indispensable components of the biosphere. By their sheer abundance in microbiomes and ecosystems and their high genetic diversity^1^, viruses have the ability to regulate populations^2^, facilitate nutrient cycling^3^, promote genetic diversity^4^, and drive virus-host co-evolutionary dynamics^5^. In spite of their importance, viruses are difficult to culture in the laboratory, resulting in a vast number remaining unknown. Computational approaches serve as valuable complementary tools and are frequently employed for studying viral genomes and proteins. Understanding viral genomes and proteins is crucial for grasping their diversity and understanding their roles in ecosystems. This knowledge advances biotechnological applications like vaccines and phage therapy.

Traditionally, virus-specific genes, including hallmark genes such as for capsid proteins, have been considered the definitive signatures of viral genomes and used for identifying and characterizing viral genomes^6–8^. However, hallmark genes account for a small portion of viral genomes^9^. Genome or metagenome fragments often do not contain hallmark genes, making it difficult to identify and classify viruses using traditional gene-centric approaches. As a result, many viral genomes remain unidentified, leading to a significant loss of information and a growing recognition of the need to overcome these limitations in viral discovery and protein annotation.

Annotating viral genes and predicting their functions provide clues about the nature of viral sequences and protein families. We reasoned that analyzing entire viral genomes, even when fragmented, with functional annotations could break convention and yield innovative viral signatures. Here, we introduce the concepts of V-scores and V_L_-scores that are quantitative metrics to serve as a virus-like signature for differentiating between viral and non-viral protein families and genomes. V-scores were first introduced as part of the VIBRANT^10^ (Virus Identification By iteRative ANnoTation) software in 2020. While VIBRANT successfully utilized V-scores as a core metric for virus identification, achieving superior precision (99.01%) and accuracy (99.74%) compared to other tools, the scores themselves remained embedded within VIBRANT’s internal code and were not independently accessible to researchers. In this work, we have systematically recomputed, extensively benchmarked, and publicly released V-scores and newly developed logarithmically-scaled V_L_-scores for five major protein databases (KEGG, Pfam, eggNOG, PHROG, and VOG). By making these metrics openly available through our searchable database (https://anantharamanlab.github.io/V-Score-Search/), we enable diverse applications beyond VIBRANT’s original implementation. Our comprehensive analysis demonstrates that these scores provide powerful solutions for multiple viral research challenges, including precise viral sequence identification, improved prophage detection, and accurate auxiliary metabolic gene (AMG) annotation. The following results illustrate how V-scores and V_L_-scores significantly outperform existing methodologies by providing a universal metric for viral protein recognition that circumvents traditional limitations of hallmark gene-based approaches.

## Results

### Assessment of protein families for virus-like proteins

**Fig. 1:**
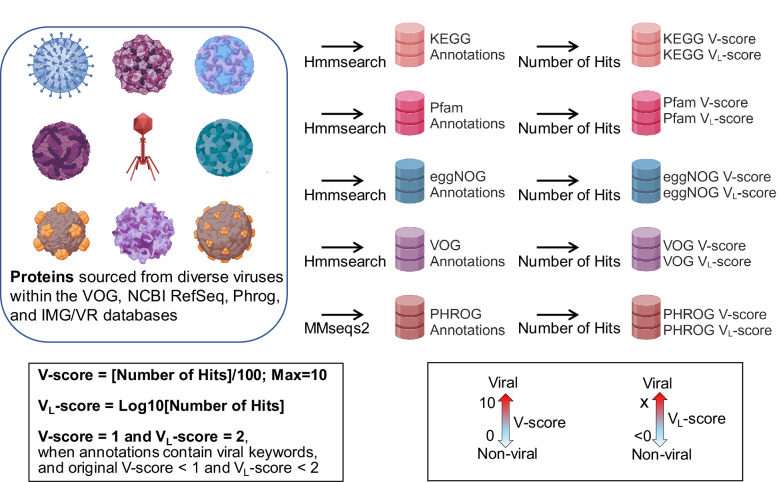
Concepts of V-score and V_L_-score. The upper section of the figure illustrates the workflow of V-score and V_L_-score generation. Nine representatives of viral taxa are shown here for the diverse viruses used in the study. A scale for V-scores and V_L_-scores is displayed by two-sided arrows going from 0 to 10 and <0 to X, respectively, suggesting low scores indicate non-viral and high-scores indicate viral. Illustration created with BioRender.com.

To establish meaningful thresholds for viral protein identification, we conducted systematic analysis of protein families across viral and non-viral sequences. Towards this, we sought to establish links between viruses and protein families (i.e., clusters of similar proteins represented under a single annotation in databases, which includes proteins of unknown function) within public annotation databases. We initially generated a non-redundant set of 18,435,589 protein sequences from various viruses (see Methods for details on sequence generation). Subsequently, we mapped protein families from diverse databases (KEGG, Pfam, eggNOG, VOG, PHROG) to these 18.4 million viral proteins to construct associations between viruses and protein families in public protein databases (Fig. 1). Each protein family in these five databases was assigned scores of viral associations that we term as V-score and V_L_-score. V- and V_L_-scores were computed by analyzing metrics of virus association when the protein family had significant numbers of hits to the 18.4 million viral proteins (see details in Methods and in Supplementary Data 1−5). For instance, the integrase protein family (KEGG KO: K14059) had 76,260 hits. To calculate the V-score, this number of hits was divided by 100, with the result capped at a maximum of 10. Therefore, the V-score for K14059 is 10. For the V_L_-score, the number of hits was scaled down using the base-10 logarithm without a maximum limit, resulting in a V_L_-score of 4.88 for K14059. To ensure that known viral structural proteins are not overlooked, we implemented a manual adjustment for protein families containing explicit viral keywords (e.g., “phage”, “capsid”, “tail”). These families were assigned a minimum V-score of 1 and V_L_-score of 2 (Fig. 1).

**Fig. 2:**
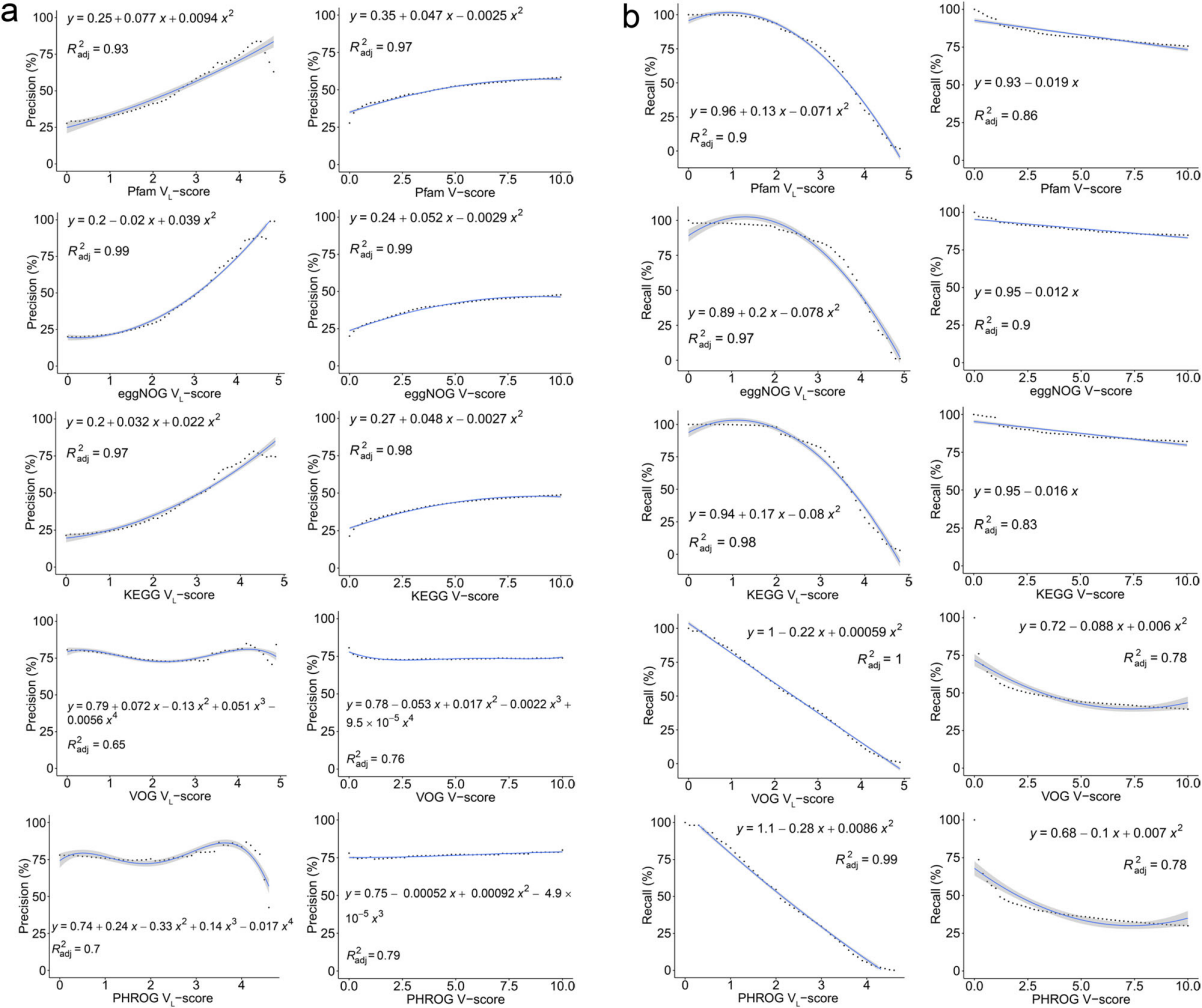
Polynomial regression analysis of V-scores and V_L_-scores. **a** Relationship between V/V_L_-scores and virus-likeness. High precision, defined as a high ratio of viral proteins identified relative to prokaryotic proteins, is achieved when the V/V_L_-scores for Pfam, KEGG, and eggNOG exceed a specific threshold. Consequently, proteins with higher precision scores exhibit greater virus-likeness. **b** Relationship between V/V_L_-scores and recall. High recall indicates a high ratio of retrieved actual viral proteins.

V-score and V_L_-score are simple numerical measures that describe how “virus-like” a protein or a group of proteins is, based on how often similar proteins are found in viruses compared to other organisms. While both scores estimate the likelihood that a protein family is of viral origin, our benchmarking across the VOG, PHROG, KEGG, Pfam, and eggNOG databases reveals distinct performance profiles for each (Fig. 2). Benchmarking demonstrates that high V_L_-scores provide high precision in identifying true viral proteins (Fig. 2a) but result in relatively low recall (Fig. 2b). In contrast, high V-scores maintain high recall, successfully retrieving a larger portion of true viral proteins, but at the cost of lower precision (Fig. 2a). These results indicate that the V_L_-score is the more effective metric for high-confidence identification of virus-specific protein families. Specially, our analysis identifies key operational thresholds for determining protein origin: high V_L_-scores (>4) serve as reliable indicators of virus-specific protein families, whereas low V_L_-scores (<2) typically suggest a non-viral origin (Fig. 2a). Notably, for the VOG and PHROG databases, which are composed exclusively of viral proteins, the precision remained consistently near 70% across all V/V_L_-score cutoffs. This result indicates that any significant match to these databases, regardless of the specific score, carries a high likelihood of viral origin. These findings confirm that V-scores and V_L_-scores are reliable, robust metrics for determining the virus-likeness of protein families across both general and virus-specific databases.

**Fig. 3:**
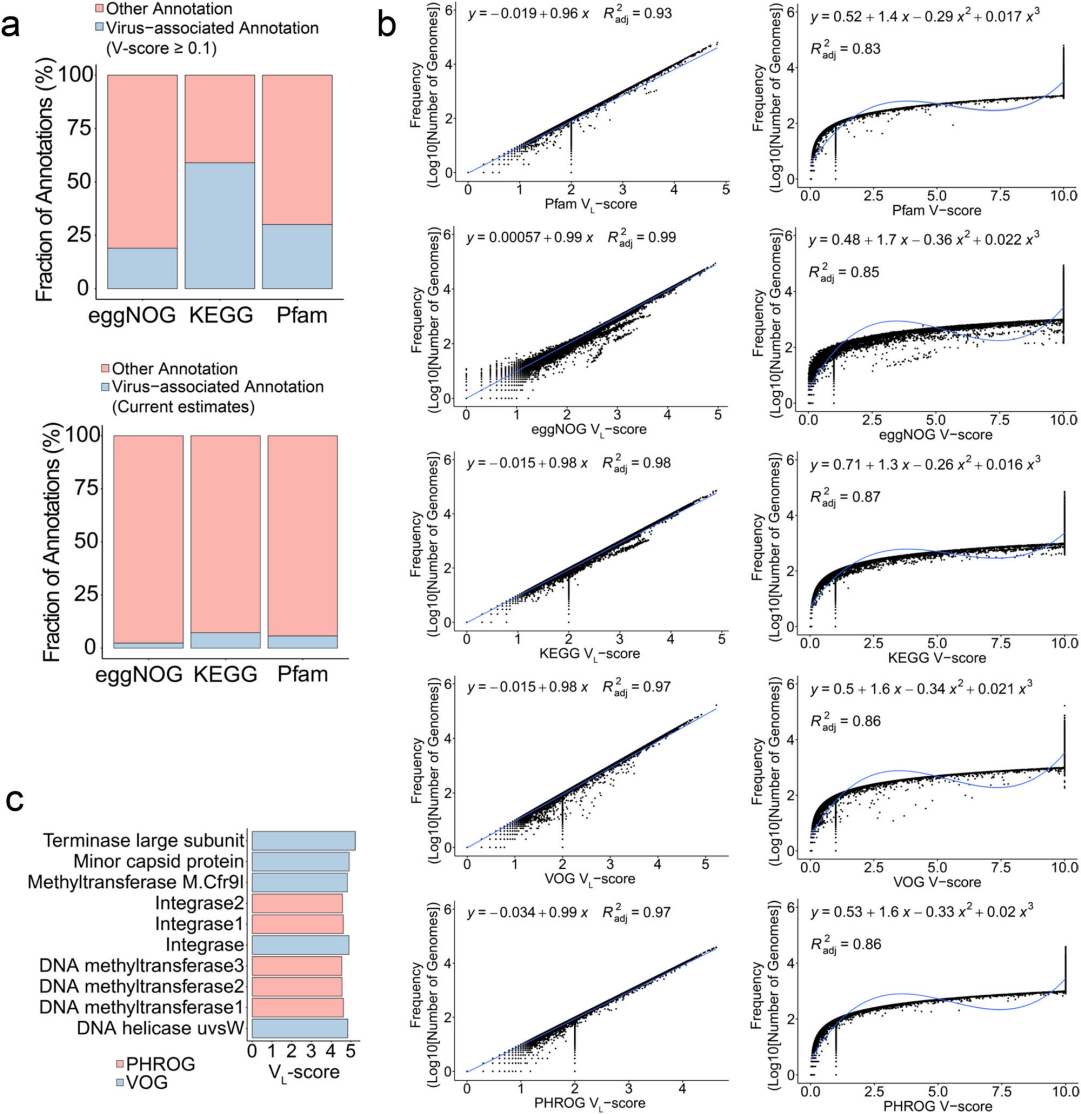
Distribution of viral protein families. **a** Frequency of virus-associated annotations with V-score ≥0.1 and in current estimates. **b** Correlation between V/V_L_-scores and protein family prevalence across viral genomes. The x-axis represents the observed V/V_L_-scores for annotated protein families, while the y-axis indicates the total count of viral genomes sharing those specific annotations. Blue lines indicate the predicted values derived from the polynomial regression model. **c** Top five annotations associated with viruses based on V_L_-scores.

Through polynomial regression analysis of the distribution patterns of these V-scores and V_L_-scores across the 18.4 million protein sequences from various sources (see Methods for details on sequence generation), we determined significant statistical relationships between V- and V_L_-scores and viral association probability. We identified cutoffs of V-score = 0.1 and V_L_-score = 1 (indicating 10 hits to the database of 18.4 million viral proteins) to define association with viral proteins. High V-scores and V_L_-scores indicated a strong association with viral proteins, whereas low scores suggested a weaker association. Protein families associated with viral proteins constituted ~59%, 30%, and 19% of the total protein families in KEGG (15,339), Pfam (6250), and eggNOG (66,301), respectively (Fig. 3a). In contrast, current estimates of viral protein entries in KEGG, Pfam, and eggNOG are limited, representing a very small fraction (<10%) (Fig. 3a). These percentages of association such as 59% for KEGG do not imply that 59% of all proteins in KEGG are viral. Rather, they indicate that 59% of KEGG protein families contain at least 10 viral members (e.g., a family with 10,000 bacterial proteins and 10 viral proteins would qualify). Our analysis increases the number of protein families in public databases associated with viruses and significantly improves the overall representation of viral proteins in these databases. This increase in viral representation will facilitate better understanding of viral roles in ecosystems, their interactions with hosts, and their evolutionary dynamics.

Next, we hypothesized that the associative nature of V-scores and V_L_-scores could also reflect the frequencies of protein families in viral communities, thereby serving as a metric for quantifying both prevalence and specificity. Towards this, we used the protein families documented in the databases of Pfam, KEGG, eggNOG, PHROG, and VOG to analyze their distribution across viral genomes. We determined that V-scores and V_L_-scores correlate significantly with the observed frequency of these families; specifically, higher scores indicate widespread distribution, while lower scores correspond to infrequent distribution (Fig. 3b). For example, according to PHROG and VOG V_L_-scores, methyltransferase-coding genes were frequently distributed in viral communities (Fig. 3c), which was also evidenced by the high V_L_-scores for these protein families in KEGG, Pfam, and eggNOG (e.g., KEGG V_L_-score = 4.8 and Pfam V_L_-score = 4.7). Consequently, the V/V_L_-score could serve as a measure of commonality; a high V/V_L_-score means the protein family is commonly seen in viruses, while a low V/V_L_-score means it is rare or uncommon in viruses. This approach will allow for the identification of proteins commonly encountered on viruses but whose function is currently not known. In contrast, protein families with very low V-scores and V_L_-scores, e.g., host-derived proteins, metabolic proteins, and hypothetical proteins with V-scores of 0.01, indicated the presence of viral proteins that are rare in communities and may confer specialized functions more likely to be involved in niche-specific interactions^11^.

### Calculation of the average V-scores and V_L_-scores of genomes for viral differentiation and prediction

To build upon our understanding of V-scores and V_L_-scores from the protein to genome-scales, we posited that the association and frequency of V-scores and V_L_-scores may confer features on viral genomes that distinguish them from other organisms. To test this, we investigated a whole-genome catalog of 5800 viral, 50,523 plasmid, and 4813 prokaryotic genomes and calculated the average V-score (AV-score) and average V_L_-score (AV_L_-score) for each genome (See methods for details) (Supplementary Fig. S1). We proposed that AV- and AV_L_-scores represented the average V- or V_L_-scores of protein families across an entire genome and would thus be representative of the overall virus-like character of a given genome. We determined that prokaryotic viruses had significantly higher medians of AV-scores (3.602−9.515) and AV_L_-scores (1.802−3.830) compared to plasmids and prokaryotic chromosomes regardless of annotation databases (*p*-value < 10^−5^; Supplementary Fig. S1a). Interestingly, viral genome fragments (1–15 kb) extracted from whole genomes also displayed significantly higher medians (see examples of KEGG and Pfam AV-scores and AV_L_-scores in Supplementary Figs. S2, S3, respectively). The higher median scores for viral genomes suggest that this metric could capture features unique to viruses, making it highly effective for identifying viral genomes in mixed communities such as metagenomes of viruses, plasmids, and chromosomes. To validate this, we conducted polynomial regression analyses on the fraction of viral genomes within mixed metagenomes containing viruses, plasmids, and chromosomes at various cutoffs of AV-scores and AV_L_-scores for both whole genomes and genome fragments (Supplementary Data 6−9). At the whole-genome level, the fraction of viral genomes increased with higher AV-score and AV_L_-scores (for VOG) (Supplementary Fig. S1b). Similarly, at the fragment level, the fraction of viral genomes increased with higher AV-score cutoffs for KEGG and Pfam (Supplementary Figs. S4, S5). From regression analyses (Supplementary Fig. S1b), whole genomes with AV-scores/AV_L_-scores exceeding the corresponding cutoffs (e.g., a VOG AV-score of 2, which surpasses the VOG AV-score cutoff of 1.93) were predicted to be viral with a 70% probability (likely viral) or a 90% probability (most likely viral) (see detailed cutoffs in Supplementary Data 10). For genome fragments, only the AV-scores of VOG, PHROG, KEGG, and Pfam were able to generate cutoffs predictive of viral genomes with a 70% or 90% probability (Supplementary Figs. S4−7). Given that cutoffs may vary with fragment size, different cutoffs were established for corresponding sizes (Supplementary Data 10). Overall, the concepts of AV-scores and AV_L_-scores offer novel insights into genome signatures, traditionally defined by k-mer frequency^12^ or single-copy signature genes^13^. The cutoffs for AV-scores and AV_L_-scores, used to differentiate between viral and non-viral genomes, may prove valuable for viral identification in metagenomic studies. Overall, these metrics address limitations of conventional gene-centric and alignment-dependent methods^8,10,14,15^.

### Maximizing identification of viral genomes

To evaluate the potential of AV-scores and AV_L_-scores for applications in metagenomics, we first assessed the false discovery rate (FDR) of the AV-score-based approach using a dataset of 711,495 prokaryotic fragments and 1137 viral fragments. We employed four approaches (see Methods and Materials) to assess the FDR and identify optimal strategies. For each approach, we performed a bootstrap analysis with 1000 replicates, randomly selecting 100 prokaryotic and 100 viral fragments in each replicate. Across the 1000 replicates, the lowest median false positive rates were 5%, achieved by the AV-score combined with CheckV assessment (Supplementary Fig. S8). This approach, using a fragment length cutoff of 5 kb, also generated the lowest median false negative rate of 9% (Supplementary Fig. S8).

**Fig. 4:**
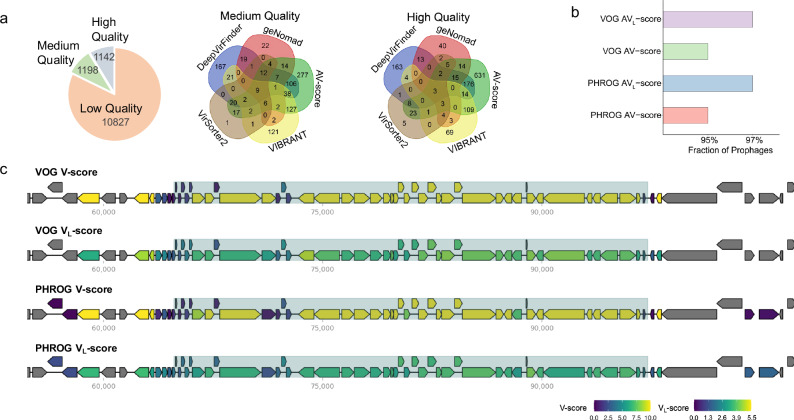
Application of V-scores, V_L_-scores, AV-scores, and AV_L_-scores for viral identification in genomes and metagenomes. **a** Number of sequences identified with AV-scores and AV_L_-scores and four commonly used software. For medium- and high-quality sequences ≥5 kb, as assessed by CheckV, the overlap between the five approaches was illustrated using Venn diagrams, showing the number of shared and specific sequences identified by different methods. **b** Fraction of prophages in a database that have AV-scores and AV_L_-scores above corresponding cutoffs for viral-like determination. **c** Distribution of V-scores and V_L_-scores for genes within a verified *E. coli* prophage and its adjacent host sequences. Prophage regions are shaded for emphasis.

These results suggest that combining AV-score and CheckV assessment is an optimal strategy for identifying viral genomes. Consequently, we analyzed a dataset of 39 host-associated metagenomes using the AV-score combined with CheckV assessment, applying fragment length cutoffs of either 1 or 5 kb. By applying AV-score cutoffs (with a 70% probability of being viral) for genome fragments of varying sizes, derived from KEGG, Pfam, VOG, or PHROG, we identified 13,167 viral sequences (contigs ≥1 kb) of low, medium, and high quality (Fig. 4a). Of these, 2045 sequences overlapped with those identified using geNomad which is a virus identifier dependent on virus-specific markers^8^ (Supplementary Fig. S9a). While our approach identified fewer low-quality viral sequences (10,827) compared to geNomad (17,369), it significantly outperformed geNomad in identifying medium- and high-quality sequences. Specifically, the AV-score-based approach identified over 1000 high-quality viral sequences, which is approximately seven times more than geNomad detected (Supplementary Fig. S9b).

Additionally, the AV-score-based method surpassed other conventional tools, including machine learning-dependent DeepVirFinder^16^, VIBRANT^10^, a hybrid approach incorporating machine learning and protein similarity, and VirSorter2^15^, a multi-classifier based approach, in identifying medium- and high-quality sequences ≥5 kb (Fig. 4a). Moreover, compared to previous studies on sponge-associated microbiomes^17,18^, we identified 129 viral sequences of medium or higher quality, more than 15 times the number of viral genomes (7 sequences) previously predicted using VirSorter^7^. Most of the high-quality viral genomes identified by the AV-score approach are specific to AV-score, indicating that this method can uncover viral genomes that other tools may not recognize (Fig. 4a). These findings suggest that the usage of AV-scores and AV_L_-scores can detect many viral sequences that traditional, viral-specific gene-dependent methods may overlook. Overall, the application of AV-scores and AV_L_-scores as metrics for genome differentiation offers a novel and powerful tool for identifying viral genomes in metagenomic studies.

We further tested the potential of this approach for prophage identification and assessment. The results showed that over 95% of sequences in a prophage database used by a popular prophage identification tool, PHASTER^19^ (65,668 prophages), had AV-scores and AV_L_-scores above our suggested cutoffs for whole genomes (70% probability, based on VOG and PHROG scores) (Fig. 4b). Additionally, clear boundaries between a verified *Escherichia coli* prophage and its adjacent host sequences were delineated by relatively low V-scores and V_L_-scores using VOG and PHROG (Fig. 4c). Furthermore, the higher AV-scores observed for VOG, PHROG, Pfam, KEGG, and eggNOG families in prophages (see Supplementary Fig. S10) strongly support the idea that AV-scores and/or AV_L_-scores are useful in identifying prophage boundaries when combined with sliding window approaches (e.g., a 10 kb sliding window^20^). Accurately predicting prophage boundaries has long been a challenge^21,22^, possibly due to the presence of auxiliary metabolic genes (AMGs) in phages^23,24^ or the ability of phages to be transposable and encode serine integrases rather than tyrosine integrases^21^. Given their ability to distinguish viral from non-viral genes and sequences, AV-scores, AV_L_-scores, and V_L_-scores may offer highly precise methods for boundary recognition.

### Advancing the identification of auxiliary genes in viral genomes

Despite recent efforts, the vast majority of viral proteins (>80%) have no known function which has hindered our understanding of the roles of viruses in ecosystems and microbiomes. V-scores and V_L_-scores as quantitative metrics display a property of measuring the frequency of individual protein families among viral genomes in public databases. Leveraging this property through the development of hidden Markov models for protein families, we assessed their effectiveness in identifying AVGs, including AMGs on viral genomes. AVGs are virus-encoded genes of prokaryotic origin that are not essential for viral propagation processes such as genome replication, lysis, or capsid assembly, while AMGs are auxiliary genes that are associated with metabolic roles^25^. Such genes likely provide a fitness benefit to the virus encoding them^25–27^. Identifying AVGs is a particularly difficult problem compounded by host-associated contamination and the host-derived nature of these genes. Given their importance due to the increasing recognition of auxiliary genes involved in human and environmental microbiomes^27–31^, we investigated whether V-scores and V_L_-scores could effectively identify auxiliary genes.

**Fig. 5:**
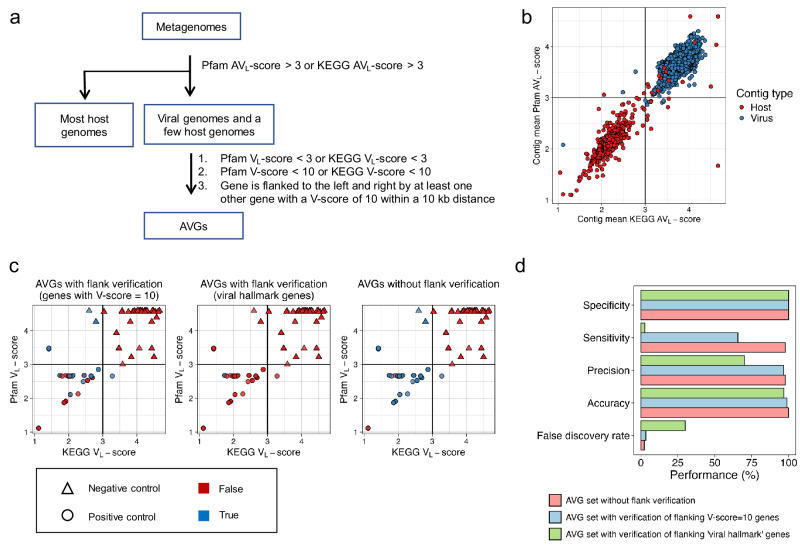
Application of V-scores, V_L_-scores, and AV_L_-scores to AVG detection. **a** Workflow for AVG identification. **b** Establishing the optimal Pfam/KEGG AV_L_-score of query scaffolds to distinguish viral vs. host genomes. Points represent individual genes, plotted by the AV_L_-score of all Pfam or KEGG annotations encoded by the gene’s origin scaffold. Vertical and horizontal lines represent the chosen scaffold AV_L_-score used to distinguish viral from host scaffolds (>3: virus, <3: host). Points are colored by the actual scaffold type of the gene’s origin (host or virus). **c** Establishing optimal Pfam V_L_-score/KEGG V_L_-score combinations to distinguish viral auxiliary vs. non-auxiliary genes. Points represent individual genes in our database of viral and host genomes that had both Pfam^5^ and KEGG^6^ annotations matching to either the database of the 17 AMGs or 10 non-AMG protein families. Genes marked as potentially auxiliary have a maximum KEGG and Pfam V_L_-scores of 3, as indicated by the vertical and horizontal lines. **d** Performance of the proposed AMG identification workflow. Performance was evaluated based on the confusion matrices in Supplementary Data 12. Definitions for specificity, sensitivity, precision, accuracy, and false discovery rate are provided in Supplementary Data 12.

To test this hypothesis, we developed a workflow (Fig. 5a) and evaluated the ability of V-scores, V_L_-scores, and AV_L_-scores to identify experimentally verified AMGs. We first averaged the V_L_-scores of all KEGG or Pfam protein families across entire scaffolds, establishing a scaffold Pfam/KEGG AV_L_-score of 3 as optimal for differentiating viral from host scaffolds (Fig. 5b). We then distinguished AMGs from host-encoded metabolic genes and non-auxiliary genes by using V-scores and V_L_-scores (Fig. 5c). Our workflow effectively detected AMGs (Fig. 5d and exemplified in Supplementary Fig. S11). We achieved a sensitivity of 97.71% and a false positivity rate of 2.29% using a database of biochemically characterized AMGs (experimentally verified) for benchmarking (see details in Supplementary Data 11). Community standards for analyzing AMGs recommend verifying that a virally encoded AMG is flanked both upstream and downstream by hallmark genes^32,33^. This check ensures that metabolic genes identified from proviral sequences are not in regions of host contamination, however, this standard hinders AMG recall for non-proviruses. This is because hallmark genes, which are used for viral identification, constitute only a small fraction of viral genomes and genome or metagenome fragments frequently lack these hallmark genes. The requirement for verification significantly reduced sensitivity to 66% (when verified with genes having V-scores of 10) and to 2.67% (when verified with hallmark genes), while also increasing the false discovery rate to 30% when using hallmark gene verification (Fig. 5d, Supplementary Data 11, 12). The ability of V-scores and V_L_-scores to confidently identify viral proteins circumvents the need to identify hallmark proteins. Therefore V-scores offer a novel methodology for verifying that AMGs encoded by proviruses are not the result of host contamination.

**Fig. 6:**
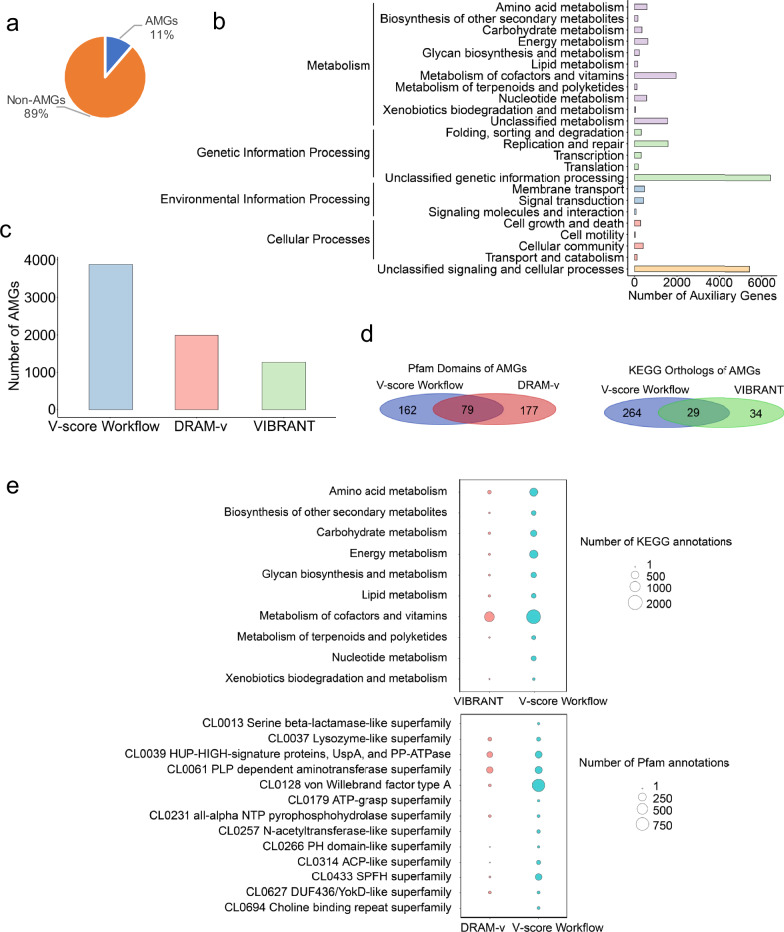
Auxiliary genes identified in the study and comparison with existing methods. **a** Auxiliary gene composition. AMG: auxiliary metabolic genes. **b** Potential functions of auxiliary genes with annotations detected using the V-score workflow. Purple bars represent categories within Metabolism, green bars denote Genetic Information Processing, blue bars indicate Environmental Information Processing, pink bars correspond to Cellular Processes, and orange bars represent unclassified signaling and cellular processes. **c** Number of AMGs identified by the V-score workflow compared to other existing methods, including DRAM-v and VIBRANT. **d** Overlap and unique Pfam domains or KEGG orthologs of AMGs identified by the V-score workflow, DRAM-v, and VIBRANT. **e** Comparison of the number of KEGG or Pfam annotations of AMGs identified using the V-score workflow, DRAM-v, and VIBRANT. Please note that VIBRANT exclusively outputs results that contain KEGG annotations, while DRAM-v mainly generated Pfam annotations for AMGs identified in the study.

Leveraging this advantage, we were able to predict a significantly larger number of auxiliary genes from 5,116 high-confidence viral genomes (of high quality), providing deeper insights into viral functions. Our workflow (with verified flanking genes with V-score = 10) identified a total of 27,442 viral genes likely to be auxiliary and the workflow without verification predicted 34,015 auxiliary genes (4.85% of all viral genes in our test dataset and 16.50% of all annotated viral genes) (Supplementary Data 13). Notably, non-metabolic AVGs comprise a substantial majority, accounting for 89%, while auxiliary metabolic genes represent a small subset, making up only 11% (Fig. 6a). The identified AVGs included genes encoding various metabolic enzymes, antibiotic resistance proteins, transporters, DNA/RNA replication proteins, transposases/recombinases, nucleases/endonucleases, and uncharacterized/hypothetical proteins. These AVGs serve diverse functions including metabolism, genetic information processing, environmental information processing, and cellular processes (Fig. 6b; Supplementary Data 13). Some of the genes have been considered auxiliary, for example, the genes encoding D-3-phosphoglycerate dehydrogenase for carbon metabolism^23^, S-adenosylmethionine decarboxylase for amino acid metabolism^34^, and alpha-L-fucosidase for glycan degradation^35^. Notably, our study predicted numerous auxiliary genes that were typically overlooked in previous studies of auxiliary genes. For instance, over 700 viral auxiliary genes related to toxin-antitoxin systems were identified. These systems, which are typically used by hosts as a defense mechanism against viral infections^36,37^, may be employed by viruses to enhance their ability to infect host organisms^36,38,39^, contributing to viral evolution in the ongoing virus-host arms race. Additionally, the presence of many genes with unknown functions suggests that there are still numerous unexplored roles for viruses, likely with important ecosystem or microbiome contexts.

In comparison to other existing approaches, our workflow significantly outperformed widely used approaches including VIBRANT^10^ and DRAM-v^32^, as demonstrated by the identification of AMGs. When applied to the same set of viral genomes, our V-score workflow identified 3859 AMGs (Fig. 6c; Supplementary Data 13), while VIBRANT and DRAM-v identified only 1261 and 1993 AMGs, respectively (Fig. 6c; Supplementary Data 14, 15). Notably, only a small fraction of Pfam domains or KEGG orthologs of AMGs were commonly identified by three approaches (Fig. 6d), with most AMGs being unique to each method. This suggests that our V-score workflow reveals novel functions that are often overlooked by existing AMG detection tools. Some unique metabolic enzymes uncovered by our method include the serine beta-lactamase-like superfamily (Pfam clan accession: CL0013), ATP-grasp superfamily, N-acetyltransferase-like superfamily, and Choline binding repeat superfamily (Fig. 6e). Furthermore, our workflow outperformed VIBRANT, as shown by the higher number of AMGs identified across all KEGG categories (Fig. 6d). Collectively, these findings demonstrate that the V-score-based approach can detect a greater number of potential AVGs with high precision.

## Discussion

In conclusion, V-scores and V_L_-scores represent powerful quantitative metrics that describe the virus-like nature and origin of protein families and genomes. Usage of V-scores and V_L_-scores offers a promising approach for the differentiation between viral and non-viral proteins, extending beyond simple gene presence or absence and incorporating quantitative assessment. Such metrics could be particularly useful in cases where traditional methods struggle, such as in distinguishing viral genes embedded within plasmids^40^ or identifying viral elements within bacterial genomes^41,42^. Additionally, these quantitative metrics for protein families are also able be applied for the differentiation of viral and non-viral genome sequences using combined V_L_-scores or V-scores across different proteins. These metrics can serve as the foundation of new tools to advance viral genomics, ecology, and evolutionary analyses. By enabling open and public distribution of these scores ((https://anantharamanlab.github.io/V-Score-Search/), we propose that they will propagate broadly in microbiology. Our approach allows for citation of these scores using databases identifiers like for KEGG, Pfam etc or using protein annotations. For example, a picornavirus capsid protein (PF00073) has a V-score of 10 implying a strong virus association while a Hepatitis C virus capsid protein (PF01543) has a V-score of 1 implying a weaker virus association, presumably because its proteins domains are not specific to capsids.

The versatility of these scores allows for their incorporation into diverse genomics tools such as for virus identification in complex datasets and identification of AMGs. By integrating metrics like AV-scores and AV_L_-scores, researchers could develop more refined tools for viral identification, potentially leading to the discovery of novel viral genomes and a deeper understanding of virus-host interactions. The broader implication of this approach is that it allows for more nuanced and data-driven differentiation between viral and non-viral entities at both the gene and genome levels. This could revolutionize how we identify and characterize viruses in complex biological systems, offering new insights into viral evolution, diversity, and function.

The quantitative nature of the metrics also opens up possibilities for automating and scaling viral genome study across large datasets, for example the completeness assessment of linear viral genomes in cases where identifiable terminal repeats are absent^6^, making it an invaluable resource in the field of viral (meta)genomics. Furthermore, these metrics offer a novel framework for characterizing signatures of population differentiation. Characterizing new viral species in complex systems is crucial for understanding how microbial interactions impact the spread of diseases and their development and impact on health^43^. AV-scores and AV_L_-scores capture the association and frequency of viral genomes, as well as their differentiation from other genomes. An analysis of 11 viral species from the NCBI RefSeq database revealed that closely related species share similar AV-scores or AV_L_-scores, whereas distinct species exhibit significantly different score profiles (Supplementary Fig. S12). This highlights the possibility of these metrics for viral genome classification and identification of novel species. For instance, changes in the gut phage population have been repeatedly linked to various gastrointestinal diseases^44–46^. The application of AV-scores or AV_L_-scores into gut phage population studies would provide opportunity to differentiate viral populations in complex host-associated systems and contribute to uncover certain disease-related viral species.

While V-scores, V_L_-scores, AV-scores, and AV_L_-scores provide novel and quantitative frameworks for viral proteins, several limitations and potential biases should be acknowledged. First, the accuracy of V-scores is inherently dependent on the composition and quality of the underlying viral protein databases. Current databases are often biased toward cultured viruses and well-studied environments, which may lead to overrepresentation of certain viral groups and under-detection of novel or uncultivated viruses. This database dependency could result in inflated V-scores for proteins from well-characterized viruses and reduced sensitivity for those from less-studied lineages. To address this, we are committed to regularly updating V-scores as viral databases expand, thus ensuring our metrics reflect the growing diversity of viral sequences.

Another limitation arises from the lack of comprehensive, experimentally validated databases delineating provirus-host boundaries, which can complicate the precise identification of proviral regions and may contribute to false positives in AVG detection. To mitigate this, we recommend coupling V-score analyses with established provirus identification tools such as VIBRANT, CheckV, or geNomad to filter out host contamination before proceeding with AMG detection. Additionally, there is an inherent trade-off between sensitivity and specificity in our pipeline, particularly when using high V-score thresholds for flanking genes to reduce false positives. While this approach limits the detection of AMGs in regions lacking hallmark genes, we propose a modified workflow that initially identifies AMGs without flank verification, followed by provirus validation and refined AVG prediction to better balance sensitivity and specificity.

It is also important to note that V-scores cannot always distinguish between viral proteins acquired from hosts (AMGs) and host-like proteins retained through shared evolutionary history, highlighting the need for complementary analyses such as genomic context or evolutionary rate comparisons. Furthermore, as viral databases evolve and expand, V-score thresholds and protein family associations may shift, especially with the inclusion of metagenome-derived viral sequences. We explicitly recognize this dynamic nature and are committed to providing regular updates to V-scores in tandem with major database releases to maintain reproducibility and accuracy. Finally, we encourage community-driven efforts to refine V-score calculations, expand databases, and experimentally validate AMGs, and we have made our pipelines and datasets openly accessible to facilitate ongoing improvements. By transparently addressing these limitations and inviting collaboration, we aim to ensure that our methodology remains robust, current, and broadly useful to the research community.

## Methods

### Viral protein database construction

Viral protein sequences were downloaded from public databases (accessed January 2024), including the National Center for Biotechnology Information (NCBI) RefSeq database, the Virus Orthologous Groups (VOG) database (version 221, https://fileshare.csb.univie.ac.at/vog/), the Prokaryotic Virus Remote Homologous Groups (PHROG) database^47^, and the IMG/VR Viral Resources^48^ (version 4.1). Protein sequences of high-confidence viral genomes from IMG/VR Viral Resources were filtered and we only retained high-quality and medium-quality viral sequences that were assessed by CheckV^49^ (version 1.0.13). To dereplicate proteins, MMseqs2 linclust^50^ (version 13.45111) was used with an identity cutoff of 95% (custom parameters: --min-seq-id 0.95 --cluster-mode 2 --cov-mode 1 -c 1.0), and generated non-redundant 18,435,589 protein sequences.

### Annotation profile database selection

To construct a wide range of associations between annotation profiles and viral proteins, a diverse collection of profile databases was selected. The profile databases included Kyoto Encyclopedia of Genes and Genomes (KEGG) KOfam^51^ (version 2024-01-01) that is a customized Hidden Markov Models (HMMs) profile collection of KEGG Orthologs, Pfam-A (release 36.0)^52^ database of a large collection of diverse protein families, and eggNOG^53^ (version 5.0) that is a database of non-supervised orthologs created from a large number of various organisms. Two additional curated viral ortholog collections are the VOG (release 221, vogdb.org) and PHROG both of which were constructed based on remote homology.

### V-score and V_L_-score generation

The V-score and V_L_-score for each annotation profile within the KEGG, Pfam, eggNOG, PHROG, and VOG databases were determined based on the number of significant hits (*E*-value ≤ 10^−5^). These hits were identified using hmmsearch^54^ (HMMER 3.4) for the KEGG, Pfam, eggNOG, and VOG databases, and MMseqs2 specifically for the PHROG database. All searches were conducted against our constructed database of 18,435,589 protein sequences, as illustrated in Fig. 1. For V-score, the resulting number was divided by 100, with a maximum limit set at 10 after division. For V_L_-score, the resulting number was scaled down using the common logarithm (base 10) without a maximum limit. In the case of annotations containing viral keywords including “virus”, “viral”, “phage”, “portal”, “terminase”, “spike”, “capsid”, “sheath”, “tail”, “coat”, “virion”, “lysin”, “holin”, “base plate”, “lysozyme”, “head”, “structural”, or “Viral protein families”, protein families/annotations were assigned adjusted V-score of 1 and V_L_-score of 2 if the original V-score was less than 1 and V_L_-score less than 2. Each annotation profile is given a V-score and a V_L_-score, serving as metrics for virus association. It is important to note that the V-scores do not consider virus specificity or association with non-viruses and have been manually adjusted to prioritize viral hallmark genes. To maintain consistency and comparability over time, V-scores and V_L_-scores were normalized by the total number of proteins in the constructed database (18,435,589 sequences).

To evaluate the performance of our approach, we conducted additional analyses comparing the V-scores ≥0.01 and V_L_-scores ≥0 derived from databases constructed using RefSeq and VOG (cultured viruses only) versus those derived from databases using VOG, RefSeq, PHROG, and IMG/VR4 (including high-confidence uncultivated viruses). When we applied V- and V_L_-scores to public annotation databases such as KEGG, Pfam, and eggNOG, the results revealed that the inclusion of high-confidence uncultivated viruses significantly expanded the number of links between protein families and viruses:

- Using only cultured virus databases including RefSeq and VOG, we linked 14–50% of public protein families to viruses, representing a 4–7× increase compared to current estimates.
- When incorporating high-confidence viruses from IMG/VR4 and PHROG, the linkage improved to 38–77%, representing an 8–17× increase compared to current estimates.

Overall, both approaches (cultured-only vs cultured + high-confidence uncultivated) significantly improved viral annotations compared to existing databases. However, the inclusion of high-confidence uncultivated viruses from IMG/VR4 further enhanced the ability of the V- and V_L_-scores to detect viral signatures, creating more associations between viruses and protein families, without substantially increasing the risk of incorporating non-viral sequences.

In summary, while we recognize potential concerns regarding biases introduced through the use of predicted viromes like IMG/VR4, our careful selection of “high-confidence” viral genomes ensures the reliability of our approach. Importantly, our method demonstrates that incorporating these high-confidence uncultivated viruses provides a more comprehensive view of viral biodiversity and expands associations between viral proteins and public annotations, which would otherwise remain unlinked if only “cultured” viral databases like RefSeq and VOG (cultured viruses only) were used. We hope this explains our decision and demonstrates our confidence in the robustness of the constructed viral protein database.

### Calculation of the frequency of protein families in viruses

To quantify the prevalence of protein families within the viral domain, we calculated V-scores and V_L_-scores for each annotation profile across the KEGG, Pfam, eggNOG, PHROG, and VOG databases. Significant hits were identified by searching the protein profiles against our integrated database of 18,435,589 viral sequences using hmmsearch (HMMER 3.4) and MMseqs2 with an *E*-value threshold of 10^−5^. For each annotation, the V-score and V_L_-score were derived from the total number of unique viral genomes associated with these significant hits. To characterize the relationship between the observed genome counts and the resulting scores, we performed a polynomial regression analysis in R. The best-fit curves were generated using a model (method: “lm”) and visualized using the tidyverse, ggplot2, and ggpubr libraries.

### Database construction of chromosomes, plasmids, and viral genomes

Databases of prokaryotic chromosomes, plasmid sequences, and prokaryotic viral genomes were constructed for the calculation of AV-score and AV_L_-score. Prokaryotic genomes (release 214) were downloaded from the Genome Taxonomy Database (GTDB; gtdb.ecogenomic.org)^55,56^. We assessed the quality of each genome with a quality score (score = completeness − 5 × contamination − 0.05 × no. scaffolds)^8^, genomes of each GTDB family with the highest quality score were selected as family representatives to reduce computational load and taxonomic bias. As a result, 4304 bacterial and 509 archaeal genomes were selected to be used in the following analyses. Then, provirus and provirus-like sequence regions were identified with VirSorter2 (version 2.2.4; --min-length 5000) and VIBRANT (version 1.2.1 with default parameters) and removed from the selected prokaryotic genomes. Additionally, plasmid sequences (sequence headers containing the word “plasmid”) were removed from the selected prokaryotic genomes. For plasmids and prokaryotic viruses, 50,523 plasmid sequences were downloaded from the PLSDB database^57^ (version 2023_11_23) and viral genomes were downloaded from the NCBI RefSeq database^58^ (retrieved in January 2024). To retrieve prokaryotic viral genomes, the GenBank division PHG was used to filter bacterial and archaeal viruses in the RefSeq database. Finally, 5800 genomes of prokaryotic viruses were retained.

### Benchmarking precision and recall for V-score and V_L_-score

Protein sequences for all the 4304 bacterial and 509 archaeal genomes and the 5800 prokaryotic viral genomes were predicted using Prodigal^59^ (v2.6.3; -m -p meta). The predicted protein datasets were used to label known viral proteins and protein families. Functional annotations were assigned as follows:

- KEGG, VOG, and Pfam: Identified via Hmmsearch^54^ (HMMER 3.4, parameter: *E*-value ≤ 10^−5^)
- eggNOG: Annotated using EggNOG-mapper^60^ (version 2.1.12; parameters: -m mmseqs --evalue 10^-5^).
- PHROG: Searched using MMseqs2 (“created”, “search”, and “createtsv” modules; *E*-value ≤ 10^−5^), retaining only the top-scoring hit for each protein.

V-scores and V_L_-scores from the KEGG, VOG, eggNOG, Pfam, and PHROG databases were assigned to each protein based on their respective functional annotations. To evaluate the performance of these metrics, a benchmarking dataset consisting of 536,805 viral proteins (positives) and 12,159,906 prokaryotic proteins (negatives) was established. Precision and recall were calculated across a gradient of thresholds: V-score cutoffs ranged from 0 to 10 (0.2 increments), and V_L_-score cutoffs ranged from 0 to 5 (0.1 increments). For each specific threshold, proteins exceeding the cutoff were classified as True Positives (TP) if they were of viral origin and False Positives (FP) if they were of prokaryotic origin. Viral-origin proteins below the cutoff were classified as False Negative (FN). Precision and recall for each cutoff were then determined as follows:

1 $${{{\rm{Precision}}}}=\frac{{{{\rm{TP}}}}}{{{{\rm{TP}}}}+{{{\rm{FP}}}}}\times 100\%$$

2 $${{{\rm{Recall}}}}=\frac{{{{\rm{TP}}}}}{{{{\rm{TP}}}}+{{{\rm{FN}}}}}\times 100\%$$

The relationship between score thresholds and precision/recall was modeled using polynomial regression (linear model smoothing) in R, using the ggplot2, ggpubr, and tidyverse libraries to determine the best-fit curves.

### Generation of average V-score and V_L_-score for genome sequences

Databases of prokaryotic chromosomes, plasmids, and prokaryotic viruses constructed above were used to calculate the AV-score and AV_L_-score for each genome. Each whole genome of prokaryotic viruses, plasmids, and chromosomes were randomly split into non-overlapping, non-redundant genome fragments at length from 1 to 15 kb. This approach was implemented using custom Python scripts to simulate metagenome-assembled sequences. Proteins of each whole genome and split genome fragment were predicted using Prodigal^59^ (version 2.6.3; parameters: -m -p meta). Hmmsearch^54^ (HMMER 3.4, parameter: -E 10^−5^) was used to match the proteins of prokaryotic viruses, plasmids, prokaryotes to the HMM profiles of KEGG, VOG, and Pfam. EggNOG-mapper^60^ (version 2.1.12; parameters: -m mmseqs --evalue 10^−5^) was used to annotate the proteins with the eggNOG database. The “created”, “search”, and “createtsv” modules of MMseqs2 were employed to search the predicted proteins against the PHROG database using an *E*-value threshold of 10^−5^. Only the hit with the highest score was kept. Then V-score and V_L_-score of KEGG, VOG, eggNOG, Pfam, and PHROG were assigned to each protein. For comparison between viruses, plasmids, and chromosomes, AV-score and AV_L_-score were calculated for each whole genome and genome fragment. The AV-score and AV_L_-score of KEGG, Pfam, and eggNOG were expressed as:

3 $${{{\rm{AV}}}}-{{{\rm{score}}}}=\frac{\sum {{{\rm{V}}}}-{{{\rm{score}}}}{{{\rm{s}}}}\;{{{\rm{of}}}}\;{{{\rm{proteins}}}}\; {{{\rm{with}}}}\;{{{\rm{significant}}}}\;{{{\rm{hits}}}}}{{{{\rm{Number}}}}\; {{{\rm{of}}}}\; {{{\rm{proteins}}}}\; {{{\rm{with}}}}\; {{{\rm{significant}}}}\; {{{\rm{hits}}}}}$$

4 $${{{{\rm{AV}}}}}_{{{{\rm{L}}}}}-{{{\rm{score}}}}=\frac{\sum {{{{\rm{V}}}}_{{{\rm{L}}}}}-{{{\rm{scores}}}}\; {{{\rm{of}}}}\; {{{\rm{proteins}}}}\; {{{\rm{with}}}}\; {{{\rm{significant}}}}\; {{{\rm{hits}}}}}{{{{\rm{Number}}}}\; {{{\rm{of}}}}\; {{{\rm{proteins}}}}\; {{{\rm{with}}}}\; {{{\rm{significant}}}}\; {{{\rm{hits}}}}}$$

The AV-score and AV_L_-score of PHROG and VOG were expressed as:

5 $${{{\rm{AV}}}}-{{{\rm{score}}}}=\frac{\sum {{{\rm{V}}}}-{{{\rm{scores}}}}\;{{{\rm{of}}}}\;{{{\rm{proteins}}}}\; {{{\rm{with}}}}\;{{{\rm{significant}}}}\;{{{\rm{h}}}}{{{\rm{its}}}}}{{{{\rm{Total}}}}\; {{{\rm{number}}}}\; {{{\rm{of}}}}\; {{{\rm{proteins}}}}\; {{{\rm{encoded}}}}\; {{{\rm{in}}}}\; {{{\rm{a}}}}\; {{{\rm{genome}}}}}$$

6 $${{{{\rm{AV}}}}}_{{{{\rm{L}}}}}-{{{\rm{score}}}}=\frac{\sum {{{{\rm{V}}}}_{{\mbox{L}}}}-{{{\rm{scores}}}}\; {{{\rm{of}}}}\; {{{\rm{proteins}}}}\; {{{\rm{with}}}}\; {{{\rm{significant}}}}\; {{{\rm{hits}}}}}{{{{\rm{Total}}}}\; {{{\rm{number}}}}\; {{{\rm{of}}}}\; {{{\rm{proteins}}}}\; {{{\rm{encoded}}}}\; {{{\rm{in}}}}\; {{{\rm{a}}}}\; {{{\rm{genome}}}}}$$

### Generation of cutoffs of AV-score and AV_L_-score for viral-like genome determination

The constructed dataset, comprising prokaryotic viruses, plasmids, and chromosomes along with their AV-scores and AV_L_-scores, was used to investigate the relationship between the viral fraction in the dataset and the corresponding AV-score and AV_L_-score ranges. We extracted over 40 sub-datasets from this comprehensive dataset, with each sub-dataset having a maximum AV-score of 10 or an AV_L_-score of 5. The minimum AV-scores ranged from 0 to 10 in increments of 0.2, while the minimum AV_L_-scores ranged from 0 to 5 in increments of 0.1. To calculate the fraction of viral sequences in each sub-dataset, we first normalized the numbers of viral genomes (Nv), plasmids (Np), and chromosomes (Nc). The viral fraction was then computed using the formula:

7 $${{{\rm{Viral\; Fraction}}}}=\frac{{Nv}}{{Nv}+{Np}+{Nc}}$$

Where:

8 $${Nv}=\frac{{{{\rm{Number}}}}\; {{{\rm{of}}}}\; {{{\rm{viral}}}}\; {{{\rm{sequences}}}}\; {{{\rm{in}}}}\; {{{\rm{the}}}}\; {{{\rm{sub}}}}-{{{\rm{dataset}}}}}{{{{\rm{Total}}}}\; {{{\rm{number}}}}\; {{{\rm{of}}}}\; {{{\rm{viral}}}}\; {{{\rm{sequences}}}}\; {{{\rm{in}}}}\; {{{\rm{the}}}}\; {{{\rm{dataset}}}}}$$

9 $${Np}=\frac{{{{\rm{Number}}}}\; {{{\rm{of}}}}\; {{{\rm{plasmid}}}}\; {{{\rm{sequences}}}}\; {{{\rm{in}}}}\; {{{\rm{the}}}}\; {{{\rm{sub}}}}-{{{\rm{dataset}}}}}{{{{\rm{Total}}}}\; {{{\rm{number}}}}\; {{{\rm{of}}}}\; {{{\rm{plasmid}}}}\; {{{\rm{sequences}}}}\; {{{\rm{in}}}}\; {{{\rm{the}}}}\; {{{\rm{dataset}}}}}$$

10 $${Nc}=\frac{{{{\rm{Number}}}}\; {{{\rm{of}}}}\; {{{\rm{chromosome}}}}\; {{{\rm{sequences}}}}\; {{{\rm{in}}}}\; {{{\rm{the}}}}\; {{{\rm{sub}}}}-{{{\rm{dataset}}}}}{{{{\rm{Total}}}}\; {{{\rm{number}}}}\; {{{\rm{of}}}}\; {{{\rm{chromosome}}}}\; {{{\rm{sequences}}}}\; {{{\rm{in}}}}\; {{{\rm{the}}}}\; {{{\rm{dataset}}}}}$$

The probability of a genome being viral was represented by the fraction of normalized viral genomes. The minimum AV_L_-scores and AV-scores for the sub-datasets were designated as cutoffs for identifying viral-like sequences. It was observed that as these cutoffs increased, indicating higher AV_L_-scores and AV-scores, the likelihood of the sequences being viral also correspondingly increased. Polynomial regression with the smoothing method “lm” was used to predict the best-fit curve that matches the pattern of the cutoff and probability by using R (R library ggpubr, palmerpenguins, tidyverse, repr, and ggplot2). The cutoffs for the probability of 70% and 90% were predicted according to estimated polynomial regression equations. If a genome sequence has a score above the cutoff for the probability of 70%, this sequence was determined as a “likely” viral-like sequence. If a genome sequence has an AV-score/AV_L_-score above the cutoff for the probability of 90%, this sequence was determined as a “most likely” viral-like sequence. The process for establishing the relationship between cutoffs and probability is illustrated in Supplementary Fig. S13.

### Applying cutoffs to the identification of viral sequences

To evaluate the AV-score’s performance and false discovery rate, we used a dataset comprised of 84 archaeal and 1472 bacterial genomes (100% completeness, 0% contamination; from GTDB) and 110 complete phage genomes (from NCBI RefSeq). These genomes were distinct from those used for AV-score and AV_L_-score generation. We simulated metagenome-assembled sequences by randomly splitting each genome into non-overlapping fragments of 1–15 kb using custom Python scripts. This yielded 1137 viral fragments and 711,495 prokaryotic fragments, which were combined into a single database. A bootstrap analysis (1000 replicates) was performed. In each replicate, 100 prokaryotic and 100 viral sequences were randomly sampled from the database. Subsequently, false discovery rates, encompassing both false negative and false positive rates, were calculated for each replicate using the four approaches outlined below:

1. AV-score alone with a fragment length cutoff of 1 kb.
2. AV-score alone with a fragment length cutoff of 5 kb.
3. AV-score combined with CheckV^49^ (version 1.0.13) assessment and a fragment length cutoff of 1 kb.
4. AV-score combined with CheckV assessment and a fragment length cutoff of 5 kb.

False positives were defined as prokaryotic fragments erroneously identified as viral sequences by the AV-score approaches, while false negatives were viral fragments not identified as viral sequences.

To identify viral sequences, each randomly selected fragment (prokaryotic and viral) was functionally annotated using VOG, PHROG, KEGG, and Pfam databases via HMMsearch (HMMER 3.4, *E*-value ≤ 10^−5^) and MMseqs2 (*E*-value ≤ 10^−5^). AV-scores were then calculated for each sequence against each database (VOG, PHROG, KEGG, and Pfam). For the AV-score alone approaches, a fragment was predicted to be viral if at least one of its AV-scores exceeded the corresponding size-specific cutoff (e.g., a PHROG AV-score >4.24 or a VOG AV-score >4.91 for a 2.5 kb scaffold; detailed cutoffs are in Supplementary Data 10). For the AV-score combined with CheckV assessment approaches, a fragment was predicted to be viral if at least one of its AV-scores exceeded the corresponding size-specific cutoff and the fragment completeness was >0. Finally, false negative and false positive rates were calculated for each replicate and were used to identify the optimal approach with the lowest false negative and false positive rates. The results clearly indicated that the AV-score combined with CheckV assessment yielded the lowest false positive rates (Supplementary Fig. S8). Therefore, this combined approach was selected for subsequent metagenomic analysis.

Metagenomes from host-associated microbiomes were analyzed as a use case to demonstrate the application of viral genome identification. Raw Illumina reads of one snail-associated metagenome^61^, three sponge-associated metagenomes^17,18^, three human-associated metagenomes^62^, and 32 coral-associated metagenomes^63^ were retrieved from NCBI (BioProject accessions: PRJNA612619 for snail, PRJNA552185 for sponge, PRJNA763232 for human, PRJNA574146 for coral). The reads were then trimmed using Trimmomatic^64^ (version 0.36) with custom settings (ILLUMINACLIP: TruSeq3-PE.fa:2:30:10 LEADING:3 TRAILING:3 SLIDINGWINDOW:4:15 MINLEN:40). Trimmed reads from the sponge-, human-, and snail-associated microbiomes were assembled with MEGAHIT^65^ (version 1.2.9) using default parameters, while reads from coral-associated microbiomes were assembled using SPAdes^66^ (version 3.11.1) with custom settings (--meta, k-mer sizes varied from 51 to 91, with a 10-mer step size). The assembled metagenomes (contigs ≥1 kb) were then functionally annotated using VOG, PHROG, KEGG, and Pfam via Hmmsearch (HMMER 3.4, parameter: -E 10-5) and MMseqs2 (*E*-value ≤ 10-5). AV-scores for VOG, PHROG, KEGG, and Pfam were subsequently calculated for each sequence. Predicted viral genomes were identified based on the following criteria: (1) sequences with at least one AV-score (from VOG, PHROG, KEGG, or Pfam) exceeding the corresponding cutoffs for each fragment size (e.g., a PHROG AV-score >4.24 or a VOG AV-score >4.91 for a 2.5 kb scaffold; detailed cutoffs by fragment size are provided in Supplementary Data 10). For sequences larger than 15 kb, cutoffs for 14–15 kb fragments were used. (2) Sequences meeting criterion (1) were further filtered for completeness >0%, as assessed by CheckV. In parallel, geNomad^8^ (version 1.7.411; end-to-end, contigs ≥1 kb), VirSorter2^15^ (version 2.2.3; contigs ≥5 kb, --include-groups dsDNAphage, NCLDV, RNA, ssDNA, lavidaviridae), VIBRANT^10^ (version 1.2.0; contigs ≥5 kb), and DeepVirFinder^16^ (version 1.0; contigs ≥5 kb, score ≥0.75, *p* < 0.05), were used to identify viral sequences from the host-associated metagenomes, allowing for a comparison between the V-score-based and specific gene- or hallmark- or machine learning-based viral identification methods. For consistency, viral sequences identified by geNomad, VirSorter2, VIBRANT, and DeepVirFinder were also required to have completeness >0%, as assessed by CheckV.

### Appling cutoffs to the assessment of proviral sequences

Cutoffs of AV-scores and AV_L_-scores of whole genomes in Supplementary Data 10 were used for the assessment on proviral sequences by estimating the consistency of our method with a custom prophage database. The custom prophage database developed by Arndt et al.^19^ were downloaded from PHASTER. Then prophage sequences in the database were functionally annotated with VOG and PHROG using Hmmsearch (HMMER 3.4, parameter: -E 10^−5^) and MMseqs2 (*E*-value ≤ 10^−5^), followed by the calculation of the AV-scores and the AV_L_-scores of VOG and PHROG for each prophage. Any prophage sequences with an AV-score or AV_L_-score above their corresponding cutoff were considered consistent with the prophage database.

To show a potential application in prophage boundary identification, one experimentally verified provirus, *Enterobacteria* phage P88^67^, and its host were selected and downloaded from NCBI (*E. coli* GenBank: GCA_001005685.1). Proteins of prophage and host genomes were predicted using Prodigal V2.6.3 (parameters: -m -p meta)^59^. Hmmsearch^54^ (HMMER 3.4, parameter: -E 10^−5^) was used to match the proteins of prophages and hosts to the HMM profiles of VOG. MMseqs2 with a custom parameter (*E*-value ≤ 10^−5^) was used to search prophage and host proteins against the PHROG database. Only the best hit to each protein was retained. Then V-score and V_L_-score of VOG and PHROG were assigned to each protein, followed by calculating AV-score and AV_L_-score for each prophage and adjacent host sequence. The gene feature plots of prophages were generated and visualized with DNA Features Viewer^68^.

### Database construction for benchmarking on AVG identification

We assembled a database of 17 KEGG and Pfam HMM profiles (V-scores <10 for KEGG annotations or V-scores <10 for Pfam annotations) representing AMGs experimentally demonstrated to affect host metabolism^69–73^ (Supplementary Data 16) and a database of 10 selected HMMs that represent non-AMGs (Supplementary Data 17). From IMG/VR v4^48^, we compiled a database of 5116 high-confidence viral genomes in high quality^74^ (Supplementary Data 18) containing the 17 experimentally verified AMGs, the 10 non-AMGs, and genomes with neither to obtain a representative sample. We ensured each viral genome had a known host genus, and we compiled a database of 180 host genomes (containing homologs of the 17 experimentally verified AMGs) representing the known host genera. We used geNomad^8^ v1.7.4 to predict viral scaffolds in the 180 host genomes and removed viral scaffolds binned in host genome assemblies (Supplementary Data 19).

Open reading frames in all virus and host genomes were identified and translated using pyrodigal-gv^8,59^ (version 0.3.1; github.com/althonos/pyrodigal-gv). Translated proteins were aligned to Pfam-A^52^ HMMs (version 36.0) and KEGG^75^ KO HMMs using pyhmmer^54,76^ (version 0.10.10) hmmsearch^54^ with a maximum *E*-value of 10^−5^. For proteins aligning to multiple HMM profiles within the same database, the highest scoring alignment was reported. Each protein with a Pfam or KEGG functional annotation was assigned its corresponding Pfam or KEGG V_L_-score and V-score.

### Optimal parameter determination for potential AVG detection

To develop the AVG detection pipeline, we used 5116 viral genomes and 180 host genomes from the constructed databases. We distinguished viral from most host genomes by averaging the V_L_-scores of all KEGG or Pfam annotations in entire scaffolds, establishing a minimum scaffold Pfam/KEGG AV_L_-score of 3 as optimal for differentiating viral from host scaffolds (see Fig. 5b). Thus, we required potential viral scaffolds to have an AV_L_-score >3 for Pfam/KEGG annotations.

Following this initial filtration, the resulting viral genomes and a few remaining host genomes were then used to determine parameters for detecting potential AVGs on the genome sequences. Pfam V_L_-score <3 and/or a KEGG V_L_-score <3 were identified as optimal parameters for separating AMGs from non-AMGs, as shown in Fig. 5c. We then applied these determined parameters to AMG identification, both with and without flanking verification. Our flanking verification approach involved running our AMG identification workflow using viral hallmark genes to verify flanking regions of potential AMGs. We defined viral hallmark genes in our KEGG and Pfam HMM databases as previously described^77^; any HMM profile with an annotation/description containing any of the following keywords: virion structure (truncated from *structure* to account for matches to the terms “structure” or “structural”), capsid, portal, tail, and terminase. A list of KEGG and Pfam HMMs defined as viral hallmark genes this way are provided in Supplementary Data 20. In parallel, we verified that AMGs identified with our workflow were flanked on both sides by at least one gene with a V-score of 10 within 10 kb of the AMG, recognizing that viral genes with unknown functions may still be characteristically viral. The optimal parameters effectively separated AMGs from non-AMGs, with the best performance observed without flank verification (see the “Assessment on performance of the workflow for AMG identification” section below for detailed information on how the performance was evaluated.). Flank verification using genes with a V-score of 10 provided the next best separation, followed by flank verification using hallmark genes, as depicted in Fig. 5c, d. Consequently, we stipulated that potential AVGs must have Pfam V_L_-scores <3 and/or KEGG V_L_-scores <3. To mitigate the risk of misidentifying AVGs from proviral sequences, which may introduce host contamination, we advise employing flank verification using genes with a V-score of 10. This strategy improves the precision and dependability of AVG identification by reducing the inclusion of non-viral elements.

### Assessment on performance of the workflow for AVG identification

To assess the performance of our workflow, we established positive and negative controls for AMGs in our test genome dataset. A gene encoded by a viral scaffold with an annotation in the experimentally verified AMG database was considered a positive control (Supplementary Data 16), while any host-encoded gene in the experimentally verified AMG database was considered a negative control (Supplementary Data 16). Genes encoded on viral scaffolds with annotations matching any of 10 selected HMMs that represent non-AMGs were also considered negative controls (Supplementary Data 17). Any other gene, encoded on a known host or viral genome, that was not annotated with the experimentally verified AMG database or non-AMG database was not considered a positive or negative control.

To ensure that we did not analyze viral genes in host genomes, we used geNomad to predict viral contigs (prophages) in the 180 host genomes and all genes encoded on host scaffolds predicted as viral were removed before we predicted the positives and negatives of our AMG identification workflow. Predicted positives were any gene, encoded on a known host or viral scaffold, that met the following criteria: (1) the gene has a Pfam V_L_-score <3 or a KEGG V_L_-score <3, (2) the gene has a Pfam V-score <10 or a KEGG V-score <10, (3) the gene is encoded on a scaffold with a Pfam AV_L_-score >3 or a KEGG AV_L_-score >3, (4) the gene is flanked to the left and right by at least one other gene with a V-score of 10 within a 10 kb distance (only applies to results reporting prediction “with flank verification”). Any gene with an annotation belonging to the AMG database or the non-AMG database that did not meet these criteria was considered a predicted negative. Genes without annotations to the non-AMG or the AMG database were not predicted as positives or negatives. The counts of positive controls, negative controls, predicted positives, and predicted negatives were used to construct the confusion matrices in Supplementary Data 12.

### Comparing our AVG detection workflow to existing approaches

We used the established database of 5116 high-quality viral genomes sourced from IMG/VR v4^48^ (Supplementary Data 18). All viral genes were evaluated for potential auxiliary functions using the AMG identification workflow, both with and without flank verification. Genes annotated under KEGG’s “sulfur relay system” or “metabolic pathways” category, excluding those related to nucleotide metabolism or sulfonate transport system substrate-binding proteins, were considered potential AMGs. Additionally, auxiliary genes with KEGG and PFAM annotations were cross-referenced against a viral AMG database^32^, which includes experimentally verified AMGs from previous studies^23,34,69–73,78,79^. PFAM and KEGG accessions associated with AMGs were retrieved, and ORFs containing these accessions were retained and integrated into the AMG dataset. To compare our approach with other existing tools to identify AMGs, we ran VIBRANT^10^ with the “annoVIBRANT” implementation (github.com/AnantharamanLab/annoVIBRANT) and DRAM-v^32^ (version 1.5.0) on the same set of high-quality viral genomes. For DRAM-v, only the AMGs with a score of 1 were retained, which indicates the presence of at least one hallmark gene on both sides, suggesting the gene is likely viral.

### Viral species differentiation based on AV-score and AV_L_-score

Reference prokaryotic viruses with complete genomes were used for assessment on viral population differentiation based on AV-score and AV_L_-score. Lineage of the reference viruses was downloaded from virushostdb (https://www.genome.jp/virushostdb). According to the lineage information of each viral RefSeq genome, 11 species of reference prokaryotic viruses were selected (each species with ≥4 genomes). Viral species include *Bixzunavirus Bxz1*, *Campylobacter* virus IBB35, *Fibrovirus fs1*, *Inovirus M13*, *Kayvirus G1*, *Otagovirus Psa374*, *Pegunavirus Pg1*, *Pegunavirus soto*, *Pegunavirus Suffolk*, *Restivirus RSS1*, and *Wphvirus megatron*. Viral genomes were annotated with databases of VOG, PHROG, KEGG, Pfam, and eggNOG using Hmmsearch (HMMER 3.4, parameter: -E 10^−5^), MMseqs2 (parameter: *E*-value ≤ 10^−5^), or EggNOG-mapper (version 2.1.12; parameters: -m mmseqs --evalue 10^−5^). In the following, the AV-score and AV_L_-score of each genome were calculated. Detailed information of NCBI RefSeq accessions and AV-score and AV_L_-score of viral genomes was provided in Supplementary Data 21.

### Reporting summary

Further information on research design is available in the Nature Portfolio Reporting Summary linked to this article.

## Supplementary information

Supplementary Information

Description Of Additional Supplementary Files

Supplementary Data 1

Supplementary Data 2

Supplementary Data 3

Supplementary Data 4

Supplementary Data 5

Supplementary Data 6

Supplementary Data 7

Supplementary Data 8

Supplementary Data 9

Supplementary Data 10

Supplementary Data 11

Supplementary Data 12

Supplementary Data 13

Supplementary Data 14

Supplementary Data 15

Supplementary Data 16

Supplementary Data 17

Supplementary Data 18

Supplementary Data 19

Supplementary Data 20

Supplementary Data 21

Reporting Summary

Transparent Peer Review file

## Data Availability

A database of V-scores and V_L_-scores associated with every protein cluster or family is available at https://anantharamanlab.github.io/V-Score-Search/. Additionally, the complete dataset has been archived in Zenodo and can be accessed at https://zenodo.org/records/18793981. Reference sequences utilized for evaluation can be downloaded from IMG/VR (https://genome.jgi.doe.gov/portal/IMG_VR/IMG_VR.home.html). Metagenome reads of host-associated microbiomes were retrieved from NCBI.

## Code availability

Codes used in this project are available at https://github.com/AnantharamanLab/V-Score-Search.
