# Supplementary material for: V- and VL-scores unveil viral signatures and origins of protein families
Source: Nat Commun. 2026 Apr 28;17:5828. doi: 10.1038/s41467-026-72028-0 (PMC13332234; doi:10.1038/s41467-026-72028-0)
Supplement: Supplementary file 1 — Supplementary Information [file 41467_2026_72028_MOESM1_ESM.pdf]

Supplementary Information for "V- and V_L-Scores Unveil Viral Signatures and Origins of Protein Families"

Kun Zhou[1,2,*], James C. Kosmopoulos[2,3], Etan Dieppa Colón[2,3], Peter John Badciong[2], Karthik Anantharaman[2,4,5,*]

[1]State Key Laboratory of Marine Geology, Tongji University, Shanghai, China

[2]Department of Bacteriology, University of Wisconsin–Madison, Madison, WI, USA

[3]Microbiology Doctoral Training Program, University of Wisconsin–Madison, Madison, WI, USA

[4]Department of Integrative Biology, University of Wisconsin–Madison, Madison, WI, USA

[5]Department of Data Science and AI, Wadhwani School of Data Science and AI, Indian Institute of Technology Madras, Chennai, India

*Correspondence: karthik@bact.wisc.edu, kunzhou@tongji.edu.cn

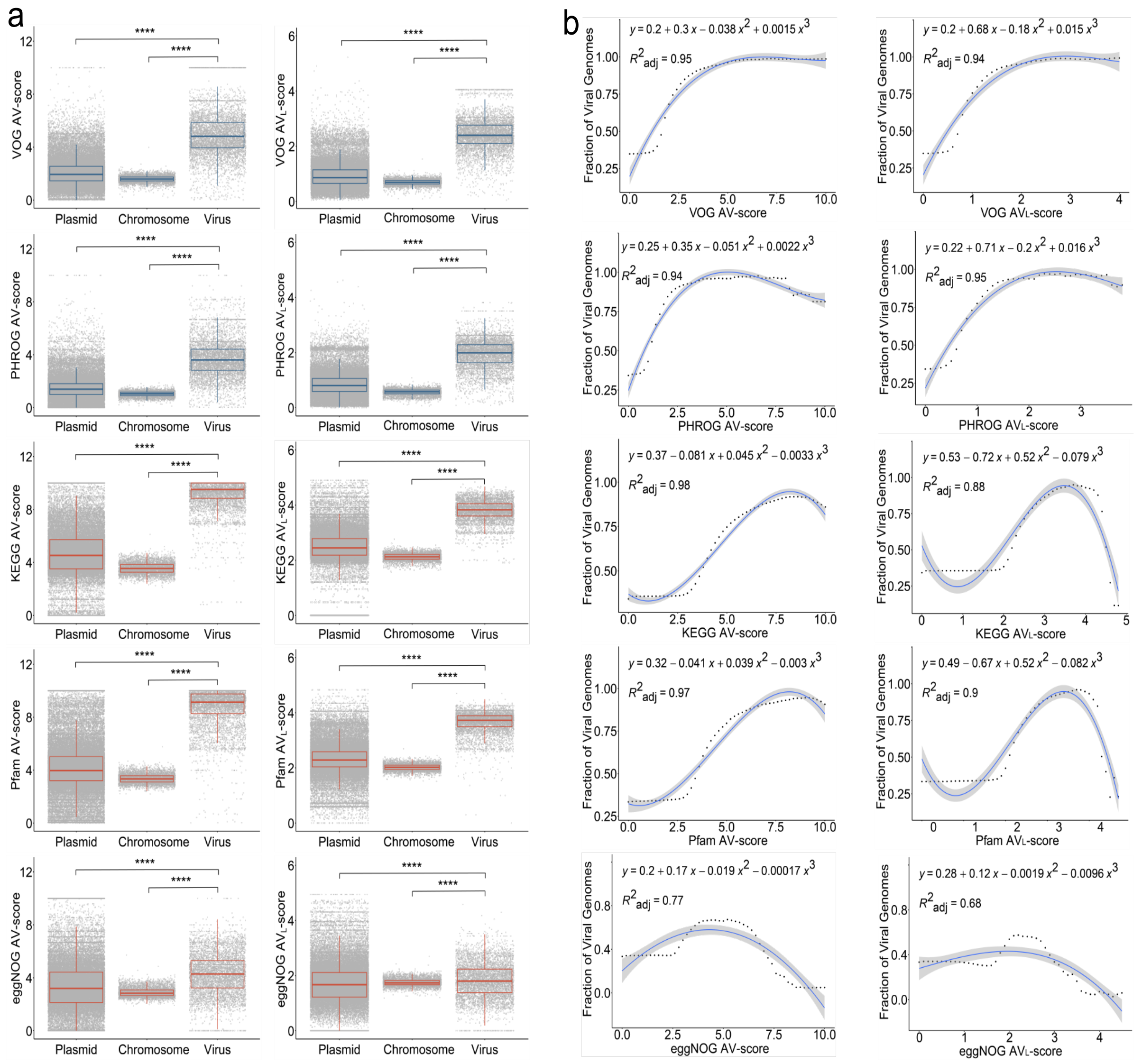

Supplementary Fig. S1 Distribution and polynomial regression analysis of AV/AV_L-scores. **a,** Distribution of AV-score and AV_L-score of prokaryotic chromosomes (*n* = 4,813) and the genomes of plasmids (*n* = 50,523) and prokaryotic viruses (*n* = 5,800). The blue boxes denote the AV-scores and AV_L-scores of VOG and PHROG. The red boxes denote the AV-scores and AV_L-scores of KEGG, Pfam, and eggNOG. The horizontal line that splits the box is the median, the upper and lower sides of the box are upper and lower quartiles, whiskers are 1.5 times the interquartile ranges and data points beyond whiskers are considered potential outliers. An ANOVA test was used to show differences between three means are significant ($p < 2.2 \times 10^{-16}$). **** denotes $p < 10^{-4}$. **b,** Relationship between the fraction of viral genomes used in (a) and the AV-scores and AV_L-scores. In this study, we define the fraction of viral genomes as the probability that a given genome sequence is viral. The dots on the dotted line represent the actual values of the fraction of viral genome sequences, while the blue lines indicate the predicted values. The process for generating the fraction of viral genome sequences is exemplified in Supplementary **Supplementary Fig. S13**.

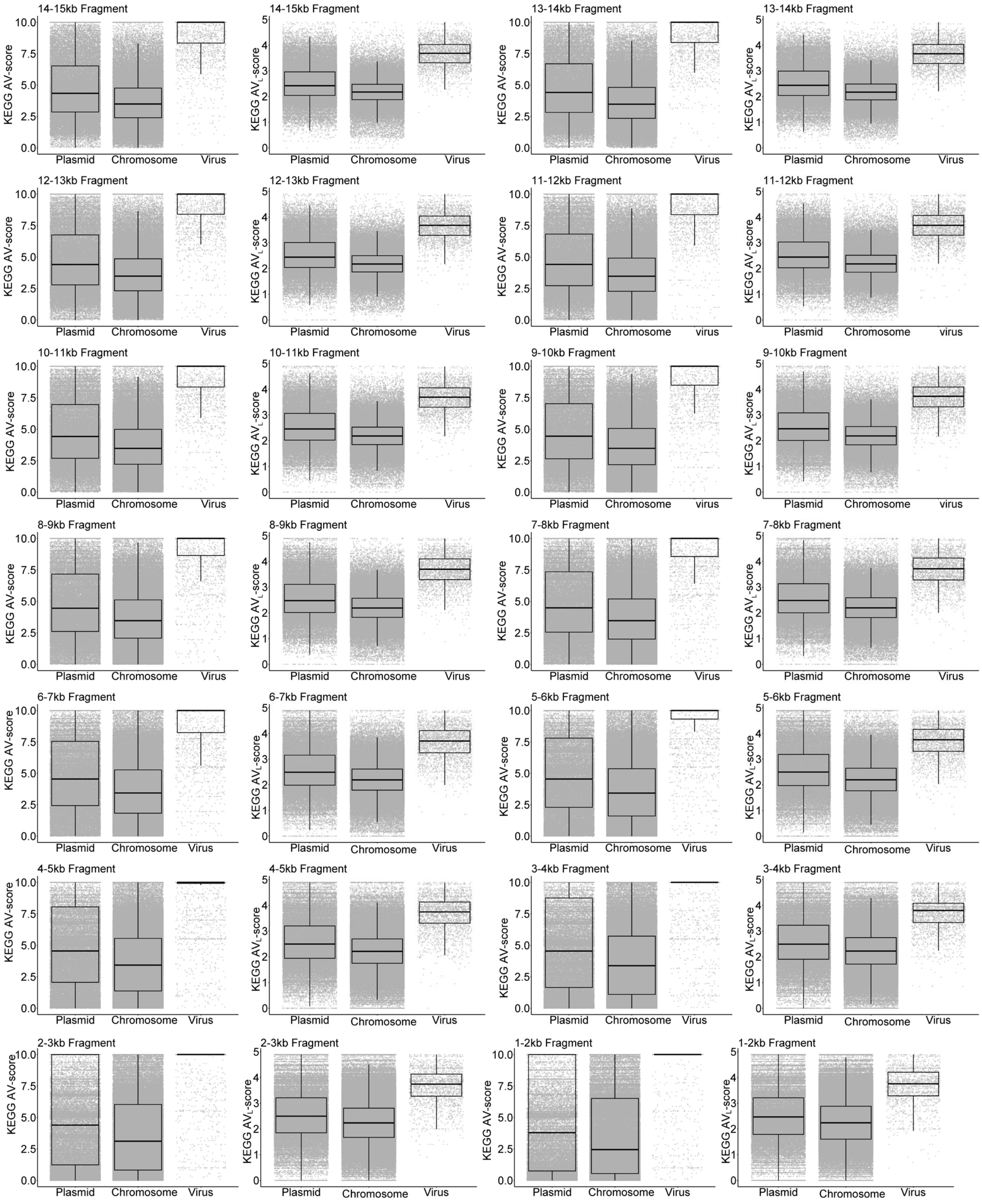

Supplementary Fig. S2 Distribution of KEGG AV-score and AV$_L$-score of split prokaryotic chromosome fragments (n = 1,975,048) and the split genome fragments of plasmids (n = 681,614) and prokaryotic viruses (n = 48,880). The horizontal line that splits the box is the median, the

upper and lower sides of the box are upper and lower quartiles, whiskers are 1.5 times the interquartile ranges and data points beyond whiskers are considered potential outliers.

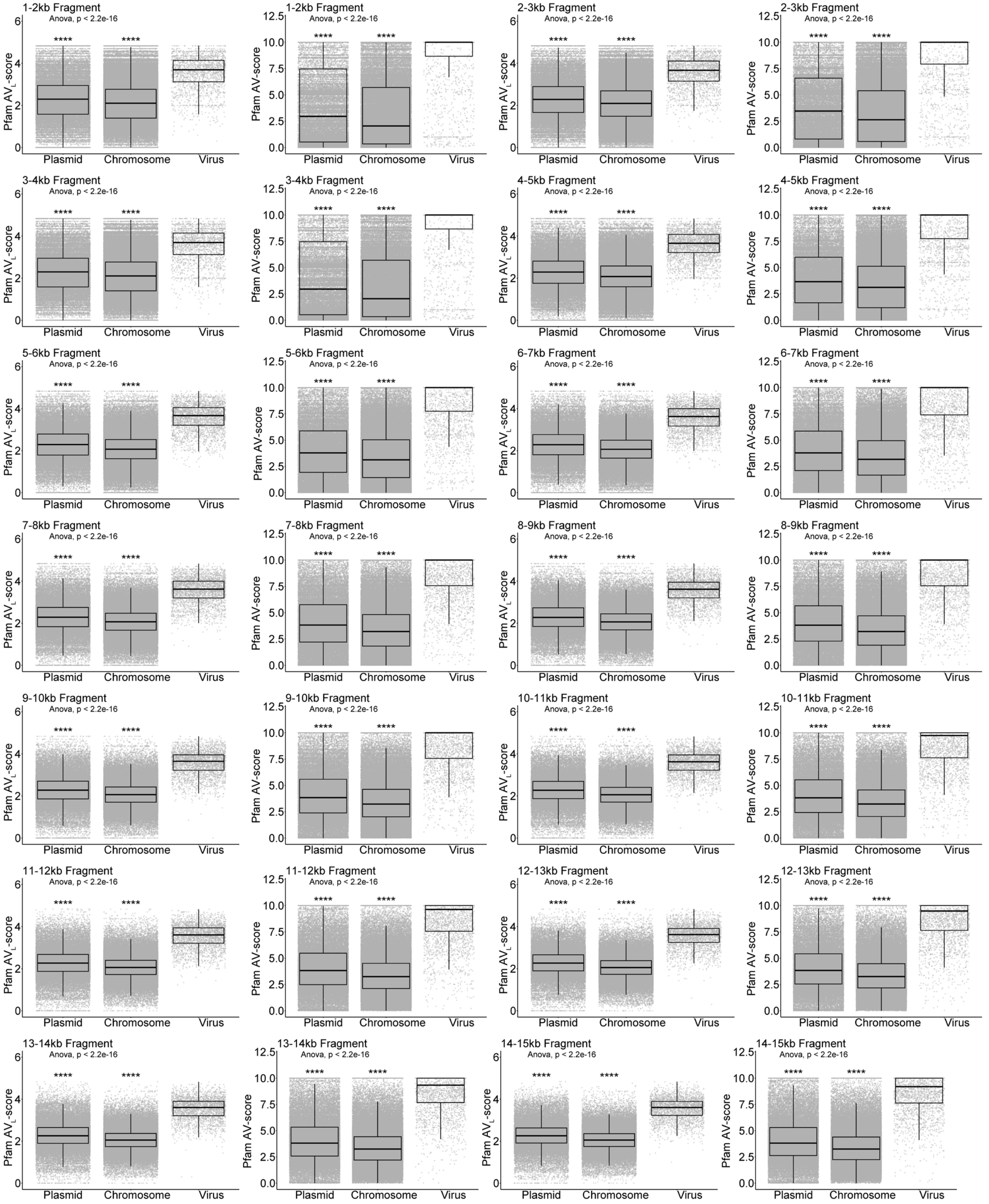

Supplementary Fig. S3 Distribution of Pfam AV-score and AV_L-score of split prokaryotic chromosome fragments (n = 1,975,048) and the split genome fragments of plasmids (n = 681,614) and prokaryotic viruses (n = 48,880). The horizontal line that splits the box is the median, the upper and lower sides of the box are upper and lower quartiles, whiskers are 1.5 times the interquartile ranges and data points beyond whiskers are considered potential outliers. An ANOVA test was used to show differences between three means are significant ($p < 2.2 \times 10^{-16}$). The asterisks (****) indicate a comparison with viruses, with a significance level of $p < 10^{-4}$.

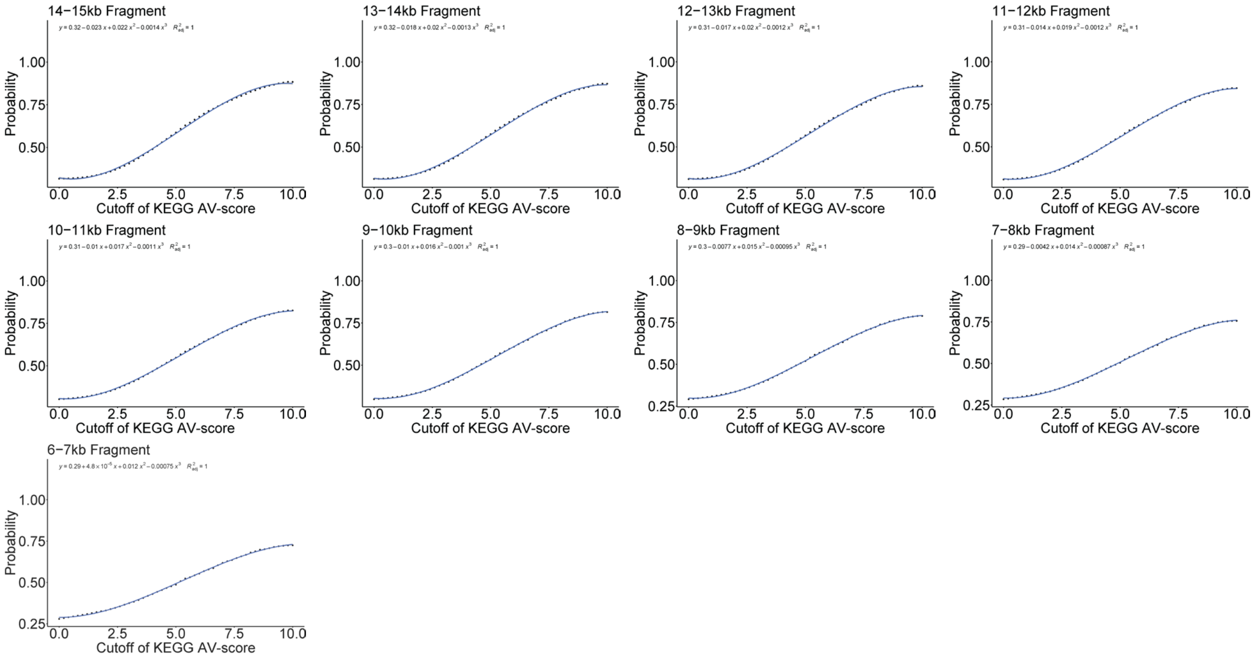

Supplementary Fig. S4 Relationship between the cutoffs (see the definition of cutoff in **Supplementary Fig. S13**) of the KEGG AV-score and AV_L-score of whole genomes used in **Supplementary Fig. S2 and S3** and the fraction of viral genomes (here was defined as probability) above certain cutoffs.

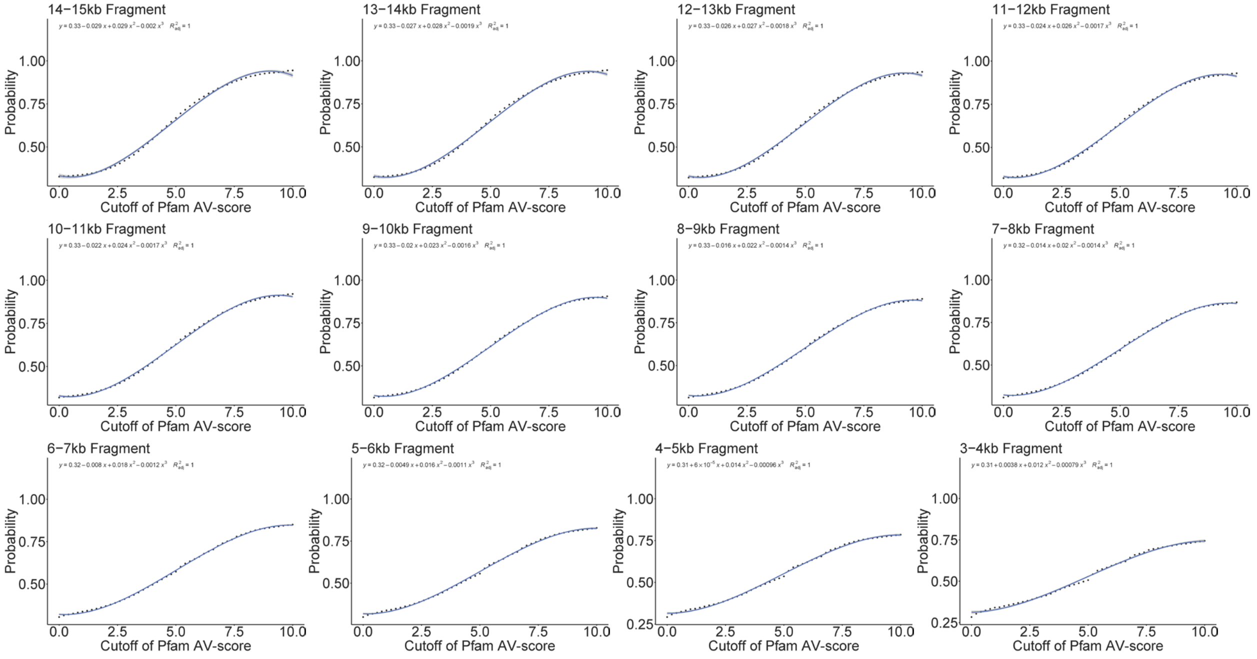

Supplementary Fig. S5 Relationship between the cutoffs (see the definition of cutoff in **Supplementary Fig. S13**) of the Pfam AV-score and AV_L-score of whole genomes used in

Supplementary Fig. S2 and S3 and the fraction of viral genomes (here was defined as probability) above certain cutoffs.

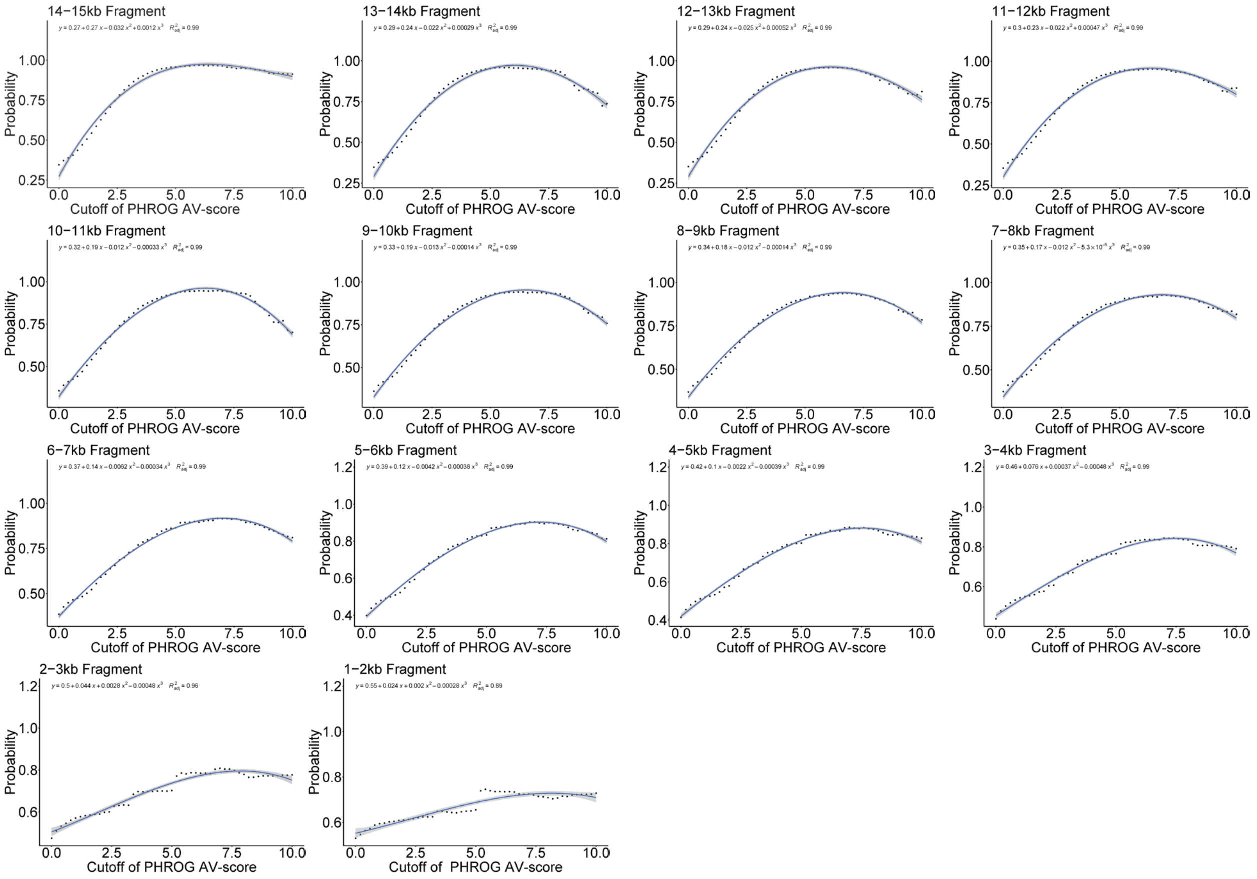

Supplementary Fig. S6 Relationship between the cutoffs (see the definition of cutoff in **Supplementary Fig. S13**) of the PHROG AV-score and AV_L-score of whole genomes used in **Supplementary Fig. S2 and S3** and the fraction of viral genomes (here was defined as probability) above certain cutoffs.

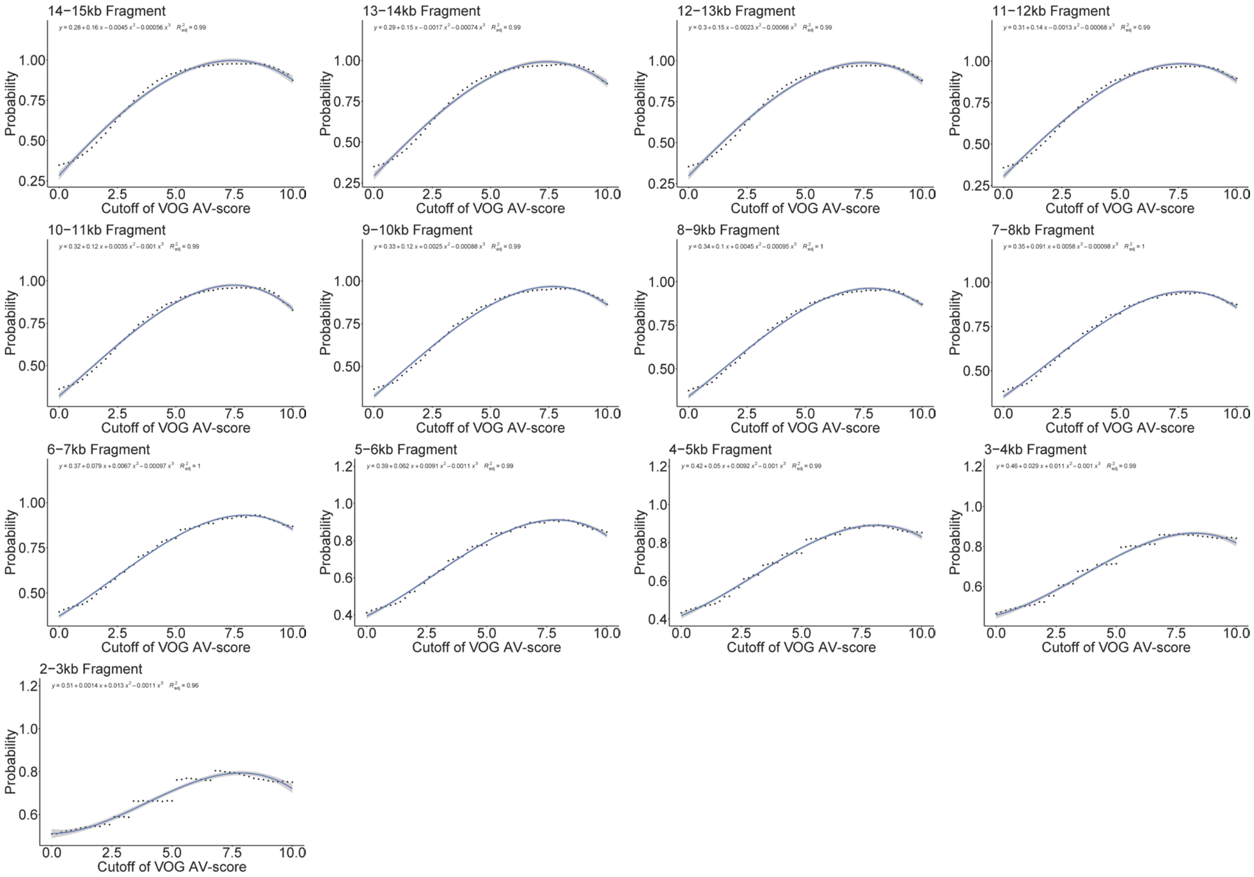

Supplementary Fig. S7 Relationship between the cutoffs of the VOG AV-score and AV_L-score of whole genomes used in **Supplementary Fig. S2 and S3** and the fraction of viral genomes (here was defined as probability) above certain cutoffs.

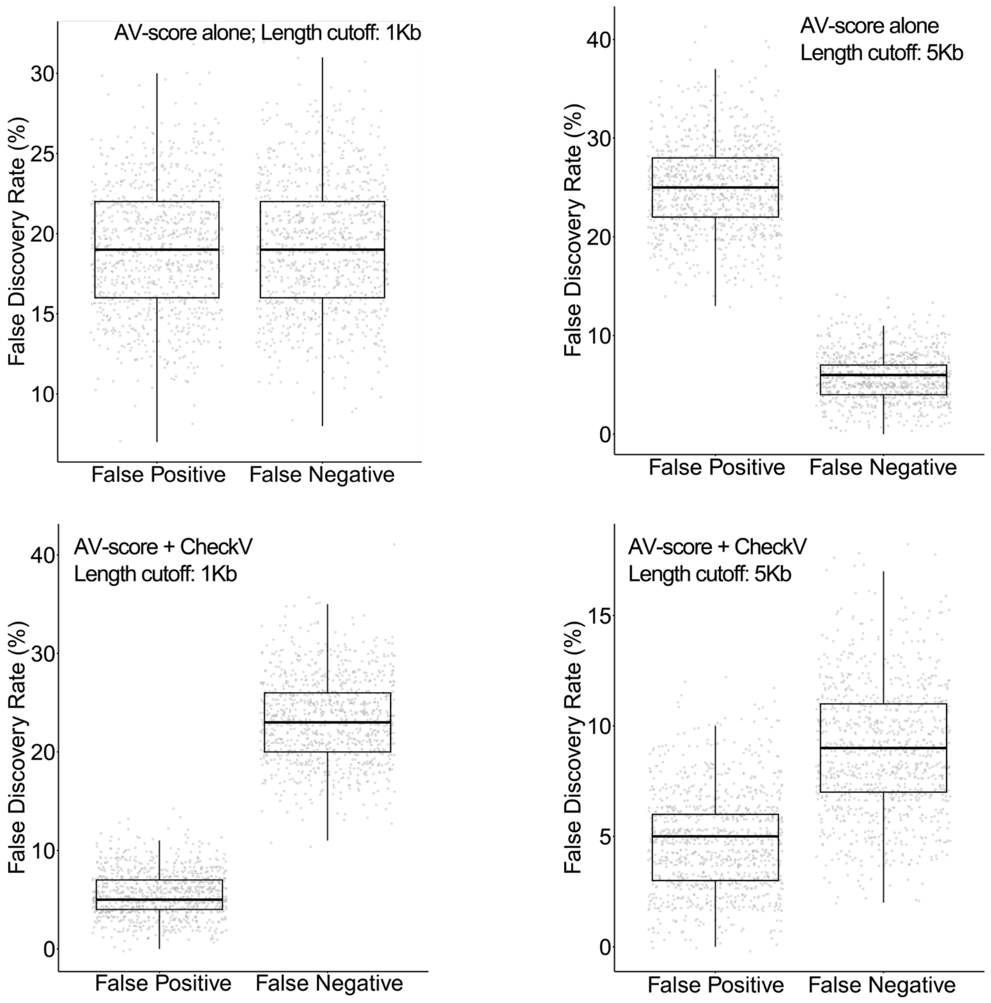

Supplementary Fig. S8 Distribution of false negative and positive rates in viral identification. False positives are defined as prokaryotic sequences that were erroneously identified as viral sequences based on the AV-score approachs (AV-score alone or combined with CheckV). False negatives are viral sequences that were not identified as viral sequences using the AV-score approachs (AV-score alone or combined with CheckV). To assess these rates, we randomly selected 100 prokaryotic and 100 viral sequences and calculated the false negative and false positive rates. This randomization and calculation were repeated 1000 times using a bootstrap resampling method. Box plot displays: median (horizontal line), upper and lower quartiles (box edges), whiskers (1.5 times the interquartile range), and potential outliers (points beyond whiskers).

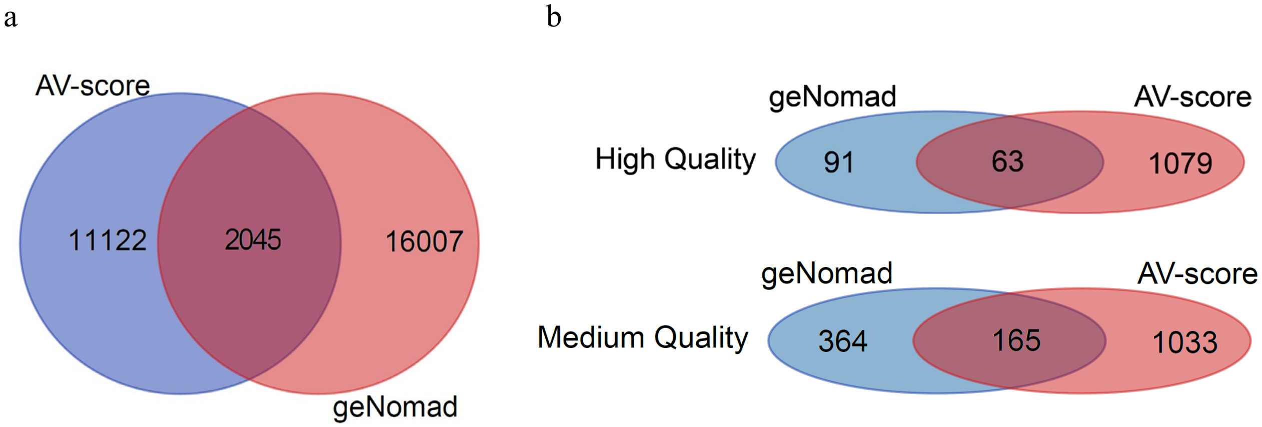

Supplementary Fig. S9 Number of low-, medium-, and high-quality viral sequences (contigs ≥ 1kb) identified using AV-scores and geNomad (a). A Venn diagram was used to display the number of sequences shared between the two approaches. For medium- and high-quality sequences, as assessed by CheckV, the overlap between the two approaches (geNomad and AV-score) was illustrated using Venn diagrams, showing the number of shared sequences identified by both methods (b).

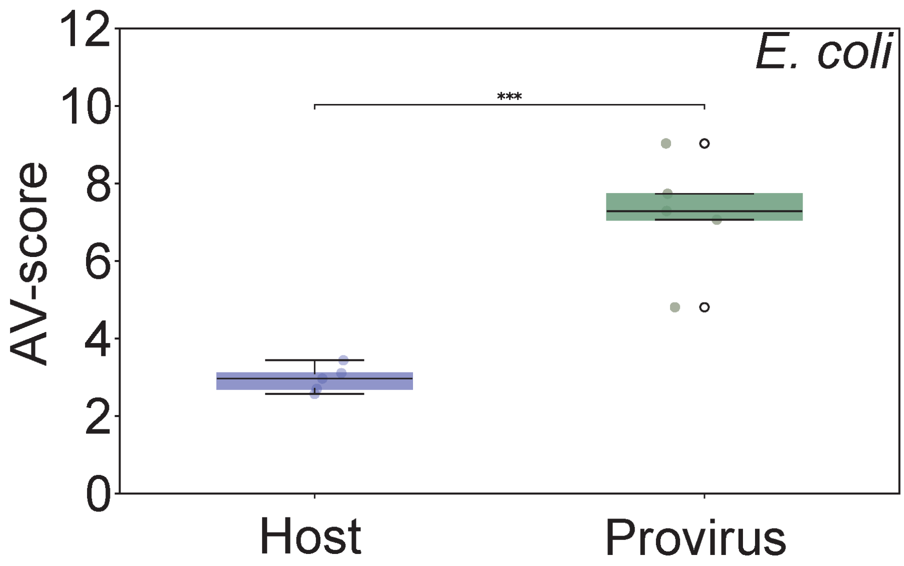

Supplementary Fig. S10 Distribution of AV-scores of Pfam, KEGG, eggNOG, VOG, and eggNOG of the *E. coli* host and its provirus genomes. The asterisks (***) indicate a significance level of $p < 10^{-3}$.

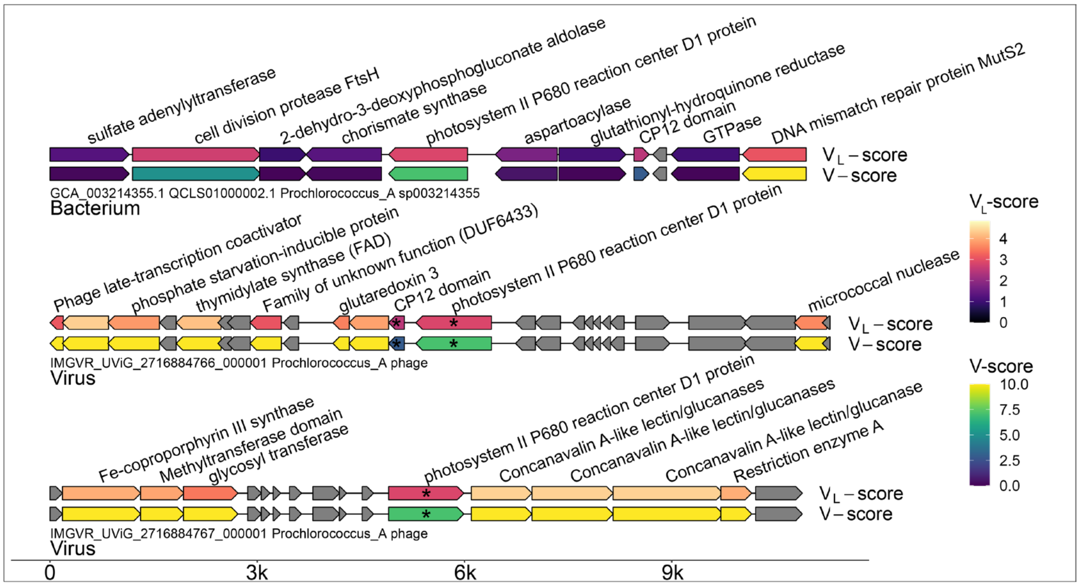

Supplementary Fig. S11 V-scores and V_L-scores reveal AMGs in viral genomes and distinguish AMGs from host-encoded metabolic genes. Genes with an asterisk (*) were predicted as AMGs using the described workflow (see Methods). We visualized the genomic context of one predicted AMG, the photosystem II P680 reaction center D1 protein (*psbA* KO K02703), in viral and host genomes. We identified one *Prochlorococcus* host genome (GenBank GCA_003214355.1) and two viral genomes (IMGVR_UViG_2716884766_000001 and IMGVR_UViG_2716884767_000001) encoding *psbA* (Supplementary Table S18) predicted by IMG/VR to be *Prochlorococcus* phages. We plotted genes within localized regions of these genomes using the R package gggenomes (version 1.0.0) using annotations, V_L-scores, and V-scores obtained as described in Methods.

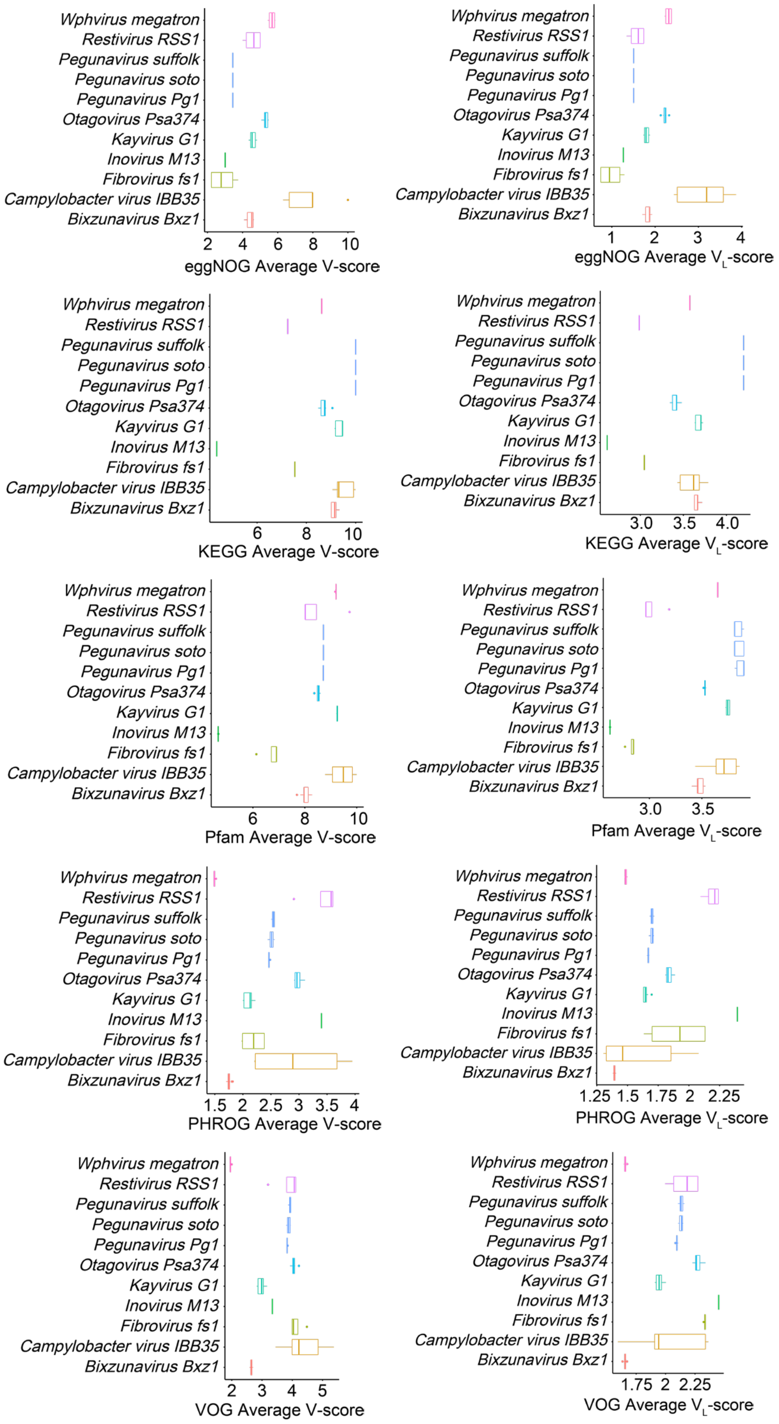

Supplementary Fig. S12 Viral population differentiation with AV-scores and AV_L-scores. Viral species include *Bixzunavirus Bxz1* (n = 13), *Campylobacter* virus IBB35 (n = 5), *Fibrovirus fs1* (n = 4), *Inovirus M13* (n = 8), *Kayvirus G1* (n = 15), *Otagovirus Psa374* (n = 7), *Pegunavirus Pg1* (n = 6), *Pegunavirus soto* (n = 5), *Pegunavirus Suffolk* (n = 6), *Restivirus RSS1* (n = 4), and *Wphvirus megatron* (n = 4). The horizontal line that splits the box is the median, the upper and lower sides of the box are upper and lower quartiles, whiskers are 1.5 times the interquartile ranges and data points beyond whiskers are considered potential outliers.

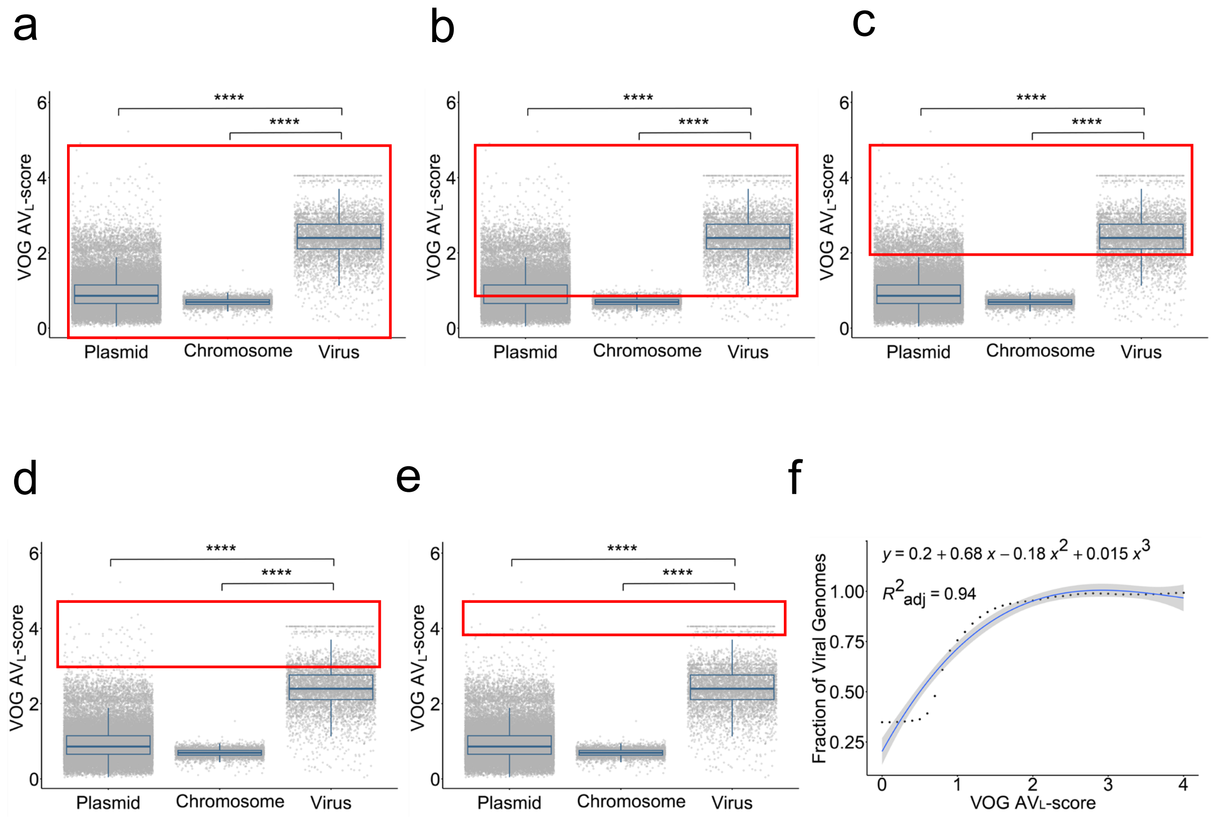

Supplementary Fig. S13 Illustration of the process used to calculate the fraction of viral genome sequences by comparing plasmids, chromosomes, and viruses. In **a–e**, each dot represents a genome sequence, while each red box indicates a sub-dataset (five examples are displayed). The red boxes emphasize variations in the ratios of viral, plasmid, and chromosomal sequences and demonstrate our methodology for calculating the fraction of viral sequences within each sub-dataset. The top edge of each red box is fixed (corresponding to an VOG AV$_L$-score of 5), while the bottom edge varies. We observed that as the bottom edge of the red boxes ascends—signifying an increase in VOG AV$_L$-scores—the fraction of viral sequences within the red box correspondingly increases. We tested more than 40 distinct VOG AV$_L$-scores, each aligning with a different position for the bottom of the red boxes. A dot plot was created to illustrate the relationship between the fraction of viral sequences and the varied VOG AV$_L$-score ranges (**Supplementary Fig. S13f**). A discernible pattern emerged, indicating that the fraction of viral sequences rises as the lowest VOG AV$_L$-scores increase, resulting in a narrower range, as reflected by the upward movement of the bottom edge of the red boxes. A polynomial regression analysis of the dot plot yielded a formula linking the fraction of viral sequences to the VOG AV$_L$-score ranges (**Supplementary Fig. S13f**). We define the fraction of viral sequences as the probability of a sequence being viral, with the VOG AV$_L$-score corresponding to the bottom edge of the red boxes serving as the cutoff for identifying viral-like sequences (these definitions also apply to AV-scores). This framework enables us to predict viral sequences based on both AV$_L$-scores and AV-

scores. For instance, if a genome sequence has an VOG AV_L-score of 2, it resides in the sub-dataset with AV_L-scores ranging from 2 to 5, where the fraction of viral sequences is 90% (as shown in **Supplementary Fig. S13c**). This signifies that the probability of this sequence being viral is 90%. In **Supplementary Fig. S13f**, the dots along the dotted line represent the observed fraction of viral proteins, while the blue lines denote the predicted values.
