# Supplementary material for: V- and VL-scores unveil viral signatures and origins of protein families
Source: Nat Commun. 2026 Apr 28;17:5828. doi: 10.1038/s41467-026-72028-0 (PMC13332234; doi:10.1038/s41467-026-72028-0)
Supplement: Supplementary file 2 — Description Of Additional Supplementary Files [file 41467_2026_72028_MOESM2_ESM.pdf]

Description of Additional supplementary files

Title: Supplementary Data 1

Description: A list of KEGG annotations along with their V-scores and V_L-scores (for hits greater than zero).

Title: Supplementary Data 2

Description: A list of Pfam annotations along with their V-scores and V_L-scores (for hits greater than zero).

Title: Supplementary Data 3

Description: A list of eggNOG annotations along with their V-scores and V_L-scores (for hits greater than zero).

Title: Supplementary Data 4

Description: A list of VOG annotations along with their V-scores and V_L-scores (for hits greater than zero).

Title: Supplementary Data 5

Description: A list of PHROG annotations along with their V-scores and V_L-scores (for hits greater than zero).

Title: Supplementary Data 6

Description: A table of the fraction of viral genomes across varying KEGG AV-score and AV_L-score cutoffs for fragmented sequences.

Title: Supplementary Data 7

Description: A table of the fraction of viral genomes across varying Pfam AV-score and AV_L-score cutoffs for fragmented sequences.

Title: Supplementary Data 8

Description: A table of the fraction of viral genomes across varying VOG AV-score and AV_L-score cutoffs for fragmented sequences.

Title: Supplementary Data 9

Description: A table of the fraction of viral genomes across varying PHROG AV-score and AV_L-score cutoffs for fragmented sequences.

Title: Supplementary Data 10

Description: A table of viral genome detection probability by sequence size.

Title: Supplementary Data 11

Description: AMG identification workflow results. Only viral and host genes with annotations in the list of Pfam/KEGG HMM profiles of experimentally verified AMGs (see Methods), or genes with annotations in the list of non-AMGs (see Methods), or genes with annotations in the list of viral hallmark genes (Table S11) are included. With V-score flank verification: at least one gene with a V-score of 10 must have been within 10 kB to the left and right flanks of a potential AMG to mark it as a predicted positive. With hallmark flank verification: at least one gene with an annotation in the list of viral hallmark genes (Table S11) must have been within 10 kB to the left and right flanks of a potential AMG to mark it as a predicted positive. Without flank verification: V-scores or hallmark annotations of genes flanking a potential AMG were not checked when predicting AMGs. Predicted positives are genes with Pfam V_L-scores < 3 and/or KEGG V_L-scores < 3, and Pfam V-scores < 10 and/or KEGG V-scores < 10, and are encoded on a contig with a mean Pfam and KEGG AV_L-score < 3, and are flanked by at least one gene with a KEGG or Pfam V-score of 10 on the left and right. True positives are genes with annotations matching experimentally verified AMGs (see Methods) encoded on known viral genomes. True negatives are genes with annotations matching to experimentally verified AMGs encoded on known host genomes or viral genes matching to a subset of non-AMGs (see Methods).

Title: Supplementary Data 12

Description: Confusion matrices of the AMG identification results presented in Supplementary Data 11.

Title: Supplementary Data 13

Description: All viral genes predicted to be auxiliary using the AMG identification workflow with/without flank verification. With flank verification: at least one gene with a V-score of 10 must have been within 10 kB to the left and right flanks of a potential AMG to mark it as a predicted positive. Without flank verification: V-scores of genes flanking a potential AMG were not checked when predicting AMGs.

Title: Supplementary Data 14

Description: VIBRANT AMG predictions for the same virus genomes present in Supplementary Data 13.

Title: Supplementary Data 15

Description: DRAM-v AMG predictions for the same virus genomes present in Supplementary Data 13.

Title: Supplementary Data 16

Description: Protein family HMM profiles of experimentally verified AMGs used to test our AMG identification method.

Title: Supplementary Data 17

Description: Protein family HMM profiles of non-AMGs used to test the AMG identification method.

Title: Supplementary Data 18

Description: A list of host and virus genomes used to test the AMG identification method. 'Host taxonomy' is the predicted host taxonomy from IMG/VR if 'Type' is 'Virus', and is the GTDB taxonomy for the genome if 'Type' is 'Host'.

Title: Supplementary Data 19

Description: GeNomad results for host genomes included in the proposed AMG identification workflow evaluation.

Title: Supplementary Data 20

Description: Viral hallmark genes in the database of KEGG and Pfam HMM profiles used for annotation. KEGG and Pfam HMM profiles were considered to represent viral hallmark genes if their annotation/description contained any of the following keywords: virion structure, capsid, portal, tail, terminase.

Title: Supplementary Data 21

Description: AV and AV_L scores for reference prokaryotic viruses used in population differentiation analysis.
