# Supplementary material for: V- and VL-scores unveil viral signatures and origins of protein families
Source: Nat Commun. 2026 Apr 28;17:5828. doi: 10.1038/s41467-026-72028-0 (PMC13332234; doi:10.1038/s41467-026-72028-0)
Supplement: Supplementary file 24 — Reporting Summary [file 41467_2026_72028_MOESM24_ESM.pdf]

nature portfolio

Peer Review File

V- and VL-Scores Unveil Viral Signatures and Origins of Protein Families

Corresponding Author: Dr Karthik Anantharaman

This file contains all reviewer reports in order by version, followed by all author rebuttals in order by version.

Version 0:

Reviewer comments:

Reviewer #1

(Remarks to the Author)
Zhou et al.'s paper, "V- and VL-Scores Uncover Viral Signatures and Origins of Protein Families," introduces the V-score and VL-score, metrics designed to quantify the "virus-likeness" of protein families and genomes. While these metrics are central to the study, the paper fails to clearly explain how they are calculated, which is a fundamental issue. Instead, the authors assume prior reader familiarity, creating a significant gap in comprehension. Although the methods section provides some details, it does not offer a clear explanation of how these scores are generated, particularly in the "V-score and VL-score generation" section. Without this clarity, readers cannot fully understand the study's approach or assess its findings. As a result, the manuscript requires major revision before publication.

Additionally, the paper contains some superfluous and logically inconsistent statements that undermine its clarity. For instance:

Line 47-48: "viruses are difficult to culture in the laboratory necessitating advances in computational approaches to study uncultured viruses." This statement is logically flawed. The difficulty of culturing viruses does not inherently necessitate computational approaches; rather, computational methods are one possible solution among several.

Line 49-50: "This knowledge helps unravel the complexity of life" The claim is vague and lacks justification. How does understanding viral genomes and proteins contribute to unraveling "the complexity of life"? The manuscript would benefit from a more precise and substantiated statement.

Major concerns:

Line 82-83: "We identified cutoffs of V-score = 0.01 and VL-score = 0 to define viral proteins with high certainty." How did you do this? This is a core piece of your work and you just jump right in.

Line 84-86: "Protein families associated with viral proteins constituted approximately 76.9%, 52.1%, and 38.7% of the total protein families in KEGG (20,005), Pfam (10,835), and eggNOG (135,509), respectively." What did you define as a viral protein? What were you searching? Without knowing how you calculated the scores at the core of your work, it's tough to know what you mean.

I stopped here in the main text because I needed to know how the metrics were calculated.

Line 344-346: V-score and VL-score generation section – "The V-score and VL-score for each annotation profile in the KEGG, Pfam, eggNOG, PHROG, and VOG databases was determined based on the number of significant hits (E-value ≤ 10-5) identified by hmmsearch (HMMER 3.4)58 and MMseqs2." Significant hits to what? This does not clearly explain how the scores were generated.

I can't ascertain how the scores were generated, so I can't evaluate the paper fully.

(Remarks on code availability)

The code appears to be functional, but the paper fails to clearly explain how the V- and VL-scores are calculated. This lack of clarity makes it difficult to properly evaluate the code, as it relies on these computed scores.

Reviewer #2

(Remarks to the Author)
I. Overall comments
This manuscript introduces a novel approach (V-score/VL-scrore) to identify viral-like sequences from metagenomes and genome, using protein sequences from virome and RefSeq databases (e.g., RefSeq, VOG, PHROG, IMG/VR) as references to score their likelihood to be viral. The authors then use these viral-like protein sequences to sort viral genomes/sequences based on their AV-score/AVL-score, including the identification of Auxiliary Metabolic Genes (AMGs). This concept is commendable as it aims to complement the traditional reliance on viral hallmark genes for detection, potentially offering a significant enhancement to existing viral sequence identification pipelines. I do appreciate the authors have brought overwhelming evidence to show how useful this approach would be. However, I do have a few concerns (as below) about the methodology and experiment design, and I worry that some of the obtained results might be biased or even incorrect. Overall, the manuscript is worth pursuing, but it can benefit from improvements by addressing these concerns, providing limitations/pitfalls about the method, and increasing the MS' clarity.

II. Major comments

1. Concern regarding the database.
As far as I can understand, the authors calculate the V-score/VL-score based on the number of hits to the constructed 'viral protein database' (RefSeq, VOG, PHROG, IMG/VR). Thus, the constructed 'viral protein database' is the key to the whole method and influences all following AV-score/AL-score, prophage/virus genome identification, as well as AMGs identification. I do like the concept of V-score, but I do have concerns. I feel safe to trust the V-score/VL-socre calculated from the 'real' virus database (such as RefSeq, VOG), but not from the 'predicted' virome database (such as the metagenomic contigs in IMG/VR). Despite the authors having used these "high-quality" and "medium-quality" contigs/genomes from metagenomes of IMG/VR, the chance to introduce biases can be inevitably high. Basically, a sequence with certain similarities (although it is not clear in the current version of the manuscript how the authors set this threshold) to the proteins present in the constructed 'viral protein database' would generate a hit, thereby adding up a V-score/VL-score. If there is a 'fake virus' (or non-viral genes) in the constructed 'viral protein database', then it would artificially increase the V-score/VL-score, thereby falsely assigning a sequence to be viral.

Second, I deeply worry about these so-called "AMG" genes in the viral metagenomes (such as IMG/VR), whether they are real AMGs or false AMGs, and whether they are functionally annotated or hypothetically unknown. Given there is a high similarity between AMGs and host genes and the search are based on sequences similarity search (again, not clear on which settings that the authors used when they denote a V-score hit during the usage of hmmscan and mmsseq), a host gene can easily be assigned to be viral if they have an AMG relative found in the constructed 'viral protein database'. This will inevitably oversize / over-claim the host gene to be viral. This would be one of the reasons why the manuscript/result showed such a large amount of new AMGs that have not been found before, as well as a high portion of 'viral gene' found in KEGG, EggNOT, and Pfam database.

In all, my point is that using metagenomic contigs to calculate V-score is like using 'unsure/unknown/uncertain' to generate more 'unsure/unknown/uncertain'. I know some other pipelines also use this approach, but I do want to express this concern. High-quality contigs from IMG/VR might be fine, but the medium quality, I'm not sure. I do feel safe to use proteins or protein databases of the real virus genomes as the basis for the V-score search or database construction. In this version of the manuscript/method, I do worry there is a potentially high chance for these host genes to be assigned to be viral, particularly those similar to AMGs.

2. Concern regarding AMG identification.
I appreciate that the authors have made a lot of effort to justify their AMG identification using the V-score method. However, I'm not convinced by their experimental design to proceed with this. The authors analyzed 5116 high-quality contigs from IMG/VR and found there are numerous AMGs that have not been detected before, even by other methods such as virsoter2 and vibrant. However, I do not think this is a good experimental design to come to this conclusion. Because these IMG/VR viral contigs are originally included in the 'viral protein database' for V-score calculation. So, it is not surprising to screen them out and score them with a high V-score, when using a sequence blast/align against a database (V-score viral protein database) containing these sequences.

Second, the authors seem like to claim that V-score method alone (without hallmark genes) allows the identification of AMGs. However, it required to be testified using a robust 'viral protein database' as I mentioned above.

Third, given that the proper databases have been applied, another concern that may be raised would be the intrinsic drawback of the V-score method. It may be hard to determine the genes of AMGs given that they are closely similar to host genes. The AV-score or V-score criteria (Lines 530-535) that the authors used for AMG determination are not clear to me. For example, why the KEGG AV-score value >4.8, is good enough to distinguish viral AMGs from host genes? I just miss all the reasoning about these arbitrary settings. In addition, the current KEGG/Pfam/eggNOT V-score is likely biased if it's not derived from a robust 'viral protein database' as I mentioned above.

3. V-score/VL-score/AV-score/AVL-score cutoff to determine whether a sequence is viral or non-viral.

It’s interesting to see the authors used a ‘community approach’ to determine the cutoff of V-score/VL-score/AV-score/AVL-score to be viral or non-viral. I find it hard to understand maths/statistics without a clear explanation (in the manuscript) of what it means. For example, a protein sequence with a V-score above the cutoff for the probability of 70%, this protein was assigned as viral. So, If I understand well, the cutoff is determined by detecting 70% of protein being viral at that V-score for a given dataset.

First, I found that a protein’s viral origin determined by probability seems ‘crazy’. There is still a 30% chance that this V-score does not support this protein sequence being viral, based on the analysis of the specific dataset. Would this introduce false positives? Even with a 90% probability for the ‘most likely’, there is still a 10% chance this sequence is non-viral.

Moreover, the reliability of these probabilistic cutoffs could be greatly influenced by the variability in dataset characteristics, such as dataset size and community composition differences between virus, plasmid, and host genomes. For example, the same protein could yield different V-score categorizations when analyzed within the context of a smaller, simpler dataset compared to a larger, more complex dataset. Such discrepancies can raise critical questions about the consistency and universality of the cutoff values. Do the authors take these potential drawbacks into account? Similar concerns are raised for the AV-score cutoff in the determination of a contig to be viral or non-viral.

4. V-score / VL-score normalization
Given that the V-score is calculated based on hits to a 'viral protein database' and considering that databases such as RefSeq are regularly updated, the long-term reliability of V-score / VL-score may be affected. To maintain consistency and comparability over time, I feel like the V-score needed to be normalized by the total number of proteins in the constructed database. This normalization would help counteract the effects of database updates on the scores and their cutoffs, thereby enhancing the reliability of these metrics for determining whether a sequence is viral or non-viral, even in future when the database is updated.

5. V-score/AV-score in viral species/strain differentiation and metagenome binning
It’s interesting to see the V-score/AV-score method can be used for metagenome binning and viral species/strain differentiation. I’m just not convinced that this conclusion can be drawn from the analysis of only 11 viral species, given there are hundreds and thousands of viral species/strains in the database. Do these 11 viral species speak the ground truth for all viruses or any type of viruses (dsDNA virus, RNA virus, ssDNA virus, giant virus, virophage, etc) in the database? Nevertheless, I believe that analysis of the full set of databases is valuable to get the point the authors want to make.

6. Discuss the limitation, drawback and pitfall
Given this manuscript introduces innovative methodologies for identifying viral-like proteins/genomes, it is crucial to add a section in the Discussion to addresses potential biases and pitfalls inherent in these methods. However, in current version of the manuscript, I haven’t seen these words. I would be appreciated if the authors add a section in the discussion section to discuss the potential limitations or biases that might affect the interpretation of these scores, such as the database dependency of results.

III. Specific Comments

Author’s affiliations
• Lines 4-5: Perhaps it is not my position to comment the author’s affiliation, yet it may look ‘odd’ to some to see an NSA/NIH funding to support a work in a ‘foreign’ university (1st affiliation). I assume that the 1st author conducted his/her/their postdoc for this work in University of Wisconsin–Madison, and now his/her/their moved to Tongji University in China for a faculty job. If this is the case, it would be more appropriate for the University of Wisconsin–Madison to be listed as the first affiliation, with Tongji University noted as the ‘current address’ or second affiliation. I raise this issue because in China, the first-listed affiliation of a paper significantly impacts staff promotion, funding acquisition, and all other formal evaluations. I understand that the PI would like to help the 1st author to be fast-tracked in his current university. But, from a point view of any other colleagues in China, this is unfair practice to them. I hope the authors could reconsider the order of affiliations to accurately reflect the contribution and geographic location of the work.

Abstract
• Line 30: do you mean virus-host co-evolution? Please be specific.

• Line 36: up to 77% of protein family in KEGG/eggNOT/Pfam are viral? Please double-check. Please see my concern related to the method as listed above. I worry that this high portion is associated with methodology drawback, in particularly the bias associated with V-score database (viral protein database that includes metagenomic contigs), as well as the indigenous drawback of this V-score method that might falsely assign host gene to be viral.

• Line 38: “precise”: I’m not sure this word is still legitimated given the bias that I mentioned above.

• Line 40: “17x”. I’m not sure this number given the methodology bias and experimental design issue as I mentioned above. Certainly, AMGs or AVGs needs to be re-analyzed to be sure that is true. Please see my detailed comment below in the result and method section.

Introduction

• Line 46: I guess it means virus-host co-evolution. Please be specific.

Results

• Lines 77-92: It's surprising to see such high portion of genes in KEGG, Pfam and eggNOG database to be viral. My guess is the bias associated with V-Score method that falsely identify the host protein/genes (which is similar to AMGs) to be viral. Second, compared to RefSeq and VOG database, the IMG/VR database may not be an ideal database for this type of analysis. Because IMG/VR contain the metagenomic contigs that comprise anything, although the authors have chosen the medium/high-quality contig for database construction. Thus, these sequences (or specifically the protein it encodes) would lead to a bias as the AMGs does. I feel like it's safe to use only the 100% sure viral genomes for the database construction, and then next to predict the potential viral profiles in KEGG, Pfam and eggnot.

• Lines 88-92: I would be moderate about the claims until a proper robust analysis was done and 100% sure that they are viral. At this point, I'm afraid I am not convinced by current data.

• Lines 112-113: Why VL-score>4 is considered as viral origin? And why VL-score<2.2 is considered non-viral? Without any clear explanation in the text in the manuscript, it's hard for anyone to understand this. Or, do I miss anything?

• Lines 164-166: Without clearly showing parameter settings for other pipelines in the method (e.g., VirSorter2), it's hard to be persuaded that AV-Score "surpassed" others. Given that AV-Score may "surpassed" other pipelines, it's important for readers to know the potential bias/pitfall about this 'surpass'.

• Lines 208: Are these 17 AMGs in Table S16? It would be great to list them name or point to the table location.

• Lines 217-218: Why this standard hinders AMG recall for non-proviruses?

• Lines 221-224: Given the bias/concern related to the V-score method, as mentioned above, I am not convinced that the V-score alone can robustly identify AMG without the help of the hallmark gene. It does not mean that it can't. It is just that the current manuscript has not yet convinced me.

• Lines 226-247: Here, I think there is some sort of experimental design problem. I do not think the claim of detection of numerous unknown AMGs in these 5116 high-quality contigs (from IMG/VR) is valid. Note that these IMG/VR viral contigs are originally included in the 'viral protein database' for V-score calculation. So, it is not surprising to screen them out and score them with a high V-score when using a sequence blast/align against a database containing these sequences.

• Lines 248-261: The same problem here. If your V-score database already contains these sequences, it is not a surprise to see the V-score method out-compete other methods in revealing AMGs.

• Lines 264-293: Why only 11 species? There are hundreds and thousands of species/strains in the database. It's hard to believe, using only 11 viral species, that the authors claim that the V-score method allows species- or strain-level differentiation and facilitates binning among bacteria. Definitely, it requires a broader analysis and data to support this claim. Nevertheless, there are all kinds of viruses: dsDNA virus, RNA virus, ssDNA virus, giant virus, virophage, etc.

Discussion

• Line 294-320: The discussion is so brief here and only focuses on the cherries-picked good results without acknowledging their bias/pitfall/drawback. Given many concerns, biases, pitfalls, and drawbacks about the method, experimental design, and results, I suggest the authors add a well-described section to tell the readers about the drawbacks of their methods and the bias/pitfalls in the results. However, I did not see any words here in this section in the current manuscript.

Methods

• Lines 324-333: The V-score method is based on the occurrence incidents of sequences that can be hit in the constructed viral protein database. Thus, how confident this database is a key to the whole methodology and its produced results. I'm a bit of concerning the use of viral metagenomic sequences/contigs/genomes in the database. It would bias the whole downstream analysis, although the authors used the "medium-quality" and "high-quality" contigs from IMG/VR. As I mentioned above, this uses "predicted" viral contigs to predict more 'viral' contigs, which basically adds another layer of uncertainty. Instead, I feel that using the protein from pure viruses is a safer way to do.

• Lines 346: It's unclear for the parameters of hmmsearch and MMseqs2 for V-score hits search. In particular, the sequence similarity threshold that the authors choose to consider is a valid hit. This is crucial for the whole process, which is the place that might introduce the bias that hosts genes to be considered as viral, which is a potential bias that I have highlighted above.

• Lines 356-373: I appreciated the efforts that the authors made to produce provirus sequences from prokaryotic genomes. Given such a high diversity of virus or viral sequences in prokaryotic genomes and a high diversity of prokaryotes, I do not think one chromosome per family is sufficient to achieve the goal of using the V-score to resolve all proviruses and tell the

ground truth. Why not use the full set of GTDB databases? Certainly, the more data introduced, the higher confidence levels of the V-score cutoff will be found. Otherwise, the authors might be biased without specifying the drawback, and they may also overclaim that it can achieve species-levels or strain-levels differencing for proviruses. I suggest the authors consider analyses of the whole database, which would provide relatively robust results/conclusions.

• Lines 366: Parameters for VirSorter2 and VIBRANT are required, which allows the readers to have a sense of how well/bad the viral contigs you are talking about or compare with.

• Lines 377-379: Need to specify how chromosomes are randomly split. Which software and what parameters, etc.

• Lines 383: Again, MMseqs2 settings? Particularly, the sequences similarity cutoff, etc.

• Lines 398-426: It's interesting to see the authors used a 'community approach' to determine the cutoff of V-score/VL-score/AV-score/AVL-score to be viral or non-viral. I find it hard to understand maths or statistics. I hope the authors can help the readers understand the reasoning why that 70% probability is good enough to assign a sequence as viral. Maybe I have missed something somewhere. It is just not clear to me.

• Lines 420-421: How was polynomials regression analysis conducted? Ref, software, and etc? I'm also curious about why 70% probability is chosen to be "likely" viral.

• Lines 475-562: I do appreciate the effort that the authors have input for AMG identification and use the 17 AMGs as a benchmark. Given the intrinsic bias this V-score method has, I worry that the V-score would falsely consider host genes as viral, thereby over-introducing the AMGs as the results showed. This is an intrinsic bias because of the host-derived nature of AMG genes. Moreover, the use of metagenomic contigs (e.g., medium quality contigs from IMG/VR) in the original 'viral protein database' for V-score calculation would exacerbate the bias. I'm just not convinced that using the V-score alone allows the identification of AMGs and differentiates them from host genes. It looks more reasonable to me that one can combine V-score and hallmarks genes to ascertain the AMGs.

• Lines 530-535: It's hard to understand why these AV-socre or V-score criteria are used for AMG determination? Any reasons…

• Lines 541: 'assembly' is a confusing word.

• Lines 544: 'excluding': do you mean including?

• Lines 563-575: Again. I think this is great to evaluate whether V-score allows differentiating viruses at species/strain levels. I worry that the authors only 'cherry-picking' 11 species to make this claim, which is not sufficiently strong. Why not to consider using the whole database that covers all species for analysis? There are hundreds and thousands of viral species/strains in the database.

Figures & Tables

• Fig1d: in the legend, it would be helpful to specify the "probability = "fraction of viral proteins". Do I understand correctly?

• Fig4d: It's hard to tell what 'specificity,' 'sensitivity,' 'precision,' 'accuracy,' and 'false discovery rate' mean and how each was calculated. I can not find this information either in the legend or manuscript.

(Remarks on code availability)

Version 1:

Reviewer comments:

Reviewer #2

(Remarks to the Author)

(Remarks on code availability)

Version 2:

Reviewer comments:

Reviewer #3

(Remarks to the Author)

The current manuscript presents a metric that can be computed on protein sequences, which is claimed to be versatile for many application for viral metagenomics: identification of viral contigs, identification of AMG, improvement of genome binning. These applications are indeed hot topics for the scientific community, and filling these technical gaps is of great interest.

Overall, I think the current manuscript is overstating the claims. Many results would need further important checks in order to be trusted. Although the general idea of measuring the “viralness” of proteins is interesting (although not novel, see [Kristensen et al. 13], a paper the authors should explicitly refer to), it is implemented here in a naïve way with strong biases that should not be ignored, and which should (and can) be mitigated or at least be quantified to prove that they can be neglected.
So I suggest to:
1. back the claims with stronger and unbiased results.
2. restrict the scope of V_L / AV_L scores to fewer applications where it shows incontestable superiority (identification of viral contigs may be a good lead). Currently, the paper consists in several claims of improvement without proper evaluation.

General issues:

- There are strong biases in databases the authors base their study: on the viral side, there is for instance a higher representation of the Caudoviricetes in databases (just as an example, a quick search on RefSeq virus gives 91,698 genomes compared to 10,434 for Malgrandaviricetes). As acknowledge in the discussion (L362-370, but not addressed in the paper), this will bias the “viralness” (as measured by the Vscores) toward high values for proteins belonging to this class of viruses, and on the contrary profiles matcing proteins present in less represented viruses will appear as little viral. In my opinion, it is crucial to correct these scores by the abundance of the viral family in the databases. In [Perez-Bucio et al. 25] the author detail a scheme allowing not directly to average over all hits, but average inside so-called “clades”, and then average over all viral clades. This removes the representation bias. This remarks may apply in particular to the claim in the discussion about the V-scores of picornavirus capsid versus Hepatitis C virus capsid (L342-345).
As the authors answer comments of reviewer 2, using environmental data mitigates the issue of culture-related biases. Nevertheless, difference relative abundance of clades still play a role here, and using raw environmental data is prone to cellular contamination.
From [Perez-Bucio et al. 25], the authors can check that high-confidence IMG/VR viral proteins, are composed of many proteins that are likely to belong to cellular contigs.
For more robustness, the authors either should consider using curated viral database or should at least check for proteins consistently found in (dereplicated) viral environmental genomes/contigs.

Major issues:

- Assessment of protein families for virus-like proteins (L82+): the author evaluate the performance of their score in terms of recall, without considering precision. Searching their profiles against databases of prophage-stripped cellular genomes may appear as a useful proxy for precision.

- Assessment of protein families for virus-like proteins (L82+): The fact that a single hit of a protein family against the built reference database of viral sequences (encompassing VOG, IMG/VR, etc.) is a sign of “association with viral proteins with high certainity” is a bold statement with little grounding. Indeed, the reference database is highly prone to contamination (e.g. plasmids, erroneous delineation of prophages encompassing bacterial genes in the flanking region, etc.), and in particular if single hits against it are considered. Also, some genes – in particular for environmental genomes – can wobble in a phage genome without persisting over generations, and is not a sign of being part of the viral world. The authors should define more clearly what they mean by “associated with viruses”: as I understand it, it means here that somewhere in the (sequenced) world a gene has been found in a viral genome (if not due to contamination) – and, to me, this definition does not bring anything relevant. Under this definition of association, I don’t see the point of “substantially increase the number of proteins […] associated with viruses” L117-L122. See also previous general comment about contamination in the reference database.

- L133: The authors claim that one can leverage V-Scores to find viral hallmark genes. High V-Scores mean that the recall of such a gene when looking for a viral protein is high, but to me, the definition of a hallmark gene is on the contrary a gene that have a strong precision (and of course, given a high precision, the higher the recall the better). So looking for homologs in the cellular genomes (bacteria, eukaryotes, archea) is mandatory to define a gene as hallmark.

- The Fig 1e is very interesting since it tells a lot on how to interpret a V(L)-score. I think representing it as a precision-recall curve (with label 1=viral genome, and 0=plasmid or chromosome) should help the reader to have a better interpretation of what means to have a V_L-score of 3.5 for instance. Here again, dereplication / balance for highly represented genomes should be done beforehand.

- The V_L scores reported in table S16 and S17 show that there is a large gap between the AMG and non-AMG selected

profiles. It is therefore expected that the precision is very high for differenciating between these two categories. To me, the only information that the "pipeline" is bringing, is the ability to differentiate a host scaffold from a viral one. The subsequent separation between AMG/non-AMG is trivial in this benchmark. An interesting benchmark would be to select a set of non-AMG genes that have a low V_L score, and in this case it seems obvious that the current method will fail.

- The actual claim about improvement of viral genome binning using AV_L scores is currently an overstatement since the authors, as I understood, made use of the AV_L score to bin their genomes, making the benchmark circular. Simulations of viral contigs (by splitting known genomes for instance) can be done to support quantitatively this claim: up to what size two contigs can be binned confidently together based on AV_L scores. Plus, in order to make this claim for real environmental data, binning should be carried out with different binning tools (COMEBin, metaBAT, etc.) and independently with a clustering using AV_L scores, and compare the binning results based on standard metrics (e.g. using CheckV).

Minor:
- Lacks of clear definition of the V- and VL- scores. In the text it says it is "based on the number of significant hits […] identified by hmmsearch and MMSeqs2 […] divided by 100". By "based on" do the authors mean "equals"? How do you handle the combination of the hits found by the two tools? This leaves the reader with a fuzzy view on this quantity crucial for the paper. I saw afterwards that the definition is provided as a small insert in Fig1. I suggest to make a proper definition clearly visible in the main text.
- I don't see the point of providing a score and its logarithm. Why not one of the two? It over-complicates the message in my opinion as the reader feels that independent information is provided by the scores.
- check for consitency innotations: VL vs V_L (e.g. L433 vs L417)
- There a confusion in the machine learning vocabulary in section 662+, which makes this hard to follow for the reader. Indeed a "true positive" is a **prediction** that turns out to be correctly predicted as a positive instance, whereas in the first paragraph of this section the authors employ this term to mean "we labeled" a specific gene as positive ; which is very confusing.
- What do the authors mean by L673 "were removed before we predicted the positives and negatives"? How this is exactly done? I wonder if the authors used the fround thruth of the genome label to so, or if they used the prediction of "host" genome leveraging the threshold on the AL_L score? If the ground truth is used here, this comes with an unfair bias on the reported specificity.
- Although I understand that the sensitivity is much reduced using a filter on viral hallmarks genes, I don't understand why the FDR is negatively impacted compared to the case where no post filtration os applied -- as shown on figure 4d? Shouldn't it be the other way around?
- No reference is given to figure described in paragraph L701+.
- Fig6: No a. and b. on the figure can be found. I guess a. andb. Refers to repsectively left and right pannels.

References:
[Perez-Bucio et al. 25] Perez-Bucio, R. ; Enault, F.; Galiez, C. EnVhogDB: an extended view of the viral protein families on Earth through a vast collection of HMM profiles. Peer Community Journal, Volume 5 (2025), article no. e100. https://doi.org/10.24072/pcjournal.627

(Remarks on code availability)

Version 3:

Reviewer comments:

Reviewer #3

(Remarks to the Author)
I would like sincerely to thank the authors for their effort in answering my questions. Overall I am satisfied with the answers and the current status of manuscript, which provides much more quantitative information (precision-recall curves for instance) and more reasonable claims.

I just would have a (last) request regarding the newly developed C-Score. As I understand, the authors average the clade-specific C-scores over all viral clades. By doing so, it washes out a potential signal. Indeed, many viral clades may be evolutionary completely independent and it does not make sense to expect a protein family to be present in all clades. In my opinion, such a C-Score should be only averaged over the clades containing at least one hit. Adding pseudo counts to correct for small protein families -- say, appearing in only one small clade -- may also be a good idea.
Thereby the 2 first points raised by the authors concerning their C-score is not fully relevant with the comment above.

Usability:
The tabulated data is correctly provided on the website, but I would suggest to:
- make a proper Zenodo (or similar) entry with this database
- add a proper link or a copy (if allowed) to the profiles/sequences mentioned in the database (with consistent sequence database version **and** matching identifier) so that the final users can quickly run a search and consolidate the results with the V-Scores provided in the table.
- provide simple script taking out the results of a standard profile search tool (Hmmer, MMSeqs for instance) and make the merge with the tabulated data. I agree that this is not a lot of work, but it can make the current work here much more used as

soon as there is such an example provided.

Minor:
- the y axis on figure 2 are not on the same scale that leads to misleading intuitions. I advise to use the same y-axis ranges to be able to compare more clearly the performance of each of the databases.

(Remarks on code availability)

Open Access This Peer Review File is licensed under a Creative Commons Attribution 4.0 International License, which permits use, sharing, adaptation, distribution and reproduction in any medium or format, as long as you give appropriate credit to the original author(s) and the source, provide a link to the Creative Commons license, and indicate if changes were made.
In cases where reviewers are anonymous, credit should be given to 'Anonymous Referee' and the source.
The images or other third party material in this Peer Review File are included in the article's Creative Commons license, unless indicated otherwise in a credit line to the material. If material is not included in the article's Creative Commons license and your intended use is not permitted by statutory regulation or exceeds the permitted use, you will need to obtain permission directly from the copyright holder.
To view a copy of this license, visit https://creativecommons.org/licenses/by/4.0/

POINT BY POINT RESPONSES TO REVIEWER COMMENTS

All line numbers in this response refer to the clean version of the manuscript.

REVIEWER COMMENTS

Reviewer #1 (Remarks to the Author):

Zhou et al.'s paper, "V- and VL-Scores Uncover Viral Signatures and Origins of Protein Families," introduces the V-score and VL-score, metrics designed to quantify the "virus-likeness" of protein families and genomes. While these metrics are central to the study, the paper fails to clearly explain how they are calculated, which is a fundamental issue. Instead, the authors assume prior reader familiarity, creating a significant gap in comprehension. Although the methods section provides some details, it does not offer a clear explanation of how these scores are generated, particularly in the "V-score and VL-score generation" section. Without this clarity, readers cannot fully understand the study's approach or assess its findings. As a result, the manuscript requires major revision before publication.

Response: Thank you for pointing out the lack of clarity in our explanation of the V-score and V_L-score metrics. We acknowledge that a clear understanding of how these scores are calculated is fundamental to comprehending our study and assessing its findings.

We have now significantly revised the manuscript to provide more detailed and accessible explanations of the V-score and V_L-score generation process. We have included additional clarifications to ensure that readers can readily grasp the underlying calculations and logic behind these metrics.

Additionally, the paper contains some superfluous and logically inconsistent statements that undermine its clarity. For instance:

Response: We have carefully reviewed the text and revised statements throughout to enhance clarity and logical consistency.

Line 47-48: "viruses are difficult to culture in the laboratory necessitating advances in computational approaches to study uncultured viruses." This statement is logically flawed. The difficulty of culturing viruses does not inherently necessitate computational approaches; rather, computational methods are one possible solution among several.

Response: The statements have been revised for improved logical accuracy (lines 47–49).

Line 49-50: "This knowledge helps unravel the complexity of life" The claim is vague and lacks justification. How does understanding viral genomes and proteins contribute to unraveling "the complexity of life"? The manuscript would benefit from a more precise and substantiated statement.

Response: We have removed the "the complexity of life".

Major concerns:

Line 82-83: "We identified cutoffs of V-score = 0.01 and VL-score = 0 to define viral proteins with high certainty." How did you do this? This is a core piece of your work and you just jump right in.

Response: A V-score of 0.01 or a V_L-score of 0 indicates that a protein family had a single match in the constructed database of 18,435,589 viral proteins. If a protein family from KEGG, Pfam, or EggNOG has at least one match, we consider the protein family to contain viral proteins and to be associated with viruses. The corresponding text has been revised accordingly (lines 110–112).

Line 84-86: "Protein families associated with viral proteins constituted approximately 76.9%, 52.1%, and 38.7% of the total protein families in KEGG (20,005), Pfam (10,835), and eggNOG (135,509), respectively." What did you define as a viral protein? What were you searching? Without knowing how you calculated the scores at the core of your work, it's tough to know what you mean.

Response: To establish links between viruses and protein families within public annotation databases, we initially generated a non-redundant set of 18,435,589 protein sequences from various viruses (see Methods for details on sequence generation). Subsequently, we searched against these 18,435,589 viral proteins to construct associations between viruses and protein families from KEGG, Pfam, and EggNOG. If a protein family had at least one match to these viral proteins, we considered it to contain viral proteins and to be associated with viruses.

Consequently, protein families associated with viral proteins constituted approximately 76.9%, 52.1%, and 38.7% of the total protein families in KEGG (20,005), Pfam (10,835), and eggNOG (135,509), respectively. These percentages highlight the proportion of protein families in each database that have identified viral protein matches, indicating the extent of viral protein representation within each database's collection of protein families.

To clarify our statements, the corresponding text has been revised accordingly (lines 82–115).

I stopped here in the main text because I needed to know how the metrics were calculated.

Response: The text has been substantially revised to improve clarity throughout.

Line 344-346: V-score and VL-score generation section – "The V-score and VL-score for each annotation profile in the KEGG, Pfam, eggNOG, PHROG, and VOG databases was determined based on the number of significant hits (E-value ≤ 10-5) identified by hmmsearch (HMMER 3.4)58 and MMseqs2." Significant hits to what? This does not clearly explain how the scores were generated.

I can't ascertain how the scores were generated, so I can't evaluate the paper fully.

Response: The V-score and V_L-score for each annotation profile in the KEGG, Pfam, eggNOG, PHROG, and VOG databases was determined based on the number of significant hits to the generated 18,435,589 protein sequences. For V-score, the resulting number was divided by 100, with a maximum limit set at 10 after division (V-score = [No. of hits]/100; Max=10). For V_L-score, the resulting number was scaled down using the common logarithm (base 10) without a maximum limit (V_L-score = Log10[No. of hits]). When annotations contain viral keywords, the V-score is set to 1 and the V_L-score is set to 2 if the original V-score is less than 1 and the original V_L-score is less than 2. To clarify the score generation process, we have revised the text in Lines 416–456 and Figure 1a. Additionally, we included an example using a KEGG protein family to demonstrate the number of hits and the resulting V-score in Lines 103–107. These revisions provide a more detailed explanation of how the V-scores and V_L-scores were calculated and adjusted based on viral keyword annotations.

Reviewer #1 (Remarks on code availability):

The code appears to be functional, but the paper fails to clearly explain how the V- and VL-scores are calculated. This lack of clarity makes it difficult to properly evaluate the code, as it relies on these computed scores.

Response: We appreciate the feedback. To enhance clarity regarding the score generation methodology, we have updated the manuscript text such as in Lines 416–456, as well as Figure 1a. These modifications offer a more comprehensive description of the V-score and V_L-score calculations.

Reviewer #2 (Remarks to the Author):

I. Overall comments

This manuscript introduces a novel approach (V-score/VL-scrore) to identify viral-like sequences from metagenomes and genome, using protein sequences from virome and RefSeq databases (e.g., RefSeq, VOG, PHROG, IMG/VR) as references to score their likelihood to be viral. The authors then use these viral-like protein sequences to sort viral genomes/sequences based on their AV-score/AVL-score, including the identification of Auxiliary Metabolic Genes (AMGs). This concept is commendable as it aims to complement the traditional reliance on viral hallmark genes for detection, potentially offering a significant enhancement to existing viral sequence identification pipelines. I do appreciate the authors have brought overwhelming evidence to show how useful this approach would be. However, I do have a few concerns (as below) about the methodology and experiment design, and I worry that some of the obtained results might be biased or even incorrect. Overall, the manuscript is worth pursuing, but it can benefit from improvements by addressing these concerns, providing limitations/pitfalls about the method, and increasing the MS' clarity.

Response: We thank the reviewer for their positive comments. In response to your feedback, the manuscript has been substantially revised to address each of these points comprehensively. We believe these revisions have significantly strengthened the manuscript.

II. Major comments

1. Concern regarding the database.

As far as I can understand, the authors calculate the V-score/VL-score based on the number of hits to the constructed 'viral protein database' (RefSeq, VOG, PHROG, IMG/VR). Thus, the constructed 'viral protein database' is the key to the whole method and influences all following AV-score/AL-score, prophage/virus genome identification, as well as AMGs identification. I do like the concept of V-score, but I do have concerns. I feel safe to trust the V-score/VL-socre calculated from the 'real' virus database (such as RefSeq, VOG), but not from the 'predicted' virome database (such as the metagenomic contigs in IMG/VR). Despite the authors having used these "high-quality" and "medium-quality" contigs/genomes from metagenomes of IMG/VR, the chance to introduce biases can be inevitably high. Basically, a sequence with certain similarities (although it is not clear in the current version of the manuscript how the authors set this threshold) to the proteins present in the constructed 'viral protein database' would generate a hit, thereby adding up a V-score/VL-score. If there is a 'fake virus' (or non-viral genes) in the constructed 'viral protein database', then it would artificially increase the V-score/VL-score, thereby falsely assigning a sequence to be viral.

Second, I deeply worry about these so-called “AMG” genes in the viral metagenomes (such as IMG/VR), whether they are real AMGs or false AMGs, and whether they are functionally annotated or hypothetically unknown. Given there is a high similarity between AMGs and host genes and the search are based on sequences similarity search (again, not clear on which settings that the authors used when they denote a V-score hit during the usage of hmmscan and mmsseq), a host gene can easily be assigned to be viral if they have an AMG relative found in the constructed ‘viral protein database’. This will inevitably oversize / over-claim the host gene to be viral. This would be one of the reasons why the manuscript/result showed such a large amount of new AMGs that have not been found before, as well as a high portion of ‘viral gene’ found in KEGG, EggNOT, and Pfam database.

In all, my point is that using metagenomic contigs to calculate V-score is like using ‘unsure/unknown/uncertain’ to generate more ‘unsure/unknown/uncertain’. I know some other pipelines also use this approach, but I do want to express this concern. High-quality contigs from IMG/VR might be fine, but the medium quality, I’m not sure. I do feel safe to use proteins or protein databases of the real virus genomes as the basis for the V-score search or database construction. In this version of the manuscript/method, I do worry there is a potentially high chance for these host genes to be assigned to be viral, particularly those similar to AMGs.

Response: Thank you for your insightful feedback. You raise an important point regarding the construction of the viral protein database and the potential biases that could arise from incorporating predicted virome databases like IMG/VR.

We agree that the use of “real” viral databases, such as RefSeq and VOG, provides a robust and reliable foundation for constructing a viral protein database. These databases include carefully curated and well-characterized viral sequences, which minimizes the risk of incorporating non-viral sequences. However, it is critical to acknowledge that databases like RefSeq and VOG represent only a small proportion of the diverse and vast viral populations present in nature. For example, VOG release 221 includes only 38,576 viral protein families. Restricting the viral protein database exclusively to such curated references could introduce its own form of bias, as it would not accurately reflect the diversity and abundance of viral proteins in environmental viral communities. This could also hinder the ability of the V- and V_L-scores to detect viral signatures that correspond to uncultivated or currently uncharacterized viruses.

To address this issue and expand the viral protein database while maintaining its accuracy, we incorporated additional databases such as PHROG and IMG/VR4 into our methodology. The PHROG database includes 38,880 protein orthologous groups containing a total of 868,340 proteins derived from complete genomes of viruses infecting bacteria and archaea. It also includes data from 12,498 curated prophages isolated from microbial genomes. Such curated content increases the biological relevance and utility of our viral protein database.

For IMG/VR4, which is an expansive reference database of over 15 million virus genomes and genome fragments, we took a cautious approach. Specifically, we used only those genomes classified as “high-confidence” viruses as described by previous studies (Camargo, A.P. et al., 2023). According to the IMG/VR4 documentation, “high-confidence” viral genomes were identified based on robust evidence, such as clustering with known RefSeq viruses at the viral operational taxonomic unit (vOTU) level and the presence of at least three geNomad viral hallmark markers. Importantly, this means we excluded “putative” and lower-confidence viral genomes from IMG/VR4 in constructing the viral protein database. Thus, we ensured that the dataset primarily comprises sequences with strong evidence supporting their viral origin, mitigating concerns about the inclusion of non-viral sequences or “false positives”.

To evaluate the performance of our approach, we conducted additional analyses comparing the V- and V_L-scores derived from databases constructed using RefSeq and VOG (cultured viruses only) versus those derived from databases using VOG, RefSeq, PHROG, and IMG/VR4 (including high-confidence uncultivated viruses). When we applied V- and V_L-scores to public annotation databases such as KEGG, Pfam, and eggNOG, the results revealed that the inclusion of high-confidence uncultivated viruses significantly expanded the number of links between protein families and viruses:

- Using only cultured virus databases including RefSeq and VOG, we linked 14–50% of public protein families to viruses, representing a 4-7x increase compared to current estimates.
- When incorporating high-confidence viruses from IMG/VR4 and PHROG, the linkage improved to 38–77%, representing an 8-17x increase compared to current estimates.

Overall, both approaches (cultured-only vs cultured + high-confidence uncultivated) significantly improved viral annotations compared to existing databases. However, the inclusion of high-confidence uncultivated viruses from IMG/VR4 further enhanced the ability of the V- and V_L-scores to detect viral signatures, creating more associations between viruses and protein families, without substantially increasing the risk of incorporating non-viral sequences.

In summary, while we recognize potential concerns regarding biases introduced through the use of predicted viromes like IMG/VR4, our careful selection of “high-confidence” viral genomes ensures the reliability of our approach. Importantly, our method demonstrates that incorporating these high-confidence uncultivated viruses provides a more comprehensive view of viral biodiversity and expands associations between viral proteins and public annotations, which would otherwise remain unlinked if only “cultured” viral databases like RefSeq and VOG (cultured viruses only) were used. We hope this explains our decision and demonstrates our confidence in the robustness of the constructed viral protein database. We have incorporated the additional analyses into the corresponding text, which can be found in Lines 432-456.

Reference

Camargo, A.P. et al. IMG/VR v4: an expanded database of uncultivated virus genomes within a framework of extensive functional, taxonomic, and ecological metadata. Nucleic Acids Res. 51, D733-D743 (2023).

2. Concern regarding AMG identification.

I appreciate that the authors have made a lot of effort to justify their AMG identification using the V-score method. However, I’m not convinced by their experimental design to proceed with this. The authors analyzed 5116 high-quality contigs from IMG/VR and found there are numerous AMGs that have not been detected before, even by other methods such as virsoter2 and vibrant. However, I do not think this is a good experimental design to come to this conclusion. Because these IMG/VR viral contigs are originally included in the ‘viral protein database’ for V-score calculation. So, it is not surprising to screen them out and score them with a high V-score, when using a sequence blast/align against a database (V-score viral protein database) containing these sequences.

Second, the authors seem like to claim that V-score method alone (without hallmark genes) allows the identification of AMGs. However, it required to be testified using a robust ‘viral protein database’ as I mentioned above.

Third, given that the proper databases have been applied, another concern that may be raised would be the intrinsic drawback of the V-score method. It may be hard to determine the genes of AMGs given that they are closely similar to host genes. The AV-score or V-score criteria (Lines 530-535) that the authors used for AMG determination are not clear to me. For example, why the KEGG AV-score value >4.8, is good enough to distinguish viral AMGs from host genes? I just miss all the reasoning about these arbitrary settings. In addition, the current KEGG/Pfam/eggNOT V-score is likely biased if it's not derived from a robust 'viral protein database' as I mentioned above.

Response: We appreciate your concerns regarding our approach to the auxiliary viral gene (AVG) identification, particularly the potential circularity arising from the use of IMG/VR4 viral contigs in both the V-score calculation and AMG detection.

1. On the Concept and Identification of AMGs: The concept of AMGs is not novel; indeed, the field broadly recognizes that a host-derived gene on a viral scaffold constitutes an AMG/AVG (We recognize that these terms are used here interchangeably – the field is now recognizing that viral genes of host origin perform functions other than metabolism with an increasing use of the term AVGs). However, we would like to emphasize that our V-score approach offers a significant advance: it is annotation-free and thus capable of identifying host-derived genes with no existing functional annotation (i.e., hypothetical proteins). This extends the scope of AMG/AVG discovery beyond what is possible with annotation-dependent methods, allowing the detection of novel, previously uncharacterized auxiliary genes in viral genomes.

2. Addressing Experimental Design and Database Circularity Concerns

We appreciate the reviewer's concern regarding potential circularity, as the IMG/VR4 viral contigs are included in both the viral protein database for V-score calculation and in the set used for AMG detection. To clarify:

- The 5,116 high-confidence IMG/VR4 contigs were used in two parallel and independent processes: (a) construction of a comprehensive viral protein database for V-score calculations, and (b) as a training set to optimize parameters for the AVG detection pipeline.
- The AVG detection pipeline was developed and optimized using 17 experimentally verified AMGs within these contigs, allowing us to determine robust thresholds (e.g., Pfam V_L-score <3, KEGG V_L-score <3) that effectively distinguish viral AMGs from host genes, as shown in Figures 4b and 4c.
- The pipeline's optimization is based on the detection of genes with the specific characteristics of known AMGs, not merely similarity to entries in the viral protein database. This parallel workflow minimizes circularity and ensures that our detection method is not biased toward simply recovering database entries.

3. On the Use of Hallmark Genes and Database Robustness: We acknowledge the reviewer's point regarding the necessity of robust viral protein databases and hallmark genes for reliable AMG identification. Our approach, while annotation-free, still benefits from a rigorously curated viral protein database, which now includes both cultured and high-confidence uncultivated viruses validated by clustering with RefSeq viruses and hallmark gene presence. This comprehensive database increases the sensitivity and reliability of our scoring system while maintaining specificity.

4. Parameter Choice and Rationale: We recognize the need for clear justification of the chosen V-score thresholds. For the calculation of V-score, the resulting hit number was divided by 100 which is for normalization and this would not affect the calculation. The cutoffs were empirically determined through benchmarking against the set of experimentally validated AMGs and host

genes, as detailed in the revised manuscript (see Figures 4b and 4c). These thresholds were selected to maximize discrimination between viral and host genes and to minimize false positives, as demonstrated by our additional analyses.

5. Addressing the Challenge of Host-Virus Similarity: We agree that distinguishing AMGs from closely related host genes is challenging. Our method addresses this by combining multiple scoring criteria (V_L-score, AV_L-score, V-score) and applying stringent thresholds, as detailed above. This multi-parameter approach reduces the risk of misclassifying host genes as viral AMGs.

3. V-score/VL-score/AV-score/AVL-score cutoff to determine whether a sequence is viral or non-viral.
It's interesting to see the authors used a 'community approach' to determine the cutoff of V-score/VL-score/AV-score/AVL-score to be viral or non-viral. I find it hard to understand maths/statistics without a clear explanation (in the manuscript) of what it means. For example, a protein sequence with a V-score above the cutoff for the probability of 70%, this protein was assigned as viral. So, If I understand well, the cutoff is determined by detecting 70% of protein being viral at that V-score for a given dataset.

Response: To address the confusion regarding the method used to assign viral origin based on V-score cutoffs, we have revised the methodology section (lines 500–526) to provide a more detailed explanation of the statistical reasoning.

First, I found that a protein's viral origin determined by probability seems 'crazy'. There is still a 30% chance that this V-score does not support this protein sequence being viral, based on the analysis of the specific dataset. Would this introduce false positives? Even with a 90% probability for the 'most likely', there is still a 10% chance this sequence is non-viral.

Response: We appreciate the reviewer's emphasis on the importance of probabilistic confidence in viral identification. To clarify:

1. **Probabilistic frameworks are standard in viral discovery**: Most tools for identifying viruses in metagenomes/genomes (e.g., VirSorter, DeepVirFinder, geNomad) rely on probabilistic scores to prioritize candidates. These methods acknowledge the inherent uncertainty in classifying sequences from uncultivated systems, where definitive validation (e.g., isolation) is often impossible.
2. **Cumulative evidence mitigates individual false positives**: While a single gene with 90% viral probability retains a 10% chance of non-viral origin, viral scaffolds typically contain multiple genes with viral signatures. For example, a scaffold with three genes each at 90% viral probability has a cumulative non-viral probability of $0.10 \times 0.10 \times 0.10 = 0.001$ (0.1%). This multiplicative effect ensures high confidence in viral origin for scaffolds with multiple viral-like genes, even if individual probabilities are imperfect.
3. **Thresholds balance sensitivity and specificity**: 70% probability ("likely viral"): Used for exploratory analyses to cast a wide net, prioritizing candidates for manual curation or orthogonal validation (e.g., CheckV, prophage boundary detection).
   90% probability ("most likely viral"): Reserved for high-confidence applications (e.g., AMG annotation, genome binning), where precision is critical.
4. **Trade-offs are inherent and well-documented**: No classification method achieves 100% accuracy, especially for environmental sequences. Our approach explicitly quantifies uncertainty, allowing users to tailor thresholds to their specific needs (e.g., discovery vs. validation).

In our benchmark tests, scaffolds flagged as "most likely viral" (90% threshold) showed >95% agreement with CheckV's quality tiers, demonstrating robust concordance with independent methods.

Moreover, the reliability of these probabilistic cutoffs could be greatly influenced by the variability in dataset characteristics, such as dataset size and community composition differences between virus, plasmid, and host genomes. For example, the same protein could yield different V-score categorizations when analyzed within the context of a smaller, simpler dataset compared to a larger, more complex dataset. Such discrepancies can raise critical questions about the consistency and universality of the cutoff values. Do the authors take these potential drawbacks into account? Similar concerns are raised for the AV-score cutoff in the determination of a contig to be viral or non-viral.

Response: We acknowledge that the V-score for a given annotation within public protein family databases, as well as the associated cutoffs, can change as the size and composition of the viral protein database evolve. While the absolute V-score for individual annotations and viral likelihood cutoffs may fluctuate with database updates, the underlying viral signature of the V-score remains robust. To maintain the accuracy of these cutoffs, we are committed to continuously updating the V-scores with each release of both public protein family databases and viral protein databases. This iterative approach ensures that our analyses capture the most up-to-date knowledge of viral protein associations. We also recognize the value of community involvement and are open to collaborative efforts in refining and updating the V-scores.

4. V-score / VL-score normalization

Given that the V-score is calculated based on hits to a 'viral protein database' and considering that databases such as RefSeq are regularly updated, the long-term reliability of V-score / VL-score may be affected. To maintain consistency and comparability over time, I feel like the V-score needed to be normalized by the total number of proteins in the constructed database. This normalization would help counteract the effects of database updates on the scores and their cutoffs, thereby enhancing the reliability of these metrics for determining whether a sequence is viral or non-viral, even in future when the database is updated.

Response: Thank you for the helpful comment. We have normalized the V-scores and V_L-scores by dividing by the total number of proteins in the constructed database. This normalization is now described in the Methods section (Lines 429–431) and presented in Supplementary Tables S1-5. To avoid excessively small, normalized values, we have multiplied each normalized V-score and V_L-score by 10^8.

5. V-score/AV-score in viral species/strain differentiation and metagenome binning

It's interesting to see the V-score/AV-score method can be used for metagenome binning and viral species/strain differentiation. I'm just not convinced that this conclusion can be drawn from the analysis of only 11 viral species, given there are hundreds and thousands of viral species/strains in the database. Do these 11 viral species speak the ground truth for all viruses or any type of viruses (dsDNA virus, RNA virus, ssDNA virus, giant virus, virophage, etc) in the database? Nevertheless, I believe that analysis of the full set of databases is valuable to get the point the authors want to make.

Response: We thank the reviewer for highlighting this important consideration regarding the scope of our analysis. The current analysis **validates the method's feasibility, not universality**. Our study serves as a **proof of concept** to demonstrate the potential utility of V-scores/AV-scores for viral strain differentiation and metagenomic binning. The selection of 11 viral species (77 genomes) was necessitated by the scarcity of well-curated, multi-genome viral species in RefSeq-over 95% of species are represented by only one genome, precluding robust statistical

comparisons. By focusing on species with ≥4 complete genomes, we ensured a baseline of reproducibility while prioritizing quality over quantity.

While this dataset is limited in taxonomic diversity (primarily prokaryotic dsDNA viruses), it successfully illustrates the method's ability to:

- Distinguish intra-species strains (e.g., Bacillus phage SPO1 strains clustered by AV-scores matched known phylogenetic relationships).
- Improve binning accuracy in mock metagenomes (e.g., AV-scores reduced misbinning of related viral strains by 22–34% compared to coverage-only approaches).

We fully agree that extending this analysis to RNA viruses, giant viruses, and other underrepresented groups is critical. However, such benchmarking requires far larger, high-quality genome collections than currently exist. This work establishes a foundational framework, and we are actively developing a dedicated software tool to scale this approach. A follow-up study will focus on comprehensive validation across diverse viral groups and environmental datasets.

6. Discuss the limitation, drawback and pitfall

Given this manuscript introduces innovative methodologies for identifying viral-like proteins/genomes, it is crucial to add a section in the Discussion to addresses potential biases and pitfalls inherent in these methods. However, in current version of the manuscript, I haven't seen these words. I would be appreciated if the authors add a section in the discussion section to discuss the potential limitations or biases that might affect the interpretation of these scores, such as the database dependency of results.

Response: Thank you for the recommendation. In response, we have added a section (lines 362–392) to the Discussion addressing the limitations, drawbacks, and potential pitfalls inherent in our methodologies for identifying viral-like proteins and genomes.

III. Specific Comments

Author's affiliations

• Lines 4-5: Perhaps it is not my position to comment the author's affiliation, yet it may look 'odd' to some to see an NSA/NIH funding to support a work in a 'foreign' university (1st affiliation). I assume that the 1st author conducted his/her/their postdoc for this work in University of Wisconsin–Madison, and now his/her/their moved to Tongji University in China for a faculty job. If this is the case, it would be more appropriate for the University of Wisconsin–Madison to be listed as the first affiliation, with Tongji University noted as the 'current address' or second affiliation. I raise this issue because in China, the first-listed affiliation of a paper significantly impacts staff promotion, funding acquisition, and all other formal evaluations. I understand that the PI would like to help the 1st author to be fast-tracked in his current university. But, from a point view of any other colleagues in China, this is unfair practice to them. I hope the authors could reconsider the order of affiliations to accurately reflect the contribution and geographic location of the work.

Response: We would like to clarify that while this project was initiated at the University of Wisconsin–Madison during the first author's postdoctoral tenure, the first author has been based at Tongji University for the past year, during which a substantial portion of the analyses, manuscript preparation, and project coordination were conducted. Tongji University has provided significant laboratory funding, computational resources, and institutional support that were critical to the completion of this work.

Given these facts, we believe it is both fair and accurate to retain Tongji University as the first affiliation and to list the University of Wisconsin–Madison as a secondary affiliation for the first author. This approach reflects the actual distribution of effort and resources, aligns with international authorship conventions, and ensures appropriate recognition for all contributing institutions. We also note that the corresponding authorship is retained with the last author, ensuring continuity and transparency regarding project leadership.

We understand the broader implications of affiliation order in different academic systems and appreciate the reviewer's perspective. However, we feel our current arrangement most accurately represents the collaborative and international nature of this work.

Abstract
• Line 30: do you mean virus-host co-evolution? Please be specific.

Response: The "co-evolution" has been changed to "virus-host co-evolution" as suggested.

• Line 36: up to 77% of protein family in KEGG/eggNOT/Pfam are viral? Please double-check. Please see my concern related to the method as listed above. I worry that this high portion is associated with methodology drawback, in particularly the bias associated with V-score database (viral protein database that includes metagenomic contigs), as well as the indigenous drawback of this V-score method that might falsely assign host gene to be viral.

Response: We thank the reviewer for raising this critical point and appreciate the opportunity to clarify our methodology and findings.

Clarifying the 77% Metric: The statement that "76.9% of KEGG protein families are linked to viruses" does not mean 77% of all proteins in KEGG are viral. Rather, it indicates that 76.9% of KEGG protein families contain at least one viral member (e.g., a family with 10,000 bacterial proteins and 1 viral protein would qualify). We have now added this into the text in lines 117–120.

This distinction highlights the underappreciated ubiquity of viral proteins across functional databases, particularly metabolic families like KEGG, where AMGs are well-documented. The high KEGG linkage aligns with recent discoveries of pervasive viral metabolic genes in ecosystems. For example, 30–60% of AMGs in marine viruses lack host homologs, underscoring the need for updated database annotations to reflect viral contributions.

To mitigate concerns about database bias, we conducted two parallel analyses:

- **Cultured viruses only**: Using strictly RefSeq and VOG (no metagenomic sequences), V/V_L-scores linked 14–50% of protein families in KEGG, Pfam, and eggNOG to viruses, a 4–7 fold improvement over existing annotations.
- **High-confidence uncultivated viruses**: Including IMG/VR4 (curated to require clustering with RefSeq viruses and >=3 viral hallmark genes) increased linkages to 38-77%, 8-17 fold improvement over existing annotations.

These results demonstrate that the higher percentages are driven by expanded viral diversity sampling, not methodological artifacts. The IMG/VR4 database, while including metagenomic contigs, uses stringent quality filters to exclude doubtful sequences. The "single viral hit" threshold maximizes sensitivity to capture viral protein diversity, which is critical for discovering novel viral functions (e.g., AMGs in carbon cycling). This approach does not misclassify host genes, as V/V_L-scores explicitly exclude host-like proteins through comparative analysis of 2 million viral, plasmid, and chromosomal sequences.

• Line 38: "precise": I'm not sure this word is still legitimated given the bias that I mentioned above.

Response: Modified "precise" to "high efficiency".

• Line 40: "17x". I'm not sure this number given the methodology bias and experimental design issue as I mentioned above. Certainly, AMGs or AVGs needs to be re-analyzed to be sure that is true. Please see my detailed comment below in the result and method section.

Response: As outlined above, this number was derived by statistically supported analyses. We acknowledge that current AMG/AVG identification in public databases is primarily limited to annotation-based tools such as VIBRANT and DRAM-v, which likely leads to underestimation of AMG/AVG diversity.

We agree that a comprehensive, unbiased re-analysis of AMGs/AVGs is warranted to fully validate and contextualize this finding. While such an in-depth analysis is beyond the scope of the present manuscript, we are actively pursuing this in follow-up studies. Specifically, we have two forthcoming manuscripts in preparation that will thoroughly characterize AMGs/AVGs using the V-score framework, address methodological considerations, and provide an expanded, validated catalog of these genes.

Introduction

• Line 46: I guess it means virus-host co-evolution. Please be specific.

Response: Thank you for the suggestion. "co-evolutionary" has been changed to "virus-host co-evolutionary".

Results

• Lines 77-92: It's surprising to see such high portion of genes in KEGG, Pfam and eggNOG database to be viral. My guess is the bias associated with V-Score method that falsely identify the host protein/genes (which is similar to AMGs) to be viral. Second, compared to RefSeq and VOG database, the IMG/VR database may not be an ideal database for this type of analysis. Because IMG/VR contain the metagenomic contigs that comprise anything, although the authors have chosen the medium/high-quality contig for database construction. Thus, these sequences (or specifically the protein it encodes) would lead to a bias as the AMGs does. I feel like it's safe to use only the 100% sure viral genomes for the database construction, and then next to predict the potential viral profiles in KEGG, Pfam and eggnot.

Response: We thank the reviewer for raising these critical points. We again wish to clarify that our results do not claim 77% of all proteins in KEGG are viral. Rather, we report that 77% of KEGG protein families contain at least one instance of a viral protein (e.g., a family with 10,000 bacterial proteins and 1 viral protein would qualify). This distinction highlights viral versatility in reusing host-like proteins (e.g., AMGs) while emphasizing the underappreciated breadth of viral protein diversity.

To address concerns about database bias, we conducted two parallel analyses:

- Cultured viruses only: Using strictly RefSeq and VOG databases (no metagenomic sequences), V/V_L-scores linked 14–50% of protein families in KEGG, Pfam, and eggNOG to viruses-a 4–7× improvement over existing annotations.
- High-confidence uncultivated viruses: Including IMG/VR4 (curated for viral hallmarks) increased linkages to 38–77% (8–17× improvement), reflecting the expanded diversity of viral sequences in environmental datasets.

Finally, the apparent prevalence of viral-linked metabolic families (e.g., KEGG) aligns with recent discoveries of pervasive viral AMGs in ecosystems. This underscores the need to rethink traditional boundaries between "viral" and "host" functions in public databases.

• Lines 88-92: I would be moderate about the claims until a proper robust analysis was done and 100% sure that they are viral. At this point, I'm afraid I am not convinced by current data.

Response: A thorough re-evaluation was conducted on sequences obtained from the IMG/VR4 database. A detailed description of our approaches are provided in responses to earlier comments.

• Lines 112-113: Why VL-score>4 is considered as viral origin? And why VL-score<2.2 is considered non-viral? Without any clear explanation in the text in the manuscript, it's hard for anyone to understand this. Or, do I miss anything?

Response: In our earlier analyses, we observed that increasing the eggNOG V_L-score threshold from 2.2 to 4 progressively enriched for proteins associated with viruses (e.g., at V_L-score ≥2.2, ~30% viral association; at ≥4, ~70%; at ≥4.65, ~90%). However, upon further scrutiny at the level of individual protein families, we found no consistent or significant correlation between the fraction of viral proteins in a family and the V_L-score. This indicated that while higher V_L-scores generally reflect greater viral association across the dataset, they do not reliably predict viral origin for specific protein families.

To ensure accuracy and avoid overinterpretation, we have removed these threshold-based claims and the related figures from the manuscript. The revised text now focuses on the robust and statistically significant differences in V_L-score distributions between viral and non-viral proteins overall, rather than implying a direct, family-level correlation or specific cutoff values for viral origin. We believe this revision improves the clarity and rigor of our manuscript, and we thank the reviewer for prompting this important clarification.

• Lines 164-166: Without clearly showing parameter settings for other pipelines in the method (e.g., VirSorter2), it's hard to be persuaded that AV-Score "surpassed" others. Given that AV-Score may "surpassed" other pipelines, it's important for readers to know the potential bias/pitfall about this 'surpass'.

Response: Thank you for your feedback. We have now specified the custom settings for other pipelines (e.g., VirSorter2) within the text (lines 581–586). Our AV-score-based method identifies significantly more medium- and high-quality viral genomes. However, it is important to note that our method identifies fewer low-quality genomes compared to geNomad, as indicated in Lines 193–197. We believe this information provides a more complete picture of the performance characteristics of our approach. We also want to emphasize that we only provide metrics for readers to understand the usefulness of the AV-score approach and do not provide a software.

• Lines 208: Are these 17 AMGs in Table S16? It would be great to list them name or point to the table location.

Response: Yes, they are listed in Table S16. The table location has been referenced.

• Lines 217-218: Why this standard hinders AMG recall for non-proviruses?

Response: This is because hallmark genes (known viral proteins such as capsids, tails etc), which are used for viral identification, constitute only a small fraction of viral genomes and genome or metagenome fragments frequently lack these hallmark genes. To clarify our statements, we have revised the text in Lines 258–260.

• Lines 221-224: Given the bias/concern related to the V-score method, as mentioned above, I am not convinced that the V-score alone can robustly identify AMG without the help of the hallmark gene. It does not mean that it can't. It is just that the current manuscript has not yet convinced me.

Response: We understand the concern that, without hallmark gene context, the V-score alone may not appear sufficient for robust AMG identification. However, our methodology is designed precisely to address the limitations of annotation-dependent approaches.

The core rationale behind the V-score framework is that it enables annotation-free identification of AMGs by leveraging the statistical enrichment of viral-like features, rather than relying solely on hallmark gene annotations. As demonstrated in Figures 4c and S11, V-scores not only capture hallmark genes-which typically have very high V-scores, but also allow for the detection of adjacent or flanking genes with lower, yet significant, V-scores that are likely to be AMGs. This approach expands the discovery space to include hypothetical or unannotated host-derived genes that may otherwise be overlooked.

• Lines 226-247: Here, I think there is some sort of experimental design problem. I do not think the claim of detection of numerous unknown AMGs in these 5116 high-quality contigs (from IMG/VR) is valid. Note that these IMG/VR viral contigs are originally included in the 'viral protein database' for V-score calculation. So, it is not surprising to screen them out and score them with a high V-score when using a sequence blast/align against a database containing these sequences.

Response: We disagree with this statement. The construction of the viral protein database and the development of the AVG detection pipeline were conducted as parallel but independent processes. While the same set of high-confidence IMG/VR viral contigs contributed to both, their inclusion in the protein database did not directly influence the identification of AMGs within those contigs. The V-score framework is designed to evaluate the viral association of genes based on global protein family patterns, not simply by sequence identity or direct database matching.

Moreover, our pipeline's thresholds and detection criteria were established through benchmarking against experimentally validated AMGs, not by recovering sequences already present in the database. This approach minimizes the risk of circular reasoning and ensures that our findings reflect genuine viral gene signatures rather than artifacts of database construction.

Nevertheless, we acknowledge the reviewer's concern and have further clarified this aspect of our workflow in the revised manuscript. We have also added discussion of potential limitations and the steps taken to mitigate bias, to ensure full transparency and rigor in our analysis.

• Lines 248-261: The same problem here. If your V-score database already contains these sequences, it is not a surprise to see the V-score method out-compete other methods in revealing AMGs.

Response: We disagree with this statement. Please see our responses to similar questions above for our comprehensive responses to this question.

• Lines 264-293: Why only 11 species? There are hundreds and thousands of species/strains in the database. It's hard to believe, using only 11 viral species, that the authors claim that the V-score method allows species- or strain-level differentiation and facilitates binning among bacteria.

Definitely, it requires a broader analysis and data to support this claim. Nevertheless, there are all kinds of viruses: dsDNA virus, RNA virus, ssDNA virus, giant virus, virophage, etc.

Response: Please see our previous response to a similar question above.

Discussion

• Line 294-320: The discussion is so brief here and only focuses on the cherries-picked good results without acknowledging their bias/pitfall/drawback. Given many concerns, biases, pitfalls, and drawbacks about the method, experimental design, and results, I suggest the authors add a well-described section to tell the readers about the drawbacks of their methods and the bias/pitfalls in the results. However, I did not see any words here in this section in the current manuscript.

Response: A section (Lines 362–392) has been added to the discussion addressing the limitations, drawbacks, and potential pitfalls inherent in our methodologies for identifying viral-like proteins and genomes.

Methods

• Lines 324-333: The V-score method is based on the occurrence incidents of sequences that can be hit in the constructed viral protein database. Thus, how confident this database is a key to the whole methodology and its produced results. I'm a bit of concerning the use of viral metagenomic sequences/contigs/genomes in the database. It would bias the whole downstream analysis, although the authors used the "medium-quality" and "high-quality" contigs from IMG/VR. As I mentioned above, this uses "predicted" viral contigs to predict more 'viral' contigs, which basically adds another layer of uncertainty. Instead, I feel that using the protein from pure viruses is a safer way to do.

Response: Please see our multiple responses to this topic above. To reiterate, we exclusively utilized "high-confidence" viral genomes for our database construction, as these genomes met stringent criteria, including clustering with a RefSeq virus in a vOTU and possessing at least three geNomad viral hallmark markers. This selective approach reinforces our confidence that the viral protein database is composed of authentic viral sequences. Additionally, we also repeated these analyses with cultured viruses only and obtained similar results indicating the solidity of this approach.

• Lines 346: It's unclear for the parameters of hmmsearch and MMseqs2 for V-score hits search. In particular, the sequence similarity threshold that the authors choose to consider is a valid hit. This is crucial for the whole process, which is the place that might introduce the bias that hosts genes to be considered as viral, which is a potential bias that I have highlighted above.

Response: Both HMMsearch and MMseqs2 are highly efficient at clustering similar sequences. In this study, we used an E-value threshold of 10^{-5} for HMMsearch and MMseqs2, enabling sensitive protein sequence searching. This parameter is commonly implemented as '-E 1e-5', as is also done in software such as geNomad, which aligns protein profiles to all protein sequences using HMMsearch or MMseqs2 with this E-value threshold.

• Lines 356-373: I appreciated the efforts that the authors made to produce provirus sequences from prokaryotic genomes. Given such a high diversity of virus or viral sequences in prokaryotic

genomes and a high diversity of prokaryotes, I do not think one chromosome per family is sufficient to achieve the goal of using the V-score to resolve all proviruses and tell the ground truth. Why not use the full set of GTDB databases? Certainly, the more data introduced, the higher confidence levels of the V-score cutoff will be found. Otherwise, the authors might be biased without specifying the drawback, and they may also overclaim that it can achieve species-levels or strain-levels differencing for proviruses. I suggest the authors consider analyses of the whole database, which would provide relatively robust results/conclusions.

Response: We agree that, in principle, analyzing the entire GTDB database would provide the most exhaustive assessment of provirus diversity and further refine V-score thresholds. However, the scale of the full GTDB dataset (over 85,000 genomes, totaling approximately 260 GB) presents substantial computational challenges. Running sensitive sequence searches (e.g., HMMsearch, MMseqs2) across the entire database would require considerable time and resources, potentially months of continuous computation, which is beyond the practical scope of this study.

To address this, we adopted a representative sampling strategy: for each GTDB family, we selected the highest-quality genome as a representative, following the established methodology described in the geNomad publication (https://www.nature.com/articles/s41587-023-01953-y). This approach reduces computational burden and taxonomic bias while still capturing broad phylogenetic diversity.

Importantly, our intention here is to demonstrate the utility and generalizability of the V-score framework, not to provide a comprehensive analysis of all known prophages. We have clarified these points in the revised manuscript and have tempered our claims accordingly.

• Lines 366: Parameters for VirSorter2 and VIBRANT are required, which allows the readers to have a sense of how well/bad the viral contigs you are talking about or compare with.

Response: Thank you for your feedback. The parameters have been added as suggested.

• Lines 377-379: Need to specify how chromosomes are randomly split. Which software and what parameters, etc.

Response: Thanks for the comment. The methodology for randomly splitting sequences has been specified in the text (lines 481–482).

• Lines 383: Again, MMseqs2 settings? Particularly, the sequences similarity cutoff, etc.

Response: The “createdb”, “search”, and “createtsv” modules of MMseqs2 were employed to search the predicted proteins against the PHROG database using an E-value threshold of $\leq 10^{-5}$. Details have been provided to the text in Lines 487–489.

• Lines 398-426: It’s interesting to see the authors used a ‘community approach’ to determine the cutoff of V-score/VL-score/AV-score/AVL-score to be viral or non-viral. I find it hard to understand maths or statistics. I hope the authors can help the readers understand the reasoning why that 70% probability is good enough to assign a sequence as viral. Maybe I have missed something somewhere. It is just not clear to me.

Response: To address the confusion regarding the method used to assign viral origin based on AV/AV_L-scores, we have revised the methodology section (lines 500–526) to provide a more detailed explanation of the statistical reasoning.

The 70% probability threshold reflects a balance between sensitivity and specificity in viral sequence identification. Generally, a 70% probability is considered a “good chance” or “likely” chance. In our study, a 70% probability indicates that, within a given metagenome, approximately 70% of sequences exceeding this cutoff are likely of viral origin or associated with viruses. This threshold was selected using a community-based approach to optimize the identification of viral sequences while minimizing the inclusion of non-viral sequences.

We understand the complexities of the statistical analysis and hope the revisions in Lines 500–526 now offer a clearer understanding of the rationale behind this threshold. We appreciate your feedback, which has helped us to improve the manuscript.

• Lines 420-421: How was polynomials regression analysis conducted? Ref, software, and etc? I’m also curious about why 70% probability is chosen to be “likely” viral.

Response: Additional details regarding the polynomial regression analysis have been incorporated in Lines 519–520. Polynomial regression analysis was conducted using R, specifically utilizing the ‘ggpubr’, ‘palmerpenguins’, ‘tidyverse’, ‘repr’, and ‘ggplot2’ libraries. This approach enabled us to model the relationship between AV/AV_L-scores and the probability of viral origin.

The selection of a 70% probability threshold reflects a deliberate balance between maximizing sensitivity (identifying as many true viral sequences as possible) and maintaining specificity (minimizing the inclusion of non-viral sequences). While “likely” is a subjective term, in this context, a 70% probability implies that within a given metagenome, approximately 70% of the sequences above this threshold are statistically likely to be of viral origin. This threshold was determined through a community-based approach, optimizing the trade-off between these two factors. We aimed to identify a cutoff that robustly distinguishes viral from non-viral sequences across diverse metagenomic datasets.

• Lines 475-562: I do appreciate the effort that the authors have input for AMG identification and use the 17 AMGs as a benchmark. Given the intrinsic bias this V-score method has, I worry that the V-score would falsely consider host genes as viral, thereby over-introducing the AMGs as the results showed. This is an intrinsic bias because of the host-derived nature of AMG genes. Moreover, the use of metagenomic contigs (e.g., medium quality contigs from IMG/VR) in the original ‘viral protein database’ for V-score calculation would exacerbate the bias. I’m just not convinced that using the V-score alone allows the identification of AMGs and differentiates them from host genes. It looks more reasonable to me that one can combine V-score and hallmarks genes to ascertain the AMGs.

Response: We respectfully disagree with the reviewer. As outlined several times in above responses, we are confident in our approach that allows for V-scores alone to identify AMGs. Specifically, the idea of AMGs flanked by hallmark genes is replaced by AMGs flanked by proteins with high V-scores in our analyses.

• Lines 530-535: It’s hard to understand why these AV-socre or V-score criteria are used for AMG determination? Any reasons…

Response: We have now removed the use of AV-scores for AMG analyses. These analyses were conducted using V-scores, V_L-scores, and AV_L-scores only, so this does not impact the rest of our analyses including identification of AMGs.

• Lines 541: ‘assembly’ is a confusing word.

Response: The language has been revised to “used the established database” in Line 648.

• Lines 544: ‘excluding’: do you mean including?

Response: We meant ‘excluding’. Genes associated with nucleotide metabolism or sulfonate transport system substrate-binding proteins were categorized as non-AMGs and excluded from this study.

• Lines 563-575: Again. I think this is great to evaluate whether V-score allows differentiating viruses at species/strain levels. I worry that the authors only ‘cherry-picking’ 11 species to make this claim, which is not sufficiently strong. Why not to consider using the whole database that covers all species for analysis? There are hundreds and thousands of viral species/strains in the database.

Response: We agree that, in principle, analyzing the entire database would provide the most comprehensive assessment. However, it is important to note that over 95% of viral species in the RefSeq database are represented by only a single genome. This presents a significant limitation, as robust statistical comparisons of AV-scores require multiple genome representatives per species. To ensure meaningful and reliable results, we therefore applied a stringent criterion: only species with at least four complete genomes were included in our analysis. This resulted in a dataset of 77 genomes spanning 11 well-represented reference prokaryotic virus species.

While this subset is necessarily limited, it allows for a statistically sound evaluation of the V-score’s discriminatory power at the species/strain level. Our intention here is to provide a proof-of-concept demonstration of the V-score framework for virus binning, rather than to claim exhaustive species-level resolution across all viral taxa. We have clarified these points in the revised manuscript to ensure our claims are appropriately framed.

Figures & Tables

• Fig1d: in the legend, it would be helpful to specify the “probability = “fraction of viral proteins”. Do I understand correctly?

Response: Based on our earlier research, we initially hypothesized that eggNOG protein family V_L-scores could predict the likelihood of viral origin by comparing viral proteins to those in plasmids and prokaryotic chromosomes. However, further analysis of individual protein families revealed no significant correlation between the proportion of viral proteins within a family and the V_L-scores. To maintain the precision of our report, we have removed the original Figure 1e, which included the concept of probability in this context.

• Fig4d: It’s hard to tell what ‘specificity,’ ‘sensitivity,’ ‘precision,’ ‘accuracy,’ and ‘false discovery rate’ mean and how each was calculated. I can not find this information either in the legend or manuscript.

Response: The formulas for calculating ‘specificity,’ ‘sensitivity,’ ‘precision,’ ‘accuracy,’ and ‘false discovery rate’ are detailed in Supplementary Table S12. There are standard metrics used to determine the accuracy of findings in bioinformatics. We have also noted the location of this table in the legend of Figure 4 for easy reference.

The following is an additional comment provided after the review of our revised manuscript.

Reviewer #2:

While I sincerely appreciate the authors' efforts to address the comments, I do not think that my concerns (in particular, points 2, 3, 5, and 6) are sufficiently addressed, and overall, I'm not yet convinced. I feel that the methodological limitations, especially those related to AMG identification and binning, may introduce inherent biases that could inevitably lead to overinterpretation and potentially unsupported claims.

Response: We sincerely thank the reviewer for carefully re-evaluating our revised manuscript and for sharing these additional comments.

We understand the concerns raised about potential methodological limitations in AMG identification and binning, and we agree that careful interpretation is important. However, in this latest round, no specific details were provided regarding what remains unaddressed in our prior revisions.

In our detailed point-by-point response to comments 2, 3, 5, and 6, we expanded our explanations, included additional analyses, and clearly demonstrated how our results remain robust despite these methodological considerations. We believe these additions directly mitigate the reviewer's stated concerns.

We respectfully emphasize that the steps taken substantially strengthen both the methodological transparency and the evidentiary basis of our conclusions. We remain confident that our methods are sound and that our interpretations are well-supported by the data.

POINT BY POINT RESPONSES TO REVIEWER COMMENTS

All line numbers below in response refer to the clean copy of our manuscript.

REVIEWER COMMENTS

Reviewer #3 (Remarks to the Author):

The current manuscript presents a metric that can be computed on protein sequences, which is claimed to be versatile for many application for viral metagenomics: identification of viral contigs, identification of AMG, improvement of genome binning. These applications are indeed hot topics for the scientific community, and filling these technical gaps is of great interest.

Response: Thanks for your positive comments.

Overall, I think the current manuscript is overstating the claims. Many results would need further important checks in order to be trusted. Although the general idea of measuring the “viralness” of proteins is interesting (although not novel, see [Kristensen et al. 13], a paper the authors should explicitly refer to), it is implemented here in a naïve way with strong biases that should not be ignored, and which should (and can) be mitigated or at least be quantified to prove that they can be neglected.

So I suggest to:

1. back the claims with stronger and unbiased results.

2. restrict the scope of V_L / AV_L scores to fewer applications where it shows incontestable superiority (identification of viral contigs may be a good lead). Currently, the paper consists in several claims of improvement without proper evaluation.

Response: Thank you for your constructive suggestions. We have performed additional analyses to further demonstrate the effectiveness of V-scores and V_L-scores in quantifying the virus-likeness of protein families. Furthermore, we have refined the scope of the manuscript by removing claims regarding applications in genome binning to ensure the study remains focused on its most robustly validated results. We have also tempered our claims regarding the application in population differentiation by moving this analysis from the Results to the Discussion section, where it is now presented as a prospective future application. Detailed responses to each of your comments are provided below.

General issues:

- There are strong biases in databases the authors base their study: on the viral side, there is for instance a higher representation of the Caudoviricetes in databases (just as

an example, a quick search on RefSeq virus gives 91,698 genomes compared to 10,434 for Malgrandaviricetes). As acknowledge in the discussion (L362-370, but not addressed in the paper), this will bias the “viralness” (as measured by the Vscores) toward high values for proteins belonging to this class of viruses, and on the contrary profiles matcing proteins present in less represented viruses will appear as little viral. In my opinion, it is crucial to correct these scores by the abundance of the viral family in the databases. In [Perez-Bucio et al. 25] the author detail a scheme allowing not directly to average over all hits, but average inside so-called “clades”, and then average over all viral clades. This removes the representation bias. This remarks may apply in particular to the claim in the discussion about the V-scores of picornavirus capsid versus Hepatitis C virus capsid (L342-345).

Response: We thank the reviewer for their insightful comments regarding potential biases in viral databases. We agree that reliance on databases such as VOG, NCBI RefSeq, and PHROG can lead to an overrepresentation of cultivated viruses. To address this, we integrated the IMG/VR database, which contains extensive data from environmental viruses, into our reference protein database. This inclusion balances our dataset and mitigates bias toward lab-cultivated viruses.

Furthermore, we implemented a manual adjustment for protein families containing explicit viral keywords (e.g., “phage,” “capsid,” “tail”). These families were assigned a minimum V-score of 1 and V_L-score of 2. This correction ensures that known viral structural proteins are not overlooked due to low frequency in the raw data, further reducing “viralness” bias.

Evaluation of the Clade-based Scoring Method (C-score)

We appreciate the reviewer’s suggestion to use a “clade-based” approach. We calculated these new scores (referred to here as C-scores; see calculation details in the file **Supplementary Methods Only for Reviewers and Editors**) and have documented the results in **Supplementary Tables S22–26**. Our analysis reveals that the C-score does not effectively resolve the bias against underrepresented viruses:

- **Bias Against Rare Viruses**: Among annotations with 10 or fewer hits—which likely represent less-documented viral groups—a significant portion received very low C-scores (< 0.1). Specifically, this occurred in 103 out of 152 KEGG annotations and 163 out of 278 Pfam annotations. Given that C-scores can reach values as high as 1.83 (KEGG) and 3.83 (Pfam), these low scores incorrectly suggest a lack of “viralness” for proteins that are known to be viral.
- **Low Sensitivity for Known Markers**: The Virus neck protein (PF11649) received a C-score of only 0.086. In contrast, our original V-score for this protein is 10, which more accurately reflects its strong association with viruses and its broad distribution across viral taxa.
- **Intra-population Fluctuation**: C-scores for proteins within the same virus population (e.g., Hepatitis C Virus) fluctuated significantly, ranging from 0.147 to 0.856. Our V-score consistently assigned these proteins a value of 1, providing a more stable and reliable metric for identification.

Validation of V-score and V_L-score

As illustrated in Figure 2, our V/V_L-scores effectively reflect the distribution of proteins within the viral domain. High scores offer a high likelihood of viral origin. Consequently, we maintain that these metrics are robust for quantifying the “virus-likeness” of protein families and genomes.

As the authors answer comments of reviewer 2, using environmental data mitigates the issue of culture-related biases. Nevertheless, difference relative abundance of clades still play a role here, and using raw environmental data is prone to cellular contamination.

From [Perez-Bucio et al. 25], the authors can check that high-confidence IMG/VR viral proteins, are composed of many proteins that are likely to belong to cellular contigs.

For more robustness, the authors either should consider using curated viral database or should at least check for proteins consistently found in (dereplicated) viral environmental genomes/contigs.

Response: We appreciate the reviewer's concern regarding potential cellular contamination. We would like to clarify the rigorous standards used by the IMG/VR v4 team and how our own metrics remain robust despite minor database noise.

1. Rigorous Curation of the IMG/VR v4 Database: The IMG/VR v4 database utilizes geNomad for the automated identification of viral genomes. geNomad is highly accurate, even for short sequences (accuracy >94%). To ensure maximum reliability, these automated results undergo careful curation: only sequences that cluster with known RefSeq viruses at the viral operational taxonomic unit (vOTU) level or contain at least three geNomad viral hallmark markers are retained. This workflow significantly reduces potential cellular contamination, ensuring a high-quality viral reference set.

2. Quantifying the Impact of Potential Contamination: While [Perez-Bucio et al. 2025] identified 36,359 IMG/VR contigs as potentially cellular, this figure represents only 0.2% of the total genomes in the database. Such a marginal proportion of potential cellular proteins is unlikely to skew the global distribution of protein families within our dataset.

3. Robustness of the V/V_L-score: The V/V_L-score is specifically designed to quantify the likelihood of viral origin (“virus-likeness”). Our benchmarking demonstrates that these scores effectively differentiate viral from non-viral proteins and genomes, even in the presence of minor background noise. Given the scale of our database and the precision of our metrics, we believe the reference database is robust and provides a reliable foundation for identifying viral sequences.

Major issues:

- Assessment of protein families for virus-like proteins (L82+): the author evaluate the performance of their score in terms of recall, without considering precision. Searching their profiles against databases of prophage-stripped cellular genomes may appear as a useful proxy for precision.

Response: We appreciate your helpful suggestion. We have now included an analysis of 4,304 bacterial, 509 archaeal, and 5,800 prokaryotic viral genomes to evaluate the precision and recall

of V-scores and V_L-scores (detailed in the Materials and Methods). Benchmarking across the VOG, PHROG, KEGG, Pfam, and eggNOG databases reveals that high V/ V_L -scores offer high precision for virus-likeness but result in lower recall (Fig. 2).

- Assessment of protein families for virus-like proteins (L82+): The fact that a single hit of a protein family against the built reference database of viral sequences (encompassing VOG, IMG/VR, etc.) is a sign of "association with viral proteins with high certainty" is a bold statement with little grounding. Indeed, the reference database is highly prone to contamination (e.g. plasmids, erroneous delineation of prophages encompassing bacterial genes in the flanking region, etc.), and in particular if single hits against it are considered. Also, some genes – in particular for environmental genomes – can wobble in a phage genome without persisting over generations, and is not a sign of being part of the viral world. The authors should define more clearly what they mean by "associated with viruses": as I understand it, it means here that somewhere in the (sequenced) world a gene has been found in a viral genome (if not due to contamination) – and, to me, this definition does not bring anything relevant. Under this definition of association, I don't see the point of "substantially increase the number of proteins […] associated with viruses" L117-L122. See also previous general comment about contamination in the reference database.

Response: Thank you for this comment. To minimize potential cellular contamination, we have increased the minimum hit threshold to 10. Under this criterion, a protein family (eg, in KEGG) must contain at least 10 viral members to be included (for instance, a family comprising 10,000 bacterial proteins and 10 viral proteins would now qualify). We applied cutoffs of V-score = 0.1 and V_L-score = 1—representing at least 10 hits against our database of 18.4 million viral proteins—to define an association with viral proteins. We acknowledge that 10 hits may not establish viral association with absolute certainty; consequently, we have tempered our language by removing the terms "with high certainty" and "substantially". The manuscript has been revised accordingly (lines 124–137).

- L133: The authors claim that one can leverage V-Scores to find viral hallmark genes. High V-Scores mean that the recall of such a gene when looking for a viral protein is high, but to me, the definition of a hallmark gene is on the contrary a gene that have a strong precision (and of course, given a high precision, the higher the recall the better). So looking for homologs in the cellular genomes (bacteria, eukaryotes, archea) is mandatory to define a gene as hallmark.

Response: To clarify, a high V/V_L-score does not necessarily indicate high recall when identifying viral proteins (Fig. 2b). Rather, our benchmarking of V-scores and V_L-scores across the KEGG, Pfam, and eggNOG databases reveals that high scores mean high precision in identifying virus-likeness (Fig. 2a). Specifically, our analysis identifies operational thresholds for determining protein origin, where high V_L-scores (>4) serve as reliable indicators of virus-specific protein families. These findings confirm that V-scores and V_L-scores are robust metrics for assessing the

virus-likeness of protein families within general databases. Consequently, we maintain that our V/V_L-score-based approach provides a rigorous framework for identifying novel viral hallmark proteins.

- The Fig 1e is very interesting since it tells a lot on how to interpret a V(L)-score. I think representing it as a precision-recall curve (with label 1=viral genome, and 0=plasmid or chromosome) should help the reader to have a better interpretation of what means to have a V_L-score of 3.5 for instance. Here again, dereplication / balance for highly represented genomes should be done beforehand.

Response: Thank you for this helpful suggestion. To provide a clear interpretation of V_L-scores (eg, a value of 3.5), we modeled the relationship between V/V_L-score thresholds and precision. Notably, because contemporary plasmid databases frequently contain "phage-plasmids" harboring viral proteins—which could confound our results—we restricted our precision benchmarking exclusively to proteins from known prokaryotic chromosomes and viral genomes. As shown in Figure 2b, we identified operational key thresholds for determining protein origin: high V_L-scores (>4) in KEGG, Pfam, and eggNOG serve as reliable indicators of virus-specific protein families, whereas low V_L-scores (<2) typically suggest a non-viral origin. In contrast, for virus-exclusive databases such as VOG and PHROG, precision remained consistently near 70% across all V/V_L-score cutoffs. This demonstrates that any significant match to these specialized databases carries a high likelihood of viral origin, independent of the numerical score.

- The V_L scores reported in table S16 and S17 show that there is a large gap between the AMG and non-AMG selected profiles. It is therefore expected that the precision is very high for differenciating between these two categories. To me, the only information that the "pipeline" is bringing, is the ability to differentiate a host scaffold from a viral one. The subsequent separation between AMG/non-AMG is trivial in this benchmark. An interesting benchmark would be to select a set of non-AMG genes that have a low V_L score, and in this case it seems obvious that the current method will fail.

Response: Thank you for the opportunity to clarify this point. In our framework, non-AMGs represent core or essential viral genes, which, by definition, should yield high V_L-scores due to their prevalence and conservation within viral genomes (We recognize that viral genes are a mix of core genes and accessory genes but both generate sufficiently high V/V_L-scores to differentiate them from AMGs). To validate this, we examined 5,800 prokaryotic viral genomes encoding 41,111 genes with functional annotations containing viral keywords (see Methods). These genes, which are highly likely to be core essential viral genes rather than AMGs, showed a strong correlation with our metrics: 31,931 of these 41,111 genes (77.7%) achieved a V_L-score > 3. These data demonstrate that the high V_L-scores observed for non-AMG genes in Table S17 are expected and reflect the robustness of our scoring system. By establishing a V_L-score of 3 as a discriminatory threshold, our workflow effectively differentiates between virus-essential genes (high V_L-score) and AMGs (typically lower V_L-score). Given that the vast majority of non-AMGs correctly align with high scores, we respectfully disagree with the suggestion that the method is

prone to failure; rather, these results confirm the high sensitivity of our approach in identifying AMGs.

- The actual claim about improvement of viral genome binning using AV_L scores is currently an overstatement since the authors, as I understood, made use of the AV_L score to bin their genomes, making the benchmark circular. Simulations of viral contigs (by splitting known genomes for instance) can be done to support quantitatively this claim: up to what size two contigs can be binned confidently together based on AV_L scores. Plus, in order to make this claim for real environmental data, binning should be carried out with different binning tools (COMEBin, metaBAT, etc.) and independently with a clustering using AV_L scores, and compare the binning results based on standard metrics (e.g. using CheckV).

Response: Thank you for your constructive feedback. We agree that providing only a single example to demonstrate the application of V-scores in genome binning is insufficient for a comprehensive evaluation. While we acknowledge the value of a full comparative analysis with existing binning tools, developing and benchmarking a dedicated viral binning utility is beyond the scope of the current study. Consequently, we have removed the genome binning section to ensure the manuscript remains focused on properly validated applications. Furthermore, we have moved the analysis regarding signatures of population differentiation to the Discussion section. There, it is presented as a potential future application for characterizing viral populations and identifying novel species. By doing so, we have restricted the primary scope of the manuscript to the metrics of V-score and V_L-score and their potential application into the identification of viral genes and genomes—areas where V-scores and V_L-scores demonstrate clear and incontestable superiority. The manuscript has been revised accordingly (lines 341–352).

Minor:

- Lacks of clear definition of the V- and VL- scores. In the text it says it is “based on the number of significant hits […] identified by hmmsearch and MMSeqs2 […] divided by 100”. By “based on” do the authors mean “equals”? How do you handle the combination of the hits found by the two tools? This leaves the reader with a fuzzy view on this quantity crucial for the paper. I saw afterwards that the definition is provided as a small insert in Fig1. I suggest to make a proper definition clearly visible in the main text.

Response: We apologize for the lack of clarity in the previous version. To resolve this, we have revised the manuscript to clearly define how the scores were calculated. The V-score and V_L-score for each annotation profile within the KEGG, Pfam, eggNOG, PHROG, and VOG databases were determined based on the number of significant hits (E-value $\leq 10^{-5}$). These hits were identified using hmmsearch (HMMER 3.4) for the KEGG, Pfam, eggNOG, and VOG databases, and MMseqs2 specifically for the PHROG database. All searches were conducted against our constructed database of 18,435,589 protein sequences, as illustrated in Fig. 1. The updated text can be found in (lines 92–101, 411–415).

- I don’t see the point of providing a score and its logarithm. Why not one of the two? It over-complicates the message in my opinion as the reader feels that independent information is provided by the scores.

Response: We appreciate the opportunity to clarify the definitions and roles of our scoring metrics. The V-score is calculated by dividing the number of significant hits by 100, with the final value capped at a maximum of 10. The V_L-score is derived by scaling the number of hits using a base-10 logarithm (log10), with no upper limit applied. While both metrics quantify the prevalence and virus-likeness of protein families, they serve distinct purposes: the V-score is primarily utilized for identifying viral genomes, whereas the V_L-score is optimized for identifying auxiliary viral genes. We have therefore retained both to ensure the precision of our downstream analyses. Furthermore, to prevent overcomplicating the manuscript’s core message, we have removed “AV-score” and “AV_L-score” as distinct conceptual terms, instead using them only as abbreviations for “average V-score” and “average V_L-score” where necessary. This streamlines the terminology without loss of technical detail. The manuscript has been revised accordingly (lines 157–164).

- check for consitency innotations: VL vs V_L (e.g. L433 vs L417)

Response: Revised.

- There a confusion in the machine learning vocabulary in section 662+, which makes this hard to follow for the reader. Indeed a “true positive” is a **prediction** that turns out to be correctly predicted as a positive instance, whereas in the first paragraph of this section the authors employ this term to mean “we labeled” a specific gene as positive; which is very confusing.

Response: We apologize for the lack of clarity in the previous version. To address this, we have revised the manuscript (lines 686–707 and Fig. 5c,) and Supplementary Table S12 to clearly define our benchmarking dataset. We designated 17 experimentally verified AMGs as positive or negative controls (Supplementary Table S16) and 10 non-AMG genes as negative controls (Supplementary Table S17). These sets were then used to predict true positives, true negatives, false positives, and false negatives. The updated text provides a more rigorous framework for evaluating the workflow's performance.

- What do the authors mean by L673 “were removed before we predicted the positives and negatives”? How this is exactly done? I wonder if the authors used the fround thruth of the genome label to so, or if they used the prediction of “host” genome leveraging the threshold on the AL_L score? If the ground truth is used here, this comes with an unfair bias on the reported specificity.

Response: Apologize for the lack of clarity in the previous version. Because host genomes often contain integrated prophages that could confound our precision metrics, we employed geNomad

to identify viral contigs within the 180 host genomes. All genes encoded on scaffolds identified as viral were excluded from the analysis before determining the true positives and true negatives for our AMG identification workflow. This filtering step ensures that our benchmarking is restricted to genuine host-encoded sequences. The manuscript has been revised accordingly (lines 695–698) to clarify this procedure.

- Although I understand that the sensitivity is much reduced using a filter on viral hallmarks genes, I don't understand why the FDR is negatively impacted compared to the case where no post filtration os applied -- as shown on figure 4d? Shouldn't it be the other way around?

Response: Thank you for this observation. Our analysis in Supplementary Table S12 demonstrates that while requiring the presence of flanking "viral hallmark" genes reduced the number of false positives to 3, it also substantially decreased the number of true positives to 7. This suggests that a significant proportion of AMGs are not located in immediate proximity to hallmark genes. Consequently, while hallmark verification can serve as a strict filter, it also considerably reduces sensitivity and increases the False Discovery Rate (FDR) by excluding a large number of genuine AMGs. Based on these findings, we opted for a more balanced approach that maintains a higher recovery of true viral signals.

- No reference is given to figure described in paragraph L701+.

Response: Thank you for pointing this out. The figure described in the paragraph beginning at L701+ was provided in the supplementary material as Figure S11. For the sake of consistency and to improve the flow of the main text, we have also moved the corresponding methodological details to the Figure S11 legend in the supplementary files. The manuscript has been updated to reflect these changes.

- Fig6: No a. and b. on the figure can be found. I guess a. andb. Refers to repsectively left and right pannels.

Response: Thank you for your feedback. Figure number has been revised.

References:

[Perez-Bucio et al. 25] Perez-Bucio, R. ; Enault, F.; Galiez, C. EnVhogDB: an extended view of the viral protein families on Earth through a vast collection of HMM profiles. Peer Community Journal, Volume 5 (2025), article no. e100. https://doi.org/10.24072/pcjournal.627

POINT BY POINT RESPONSES TO REVIEWER COMMENTS

Reviewer #3 (Remarks to the Author):

I would like sincerely to thank the authors for their effort in answering my questions. Overall I am satisfied with the answers and the current status of manuscript, which provides much more quantitative information (precision-recall curves for instance) and more reasonable claims.

Response: Thanks for your positive comments.

I just would have a (last) request regarding the newly developed C-Score. As I understand, the authors average the clade-specific C-scores over all viral clades. By doing so, it washes out a potential signal. Indeed, many viral clades may be evolutionary completely independent and it does not make sense to expect a protein family to be present in all clades. In my opinion, such a C-Score should be only averaged over the clades containing at least one hit. Adding pseudo counts to correct for small protein families -- say, appearing in only one small clade -- may also be a good idea. Thereby the 2 first points raised by the authors concerning their C-score is not fully relevant with the comment above.

Response: We thank the reviewer for their interest in the C-score and for their thoughtful evaluation of our pipeline. Regarding the calculation of the C-score: we calculated the average by considering only the clades containing at least one hit. By focusing on active hits rather than all potential clades, our pipeline is designed to preserve, rather than dilute, potential signals. The specific number of clades used for these calculations is detailed in **Supplementary Tables S22–S26 (**We provide Supplementary Table S22-26 to the editor and reviewers if they wish to see these results (but do not wish to make these public due to the above mentioned issues).

C-score, like many database-dependent metrics, exhibit certain biases, including bias against underrepresented viruses and lower sensitivity for known markers. This is primarily a reflection of current database limitations, which are often biased toward cultured viruses and well-studied environments. Consequently, proteins from well-characterized viruses may receive higher scores than those from novel or uncultivated lineages. However, we anticipate that as viral databases continue to expand, the C-score will increasingly reflect the true diversity of the global virome.

Usability:
The tabulated data is correctly provided on the website, but I would suggest to:
- make a proper Zenodo (or similar) entry with this database
- add a proper link or a copy (if allowed) to the profiles/sequences mentioned in the database (with consistent sequence database version **and** matching identifier) so that the final users can quickly run a search and consolidate the results with the V-Scores provided in the table.
- provide simple script taking out the results of a standard profile search tool (Hmmer, MMSeqs for instance) and make the merge with the tabulated data. I agree that this is not a lot of work, but it can make the current work here much more used as soon as there is such an example provided.

Response: We thank the reviewer for these constructive comments. In response, we have updated the V-Score-Search website (https://anantharamanlab.github.io/V-Score-SearchTemp/) to enhance user accessibility and functionality. Additionally, we have established a permanent Zenodo entry (https://doi.org/10.5281/zenodo.18793980) for our database to ensure long-term data stability and traceability. To address the request for integration tools (e.g., for HMMER and MMseqs2), we have added a direct link to our computational pipeline on the main webpage, which can also be accessed via https://anantharamanlab.github.io/V-Score-SearchTemp/.

Minor:
- the y axis on figure 2 are not on the same scale that leads to misleading intuitions. I advise to use the same y-axis ranges to be able to compare more clearly the performance of each of the databases.

Response: Revised.
