# Supplementary material for: V- and VL-scores unveil viral signatures and origins of protein families
Source: Nat Commun. 2026 Apr 28;17:5828. doi: 10.1038/s41467-026-72028-0 (PMC13332234; doi:10.1038/s41467-026-72028-0)
Supplement: Supplementary file 25 — Transparent Peer Review file [file 41467_2026_72028_MOESM25_ESM.pdf]

nature portfolio

Corresponding author(s): Karthik Anantharaman, Kun Zhou

Last updated by author(s): Feb 28, 2026

Reporting Summary

Nature Portfolio wishes to improve the reproducibility of the work that we publish. This form provides structure for consistency and transparency in reporting. For further information on Nature Portfolio policies, see our Editorial Policies and the Editorial Policy Checklist.

Statistics

For all statistical analyses, confirm that the following items are present in the figure legend, table legend, main text, or Methods section.

n/a	Confirmed	
☐	☒	The exact sample size (n) for each experimental group/condition, given as a discrete number and unit of measurement
☐	☒	A statement on whether measurements were taken from distinct samples or whether the same sample was measured repeatedly
☐	☒	The statistical test(s) used AND whether they are one- or two-sided<br>*Only common tests should be described solely by name; describe more complex techniques in the Methods section.*
☐	☒	A description of all covariates tested
☐	☒	A description of any assumptions or corrections, such as tests of normality and adjustment for multiple comparisons
☐	☒	A full description of the statistical parameters including central tendency (e.g. means) or other basic estimates (e.g. regression coefficient) AND variation (e.g. standard deviation) or associated estimates of uncertainty (e.g. confidence intervals)
☐	☒	For null hypothesis testing, the test statistic (e.g. F, t, r) with confidence intervals, effect sizes, degrees of freedom and P value noted<br>*Give P values as exact values whenever suitable.*
☒	☐	For Bayesian analysis, information on the choice of priors and Markov chain Monte Carlo settings
☒	☐	For hierarchical and complex designs, identification of the appropriate level for tests and full reporting of outcomes
☒	☐	Estimates of effect sizes (e.g. Cohen's d, Pearson's r), indicating how they were calculated

Our web collection on statistics for biologists contains articles on many of the points above.

Software and code

Policy information about availability of computer code

Data collection	No software was used for collection.
Data analysis	Perl and Python scripts were used to analyze the data in this study. Codes used in this project are available at https://github.com/AnantharamanLab/V-Score-Search.<br><br>List of software: Bowtie2 (version 2.3.4), CheckV(version 1.0.13), DeepVirFinder (version 1.0), DRAM-v (version 1.5.0), EggNOG-mapper (version 2.1.12) , geNomad (version 1.7.411), hmmsearch (HMMER 3.4), MEGAHIT (version 1.2.9), MMseqs2 linclust (version 13.45111), Prodigal (version 2.6.3), pyhmmer (version 0.10.10), pyrodigal-gv (version 0.3.1), R package gggenomes (version 1.0.0), SAMtools (version 1.6), SPAdes (version 3.11.1), Trimmomatic (version 0.36), VIBRANT (version 1.2.0), VirSorter2 (version 2.2.3).

For manuscripts utilizing custom algorithms or software that are central to the research but not yet described in published literature, software must be made available to editors and reviewers. We strongly encourage code deposition in a community repository (e.g. GitHub). See the Nature Portfolio guidelines for submitting code & software for further information.

Data

Policy information about availability of data

All manuscripts must include a data availability statement. This statement should provide the following information, where applicable:

- Accession codes, unique identifiers, or web links for publicly available datasets
- A description of any restrictions on data availability
- For clinical datasets or third party data, please ensure that the statement adheres to our policy

A database of V-scores and VL-scores associated with every protein cluster or family is available at https://anantharamanlab.github.io/V-Score-Search/. Reference sequences utilized for evaluation can be downloaded from IMG/VR (https://genome.jgi.doe.gov/portal/IMG_VR/IMG_VR.home.html). Metagenome reads of host-associated microbiomes were retrieved from NCBI (https://www.ncbi.nlm.nih.gov/).

Research involving human participants, their data, or biological material

Policy information about studies with human participants or human data. See also policy information about sex, gender (identity/presentation), and sexual orientation and race, ethnicity and racism.

Reporting on sex and gender	n/a
Reporting on race, ethnicity, or other socially relevant groupings	n/a
Population characteristics	n/a
Recruitment	n/a
Ethics oversight	n/a

Note that full information on the approval of the study protocol must also be provided in the manuscript.

Field-specific reporting

Please select the one below that is the best fit for your research. If you are not sure, read the appropriate sections before making your selection.

☒ Life sciences ☐ Behavioural & social sciences ☐ Ecological, evolutionary & environmental sciences

For a reference copy of the document with all sections, see nature.com/documents/nr-reporting-summary-flat.pdf

Life sciences study design

All studies must disclose on these points even when the disclosure is negative.

Sample size	We assessed the quality of each genome with a quality score (score = completeness − 5 × contamination − 0.05 × no. scaffolds), genomes of each GTDB family with the highest quality score were selected as family representatives to reduce computational load and taxonomic bias. As a result, 4,304 bacterial and 509 archaeal genomes were selected to be used in analyses. Additionally, the genomes of plasmids (n = 50,523) and prokaryotic viruses (n = 5,800) in publicly available databases for the comparison of AV-score and AVL-score between viruses, plasmids, and chromosomes were collected to unveil viral signatures.
Data exclusions	No data were excluded.
Replication	False positives are defined as prokaryotic sequences that were erroneously identified as viral sequences based on the AV-score approachs (AV-score alone or combined with CheckV). False negatives are viral sequences that were not identified as viral sequences using the AV-score approachs (AV-score alone or combined with CheckV). To assess these rates, we randomly selected 100 prokaryotic and 100 viral sequences and calculated the false negative and false positive rates. This randomization and calculation were repeated 1000 times using a bootstrap resampling method.
Randomization	Each whole genome of prokaryotic viruses, plasmids, and chromosomes were randomly split into non-overlapping, non-redundant genome fragments at length from 1 to 15kb to simulate metagenome-assembled sequences.
Blinding	No blinding was performed because it does not affect analysis. Investigation was conducted using the databases of prokaryotic chromosomes, plasmids, and prokaryotic viruses and they were kept separated.

Reporting for specific materials, systems and methods

We require information from authors about some types of materials, experimental systems and methods used in many studies. Here, indicate whether each material, system or method listed is relevant to your study. If you are not sure if a list item applies to your research, read the appropriate section before selecting a response.

Materials & experimental systems

n/a	Involved in the study
☒	☐ Antibodies
☒	☐ Eukaryotic cell lines
☒	☐ Palaeontology and archaeology
☒	☐ Animals and other organisms
☒	☐ Clinical data
☒	☐ Dual use research of concern
☒	☐ Plants

Methods

n/a	Involved in the study
☒	☐ ChIP-seq
☒	☐ Flow cytometry
☒	☐ MRI-based neuroimaging

Plants

Seed stocks	n/a
Novel plant genotypes	n/a
Authentication	n/a
